# Peroxidase-induced C–N bond formation *via* nitroso ene and Diels–Alder reactions†

**DOI:** 10.1039/d2gc04827b

**Published:** 2023-03-21

**Authors:** Christina Jäger, Bernhard J. Gregori, Juhana A. S. Aho, Marleen Hallamaa, Jan Deska

**Affiliations:** a Department of Chemistry, University of Helsinki A.I. Virtasen aukio 1 00560 Helsinki Finland jan.deska@helsinki.fi https://www.deskalab.com; b Institut für Anorganische und Angewandte Chemie Martin-Luther-King-Platz 6 20146 Hamburg Germany

## Abstract

The formation of new carbon–nitrogen bonds is indisputably one of the most important tasks in synthetic organic chemistry. Here, nitroso compounds offer a highly interesting reactivity that complements traditional amination strategies, allowing for the introduction of nitrogen functionalities *via* ene-type reactions or Diels–Alder cycloadditions. In this study, we highlight the potential of horseradish peroxidase as biological mediator for the generation of reactive nitroso species under environmentally benign conditions. Exploiting a non-natural peroxidase reactivity, in combination with glucose oxidase as oxygen-activating biocatalyst, aerobic activation of a broad range of *N*-hydroxycarbamates and hydroxamic acids is achieved. Thus both intra- and intermolecular nitroso-ene as well as nitroso-Diels–Alder reactions are performed with high efficiency. Relying on a commercial and robust enzyme system, the aqueous catalyst solution can be recycled over numerous reaction cycles without significant loss of activity. Overall, this green and scalable C–N bond-forming strategy enables the production of allylic amides and various N-heterocyclic building blocks utilizing only air and glucose as sacrificial reagents.

## Abstract 2

Biocatalytically activated *N*-hydroxy carbamates and hydroxamic acids undergo C–N bond forming reactions through ene-type reactions or Diels–Alder cycloadditions *via* the generation of reactive acylnitroso intermediates.

## Graphical abstract

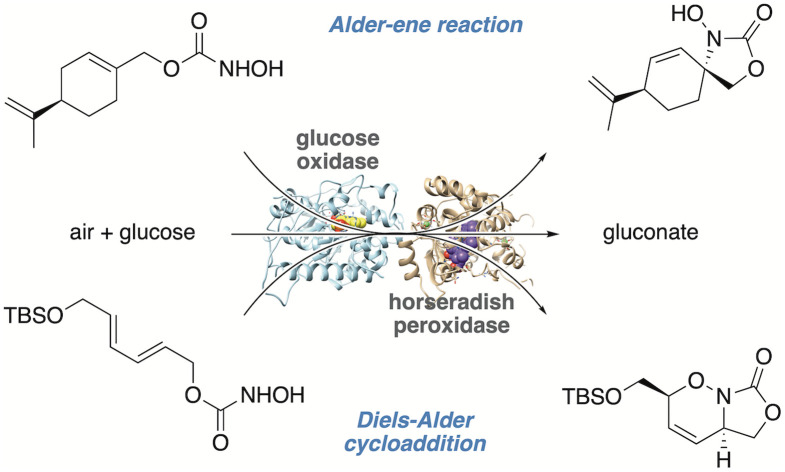

## Introduction

The incorporation of nitrogen into organic building blocks through C–N bond forming reactions represents one of the most crucial methodology goals in modern organic chemistry.^1^ Especially nitrogen-containing heterocycles, an absolute go-to motif in today's pharmaceuticals,^2^ pose a highly important synthetic target. Among the various different ways to conduct carbon–nitrogen bond formations, nitroso reactions occupy something of a niche.^3^ Nevertheless, their distinctive reaction profile with a high affinity for ene and diene reaction partners also puts nitroso species in a unique position where they complement more traditional methodologies such as condensations or cross coupling reactions in offering an alternative pre-functionalization strategy. First explored in the 1940s, nitroso-Diels–Alder cycloadditions and nitroso-ene reactions later in the 1960s are nowadays found as key transformations in a large number of total syntheses yielding natural products and pharmaceuticals.^4–6^ While there is a broad range of ways to *in situ* access the highly reactive, electrophilic nitroso intermediates, in particular the oxidation of hydroxylamine derivatives has been established as common general approach. Here, both stoichiometeric oxidants such as hypoiodites,^7^ iodosoarenes^8^ and amine *N*-oxides^9^ have found application, as well as transition metal catalysts^10–13^*en route* to use milder and chemically more benign oxidizing agents.

**Scheme 1:**
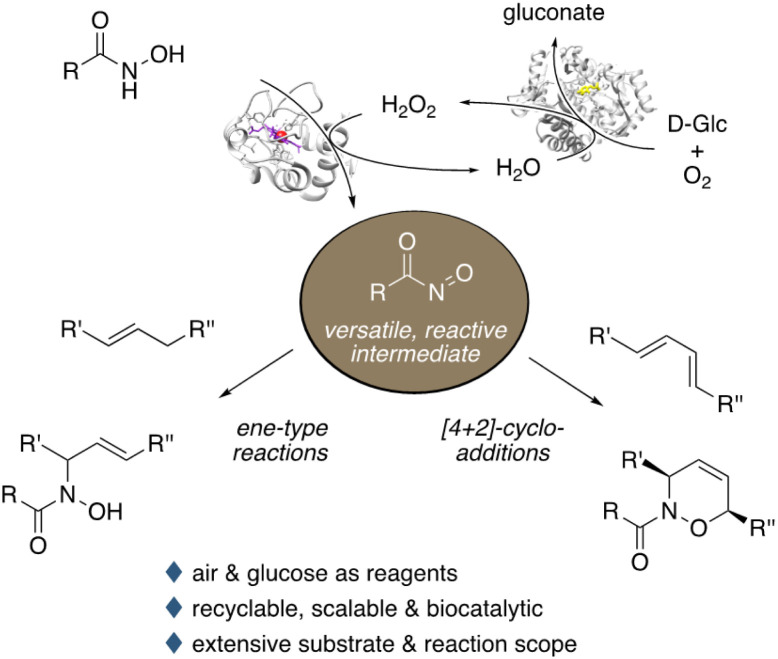
Biocatalytic generation and reactions of nitrosocarbonyl species using horseradish peroxidase and glucose oxidase.

In our ongoing endeavour to discover new enzymatic tools that are inspired by traditional organic chemistry,^14^ we herein disclose a generally applicable, purely biocatalytic route to conduct nitroso-ene-type reactions and nitroso-Diels–Alder cycloadditions (Scheme 1). The combination of a peroxidase and glucose oxidase renders a highly effective dehydrogenation system that utilizes only glucose and air as stoichiometric sacrificial reagents.^15^ The extensive study covers insights into the method development, the substrate scope of both intra- and intermolecular ene-type and Diels–Alder reactions, upscaling, as well as catalyst recycling.

## Results and discussion

The high reactivity of nitrosocarbonyls makes them useful intermediates in a range of C–N bond forming reactions. Traditional methods for their *in situ* generation based on hydroxylamine derivatives, however, usually rely on the use of stoichiometric amounts of strong oxidants. Among the transition metal-mediated protocols, particularly the use of simple iron catalysts with hydrogen peroxide caught our attention.^13^ In our attempt to mimic this reactivity, assuming that iron-dependent heme enzymes may offer a similar activation pathway, we investigated a bi-enzymatic system for its ability to catalyze a nitroso-ene-type cyclization by oxidative activation of *N*-hydroxycarbamate 1a (Table 1). Here glucose oxidase serves as catalyst to provide hydrogen peroxide by reduction of oxygen in order to fuel different peroxidases. Their oxidized heme centre should accept electrons from the hydroxylamine substrates and thus deliver the reactive key intermediate, the acylnitroso species.^16^ While fungal peroxygenase, lactoperoxidase and chloroperoxidase only converted the model substrate 1a very slowly, horseradish peroxidase (HRP) showed an exquisite performance, quickly converted 1a and led to the selective formation of the oxazolidinone 2a in 97% yield (Table 1, entry 1), and the system tolerated both water-miscible and non-miscible cosolvents really well (Table 1, entries 2 & 3). Under these previously optimized conditions, both phosphate and glucose represent the main contributors to the overall waste. As such, we tried to increase the substrate concentration and at 50 mM (*i.e.* with only 2 equivalents of phosphate) an acceptable yield of 70% was achieved, however at the expense of significantly slower conversion rates (Table 1, entry 4). Reduction of the glucose loading on the other hand proved to be more viable and the excellent outcome of the original protocol was maintained (Table 1, entry 5).

**Table 1:** Peroxidase screening for the ene-type cyclization of 1a

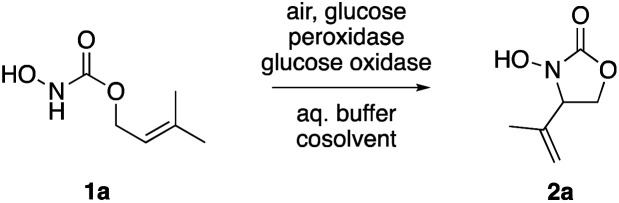					
#	Glucose [equiv.]	1a [mM]	Cosolvent^a^	Time [h]	Yield^b^ [%]
1	5.0	10	—	2	97
2	5.0	10	EtOAc	2	96
3	5.0	10	Dioxane	2	94
4	5.0	50	—	7	70
5	1.2	10	—	2	98

a 10 vol%.

b Isolated yields of 2a.

**Fig. 1:**
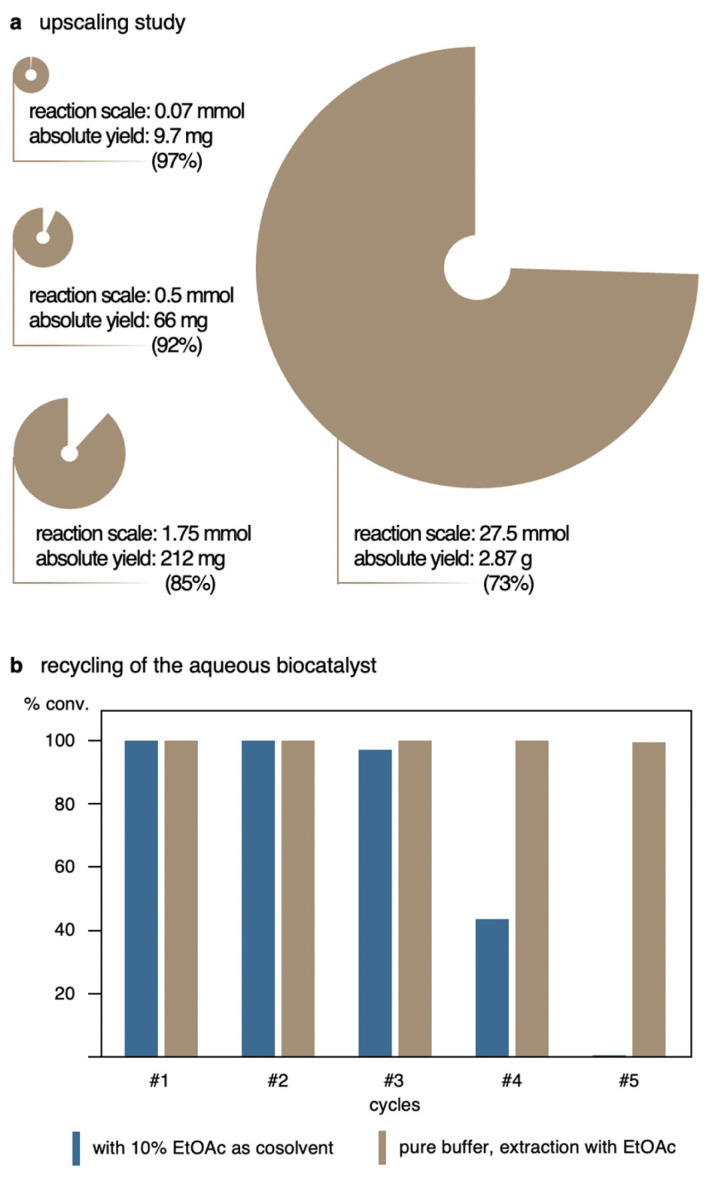
Upscaling & recycling studies for the intramolecular nitroso-ene reaction using substrate 1a. (a). The HRP/GOx system allows for a multi-gram scale-up. (b). While the use of ethyl acetate as cosolvent (10% v/v) results in loss of activity after three cycles of catalysis, recycling by extraction from pure buffer media maintains excellent biocatalytic performance over multiple reaction cycles.

To assess the practical synthetic potential of the method, the ene-type cyclization of 1a was incrementally scaled up while otherwise maintaining the original optimized conditions (Fig. 1a and ESI Table 2†). The reaction was first performed in a total reaction volume of 50 mL yielding promising 92% of product 2a. At 175 mL, representing a 25-fold scale-up compared to the original volume, however minor deterioration of efficacy of the system became and 2a was obtained in 85% yield. Nevertheless, in either case, the reaction time remained unaffected by these changes and full conversion was reached after 2 h. Performing the nitroso-ene reaction in a gram scale, *i.e.* starting with 4.0 g of *N*-hydroxycarbamate 1a while proportionately increasing also reagent, catalyst and solvent volumes, a slower conversion was observed and the reaction required 24 h to reach completion. Even though the biotransformation still proceeded cleanly, with no apparent side products being detected, the overall yield of 2a remained somewhat lower, with 73%. At almost 3 L of reaction medium, however, it does not really surprise that transport processes and proper aeriation would take are more pronounced role and that oxygen saturation could become a limiting factor. This, on the other hand, would be easily solved at a later stage by moving from the normal lab-scale beakers to reactor setups that allowed for the control of oxygen consumption.

As process engineering aspects of that kind have not been part of our ongoing study, we turned our attention to an alternative strategy to improve the efficacy and scale of the biotransformation. To circumvent the previously discussed sustainability issues related to low substrate concentrations, the idea of recycling of the aqueous enzyme solution arose, making use of the highly effective and fast small-scale system while at the same time utilizing the robust HRP/GOx enzyme cocktail for multiple rounds of catalysis (Fig. 1b). Since the ene-type biotransformation of 1a showed high tolerance to the green organic cosolvent ethyl acetate, we envisaged that a biphasic setup would allow to charge the reaction with the ene substrate as a solution in the organic cosolvent, and upon completion to simply remove the product through separation of the layers. Replacement with fresh EtOAc containing a new portion of the ene substrate would exploit the remaining biocatalyst-buffer solution for subsequent biotransformations. One challenge in peroxidase-mediated reactions can be the bleaching of the heme enzymes by high concentrations of hydrogen peroxide.^17^ Thus, the amount of d-glucose was adjusted to only 1.2 equivalents relative to 1a to produce sufficient but not excessive H_2_O_2_. With each new cycle, not just fresh substrate in EtOAc was added, but also new d-glucose was delivered expecting to result in more reaction cycles. While the recycling experiments with 10% (v/v) of EtOAc as cosolvent first seemed promising, reaching full conversion to yield 2a in quantitative yields in the initial rounds of biocatalysis, a significant impairment of the enzyme couple became apparent during the fourth cycle and the conversion of 1a could no longer exceed 47%. It is known that organic solvents can affect the stability of an enzyme by lurking through the protein structure. For HRP this case was studied, finding that hydrogen bonds in the active site can be disrupted under these circumstances and lead to unfolding of the enzyme. Subsequent release of the iron-containing prosthetic group as consequence renders the protein inactive. However, this was so far observed for fairly high cosolvent concentrations of >50% (v/v) and prolonged incubation times.^18^ To rule out a negative impact by the cosolvent, repetitive recycling of the aqueous HRP/GOx system was also tested in absence of any cosolvent, and a minimal amount of EtOAc was only employed for product extraction after reaching full conversion. Gratifyingly, the extraction using EtOAc did not disturb the reaction outcome, suggesting that the inactivation just happens if exposed for longer time periods. Recycling of the aqueous enzyme solution with repetitive substrate/glucose addition and product extraction worked flawlessly over the duration of a full work day, accounting for five cycles of catalysis with full conversion to the ene-cyclized oxazolidinone 2a.

Enzymatic reactions are often characterized as highly effective in terms of their catalytic turnover. While this is true for the conversion of native substrates, the application of biocatalysts in non-natural reactions can sometimes be hampered by reduced total turnover numbers. Quite contrary, the HRP-mediated cyclization of 1a proceeds with exquisite efficacy, reaching a total turnover number of >217 000 (relative to the catalytically active iron porphyrin centre), while its chemical blueprint using iron(iii) chloride operated in a range of maximum 23 catalytic turnovers.^13^

**Scheme 2:**
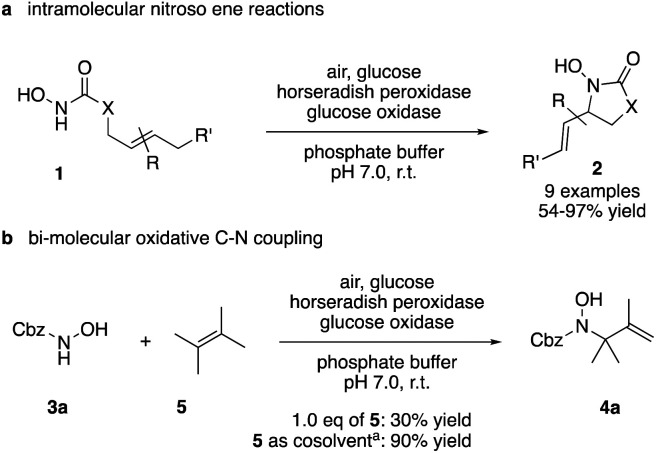
Scope and limitations: while cyclization reactions proceed with high efficiency (a), intermolecular C–N couplings require a significant excess of the ene reaction partner (b). ^*a*^ 10% v/v.

Apart from the promising catalytic performance and the benefits of scalability and recycling, the peroxidase-induced ene-type reaction also exhibited a broad tolerance regarding the scope of accepted olefin-functionalized hydroxylamines in the intramolecular C–N coupling based on the HRP/GOx activation.^16^ As reported in our preliminary study, a wide range of heterocycles could be obtained in moderate to very good yields by employing *N*-hydroxycarbamates and hydroxamic acids in the biocatalytic cyclization protocol (Scheme 2). The intermolecular coupling, however, has remained far from optimal, since the best results were reached by utilizing the olefinic coupling partner as cosolvent. Applying the previously introduced protocol, *N*-hydroxy benzyl carbamate (3a) was coupled to tetramethylethylene (5, used as cosolvent, 10% v/v) to yield 90% of product 4a within 2 h. Any attempts to work with equimolar or moderately superstoichiometric concentrations of olefin, with EtOAc as cosolvent, did not lead to satisfying yields but always proceeded in full conversion of substrate 3a (ESI Table S1†). Clearly, further refinement of the protocol was necessary with regard to the reaction's efficiency.

In an unrelated study investigating enzymatic allene halogenations, we discovered a positive influence of detergents on biphasic peroxidase-induced biotransformations.^19^ So we were very delighted to observe that this previously developed solvent system, consisting of an aqueous buffer, and Brij 35 as non-ionic emulsifier for the hydrocarbon cosolvent, substantially improved the ene-type coupling between 5 and 3a. With only a moderate excess of the olefin, the intermolecular coupling product 4a could be obtained in an excellent yield of 93% after only 45 min of incubation (Table 2, entry 1). Moreover, the methodology exploiting emulsion biocatalysis extended well to other highly substituted olefins, and amylene (6) and prenol (7) were coupled with 3a to give rise to the *N*-functionalized carbamates in respectable yields of 55% and 77%, respectively (Table 2, entries 2 & 3). On the other hand, the cyclic olefins 8 and 9 performed only poorly and in order to reach significant product formation, we needed to resort back to the cosolvent solution (Table 2, entries 4 & 5). The amidated cyclohexenes 4d and 4e were thus obtained in moderate yields of 33% and 26%, respectively, and the 6 : 1 *twix*/*twin* ratio in 4e is consistent with typical selectivity patterns of these substrates.^12^

**Table 2:** Intermolecular oxidative coupling of carbamate 3a with olefins

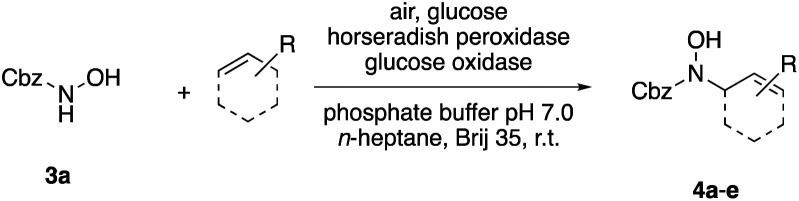			
#	Olefin	Product (s)	Yield^a^ [%]
1	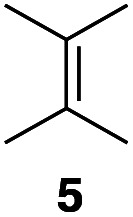	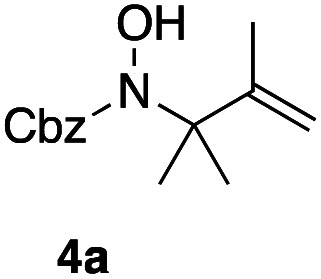	93
2	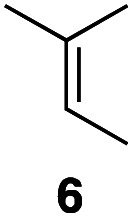	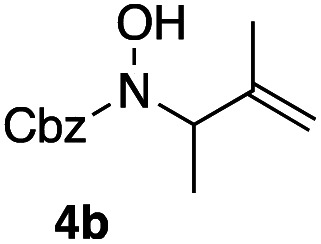	55
3	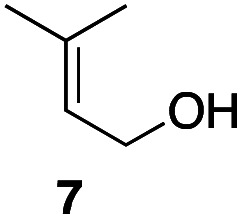	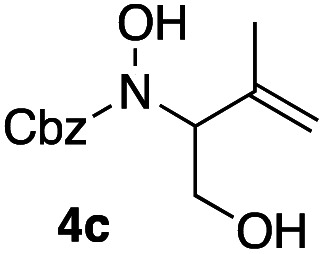	77
4^b^^,^^c^	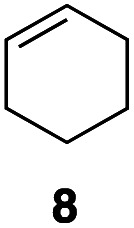	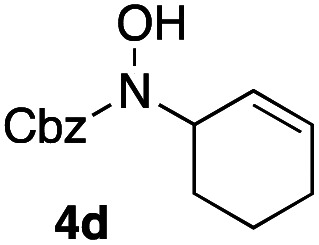	33
5^b^^,^^c^	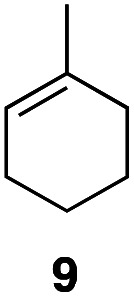	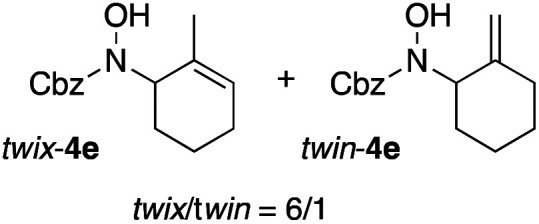	26

a Isolated yields.

b Olefin as cosolvent.

c ^1^H-NMR yields using dimethylsulfone or mesitylene as internal standard.

The ene reaction is just one great way to utilize the highly reactive nitroso intermediates in C–N bond forming reactions. After the successful development of an enzyme-driven methodology that works both in intra- and intermolecular nitroso ene-type reactions, we turned our attention to the second reactivity pattern of the N=O derivatives, that is, their ability to act as dienophiles in hetero Diels–Alder reactions. Diels–Alder cycloadditions have for a long time been a topic of interest in enzyme catalysis development and over the years, both native Diels–Alderases^20^ and artificial biocatalytic tools for [4 + 2]-cycloadditions have been discovered.^21^ We used 1,3-cyclohexadiene (10) as benchmark reaction partner and assumed, considering the similarities to cyclohexene, that the emulsified medium would also offer advantages in the [4 + 2]-cycloaddition. In fact, the desired bicyclic product 11a was obtained in good yield of 63% (Table 3, entry 1), however, even better results were accomplished in absence of the amphiphile, in a more classical two-phase cosolvent system (Table 3, entries 2 & 3). This prompted us to re-investigate the solvent influence, where ethyl acetate, the optimal cosolvent for intramolecular ene reactions, yet again emerged as effective additive (Table 3, entry 4), and at 20% v/v an excellent yield of 90% was achieved. In contrast, toluene as other non-miscible cosolvent or any of the tested miscible cosolvents failed to provide good results in the cycloaddition between the 3a-derived nitroso derivative and 10 (Table 3, entries 6–11). Notably, the reaction parameters were slightly adjusted for the nitroso-Diels–Alder reactions to a 5 : 1 HRP/GOx ratio yielding a slower release of hydrogen peroxide (ESI Fig. 2†).

**Table 3:** Medium optimization for the peroxidase-induced intermolecular nitroso-Diels–Alder reaction

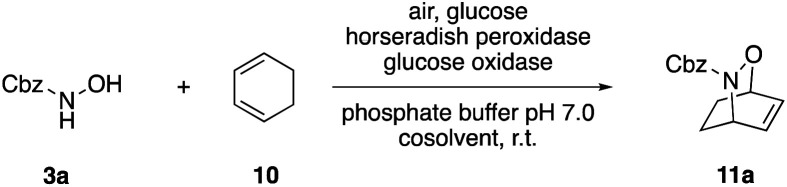				
#^a^	Cosolvent	[% v/v]	Time [h]	Yield [%]
1	*n*-Heptane/Brij 35	10	2	63
2	*n*-Heptane	10	2	76
3		20	1	82
4	EtOAc	10	2	47
5		20	1.5	90
6	Toluene	10	4	38
7	Dichloromethane	10	22	<5
8	Dioxane	10	3	13
9	DMSO	10	4	13
10	*t*-BuOH	10	4	10
11	MeCN	10	4	12
12^b^	None	—	6	12

a ^1^H-NMR yields using dimethylsulfone or mesitylene as internal standard.

b 70 U GOx.

**Scheme 3:**
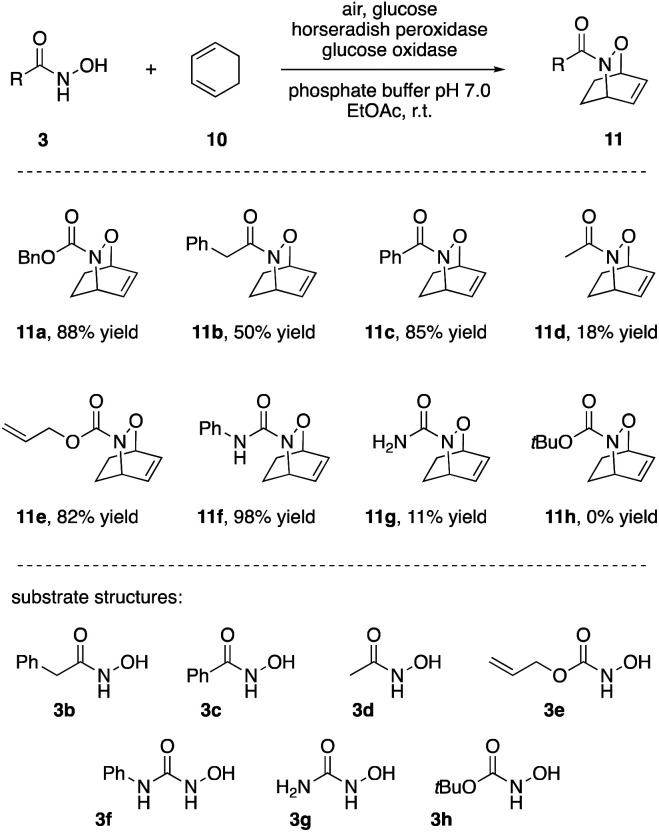
Hydroxamic acids, *N*-hydroxycarbamates and *N*-hydroxyureas as nitroso precursors in intermolecular [4 + 2]-cycloadditions.

The synthetic applicability of the HRP-induced [4 + 2]-cycloaddition was subsequently tested, both through modification of the diene and dienophile reaction partners. First, changing the acylation pattern at the hydroxylamine, mainly good to excellent yields of the bicyclic products were obtained (Scheme 3). Interestingly, unsaturated acyl groups at the nitrogen seemed much more beneficial than saturated ones. Benzamide 3c outperformed acetamide 3d to a similar extent (85% *vs.* 18%) as *N*′-phenylurea 3f outperformed urea 3g (98% *vs.* 11%). So far, this phenomenon was not observed in the nitroso Diels–Alder literature and may be connected to the interaction of the substrate with the active site of the enzyme. Indeed, studies of HRP-C, the most abundant isoenzyme of HRP, revealed the structural feature of an aromatic binding pocket explaining the bias in favour of recognizing small aromatic molecules (and stabilizing the thus formed nitroso intermediates). Due to substrate interactions with phenylalanine residues in the access channel of the enzyme, the substrate-protein binding improves by forming a “closed lid” conformation which may explain the distinct phenomena in our studies.^22^ As opposed to the scale-up example where lower yields were likely caused by poor supply of the medium with air as terminal oxidant, deterioration of yields for slowly reacting substrates can be attributed to the relatively low stability of acylnitroso species in the aqueous solvent. With a half-life of a few minutes, the oxidatively activated N=O species hydrolyse quickly if trapping with the desired ene or diene compound is delayed.^18^ Yet more importantly from the synthetic perspective, a set of commonly used *N*-protective groups (Cbz, Bz, Alloc) were well tolerated and gave good to excellent yields of the bicyclic N,O-heterocycles.

**Scheme 4:**
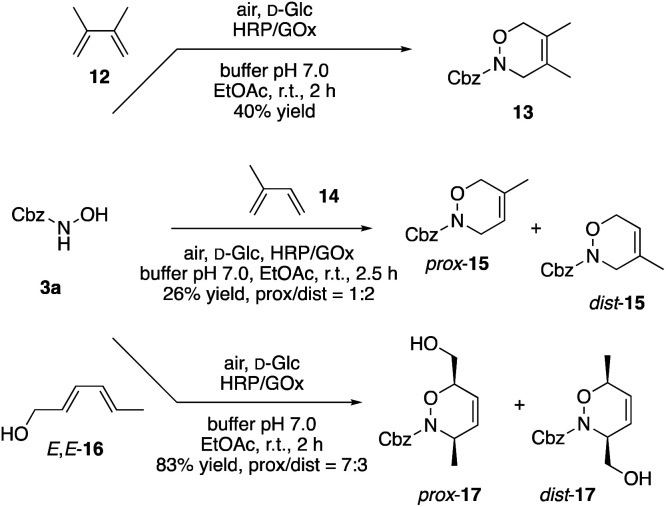
Intermolecular nitroso-Diels–Alder reaction *via* HRP/Gox-mediated dehydrogenation.

The synthetically more interesting aspect of nitroso-Diels–Alder reactions certainly lies in the structural scope regarding the diene reaction partner. At the same time, moving away from cyclic dienes like 10 renders the cycloadditions more challenging as the additional rotational freedom along the dienes’ single bond does no longer lock the substrate in the required s-*cis* conformation. Whiting *et al.* had studied acyl nitroso compounds in [4 + 2] cycloadditions with the goal to understand their behaviour. They concluded that the reaction time plays a crucial factor due to the competitive decomposition of the very reactive nitroso species.^23^ Considering the prevalent s-*trans* conformation of substrates such as 12 or 14, extended reaction times and hydrolysis of the 3a-derived nitroso compound could likely explain the reduced yields of the cycloaddition products 13 and 15 (Scheme 4). By reacting just 3a under the same reaction conditions without adding any reaction partners, conversion was observed also in our case supporting the possibility of decomposition due to the highly reactive character of the nitroso intermediate (ESI Table 2†). On the other hand, intermolecular [4 + 2] cycloadditions might be facilitated by improving the transport of the lipophilic diene substrate to the nitroso intermediate that is formed in the aqueous layer. Hence, sorbic alcohol (16) with its significantly increased solubility in water performed well in the bimolecular nitroso-Diels–Alder reaction. With a regioselectivity of 70 : 30 in favour of the proximal isomer, the cycloaddition products 17 were isolated in a very good overall yield of 83%. Even more importantly, the products were obtained as pure *cis*-diastereomers, indicating that the cycloaddition indeed proceeds through a pericyclic, concerted pathway.

**Scheme 5:**
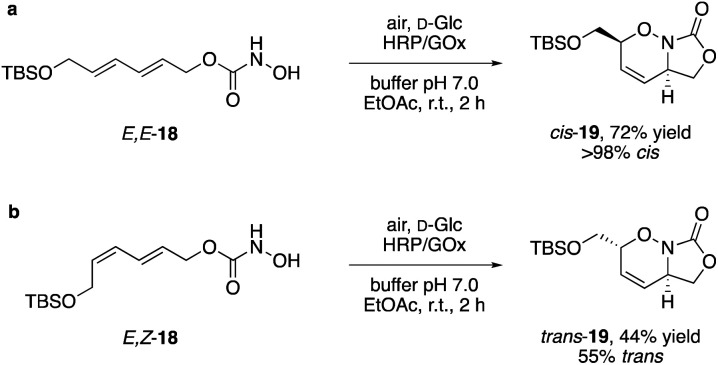
Synthesis of bicyclic N,O-heterocycles through intramolecular [4 + 2]-cycloaddition.

The same stereospecificity, that is typical for the pericyclic Diels–Alder reactions, was also observed in the intramolecular [4 + 2] cycloadditions of *E*,*E*-18, and the bicyclic N,O-heterocycle 19 was isolated as pure *cis*-isomer in 72% yield (Scheme 5). In contrast, while the *E*,*Z*-isomer reacted under identical conditions also in a Diels–Alder fashion, the diastereoselectivity of the transformation plummeted. However, it was observed before that *E*/*Z*-dienes can yield mixtures of *cis*- and *trans*-isomers, while *E*/*E*-dienes gave the pure *cis*-compound.^24^ Mechanistic studies revealed that the nitroso-Diels–Alder reaction proceeds in an asynchronous concerted mechanism, which arises from electrostatic repulsions between lone pairs of the nitroso compound and the diene and certainly can result in eroded selectivity.^25^

### Sustainability assessment

The use of enzymes is by no means automatically resulting in a better environmental footprint or render any kind of reaction green.^26^ It is true, however, that the commonly employed reagents, and water as reaction medium, may often provide benefits in terms of toxicity or biodegradability. In our sustainability assessment, we will compare the herein presented and optimized benchmark reaction for the synthesis of 2a with state-of-the-art methodologies utilizing traditional stochiometric oxidants as well as metal-mediated catalytic processes. The former utilizes diacetoxyiodobenzene as oxidant in a solvent mixture of dichloromethane and DMF,^8^ the latter systems consist of either iron(iii) chloride as catalysts with hydrogen peroxide as oxidant (in isopropanol at 100 °C),^13^ or copper(i) chloride as catalyst with air as oxidant (in THF at r.t.).^12^ In addition to a qualitative assessment of individual reaction components, also a comparative analysis of *E*-factors was considered useful to identify sustainability advantages but also reveal development targets for potential process-related optimizations.

Apparently, the traditional method suffers from major shortcomings in terms of green chemistry principles, as it relies on both chlorinated solvents and a high molecular weight halogenated oxidant. In terms of safer solvent systems, THF is certainly not without its own problems and generally classified as problematic whereas both isopropanol or our aqueous reaction medium score as recommended in common solvent selection guides. When it comes to the oxidation reagents and the resulting waste, similarly, halogenated aromatics pose a major issue and in comparison, the gluconate formed during the enzyme-mediated nitroso reactions can be characterized as less hazardous and fully biodegradable. Here, the two metal-catalyzed reactions offer the best profile with only water being formed as waste.

**Fig. 2:**
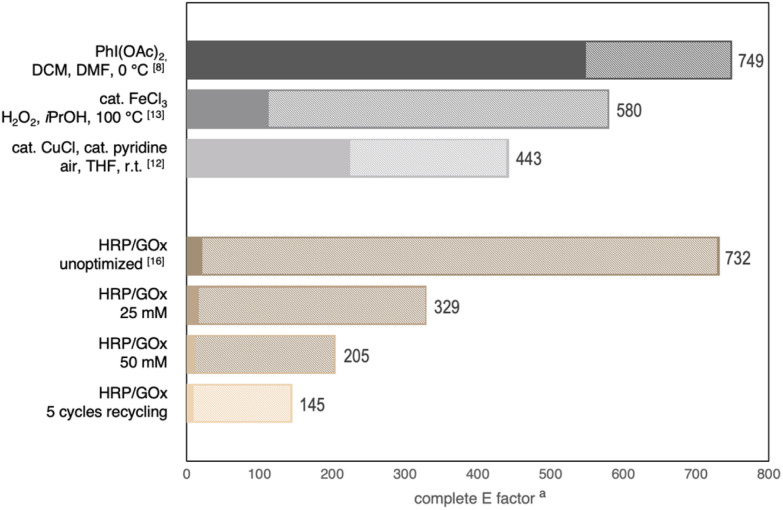
Quantitative analysis of the overall waste intensity of nitroso-ene reactions of 1a. The contribution of water or aqueous solutions is marked as hatched area. ^*a*^ To avoid a disproportionate positive bias for the HRP-based cyclizations, extraction solvents were excluded from the *E* factor calculations for all reactions.^27^

Less obvious to judge and sometimes misleading, however, are factors relating to efficiency, atom economy and the amount of waste that results from a given transformation. Particularly biocatalytic processes often suffer from the need to conduct reactions at relatively low substrate concentrations. Hence, an overall good sustainability footprint can be diminished by a poor *E*-factor that expresses the ratio of all waste relative to the amount of product. And when assessing the above mentioned protocols for oxidative nitroso ene-type reactions, in fact, the initially identified HRP-mediated oxidation at 10 mM substrate concentration reaches the same 700+ *E*-factor as the method based on hypervalent iodine (Fig. 2). At increased substrate concentrations (25 mM and 50 mM) in the enzymatic oxidation, this atom efficiency measure substantially improves even though quantitative yields are no longer achieved. In the overall most efficient setup, the previously described recycling of the aqueous catalyst solution (with five biotransformation rounds catalyzed by the same batch of enzyme solution), the HRP-system outperforms the copper-catalyzed protocol by a factor of three reaching an *E*-factor of 145.^27^

It is worth noting that the major contribution to the biocatalytic transformations’ *E*-factors stems from water (>97%), with phosphate (2 eq. per mole product) and gluconate (1.2 eq. per mole product) as main solutes. Compared to the reference protocols washing solutions (aq. thiosulfate, aq. hydrochloric acid, and aq. EDTA, respectively), this waste stream can certainly be characterized as more environmentally benign, yet, particularly the use of phosphorous-based chemicals has to be seen as an issue that needs to be addressed more broadly.^28^ It seems therefore of general interest to identify alternatives to phosphate buffers in the neutral pH range that would fulfil critical sustainability criteria. Certain amino acids may offer a better profile, and we were pleased to find that the benchmark cyclization of 1a proceeded similarly well in a neutral histidine buffer (97% yield, 2 h). The issue with the relatively high molecular weight of the reductant, glucose, could unfortunately not be fully addressed within this study. Attempts to directly fuel the oxidations with aqueous hydrogen peroxide instead of d-Glc/GOx did in fact lead to product formation but the reactions were markedly disturbed and could not reach conversions above 50%. Here, alternative peroxidases with a higher peroxide tolerance may play an important role towards more atom-economic protocols. The use of glucose and phosphate as sole stoichiometric waste chemicals, however, also poses a preparative advantage. Since the benchmark reaction reaches full conversion of the substrate and all side products and additives remain highly soluble in the aqueous layer, a simple extraction with ethyl acetate yields analytically pure product, rendering chromatographic and solvent-intensive purification steps obsolete.

## Conclusions

In summary, the newly developed biocatalytic system for the dehydrogenative activation of nitrosocarbonyls has proven to be a valuable and versatile synthetic tool for C–N bond forming reactions. The combination of horseradish peroxidase and glucose oxidase, with only air and glucose as stoichiometric reagents, offers an environmentally friendly way to generate acylnitroso compounds that can engage *in situ* in ene-type reactions and [4 + 2] cycloadditions, two transformations that are highly desirable in organic synthesis yet generally unknown in biosynthesis. The application of the wild-type natural catalysts in new-to-nature reactions does not only proceed with high yields but the over catalytic system exhibits an exquisite efficacy, with total turnover numbers above 200 000. With a very wide substrate tolerance the HRP/GOx-mediated methodology enables C–N bond formations in both inter- and intramolecular fashion and thus offers a broad product range from simple allylic amine derivatives to highly complex and stereochemically defined bicyclic N,O-heterocycles. Most transformations are best performed in a biphasic reaction system where the aqueous medium is supplemented with ethyl acetate as sustainable and environmentally acceptable choice for an organic cosolvent. Moreover, the biphasic setup effectively protects the enzymatic system and expedites upscaling to reaction volumes of several litres. Likewise, the combination of water and ethyl acetate facilitates catalyst recycling so that the aqueous biocatalyst solution can be recovered and reused in multiple rounds of catalysis without significant loss of efficacy.

## Experimental

### General methods & materials

Commercially available reagents were used without further purification. glucose oxidase (lypophilized powder, type II, 158 200 U g^−1^, *Aspergillus niger*) and horseradish peroxidase (lypophilized powder, beige, 173 U mg^−1^) were obtained from Sigma Aldrich. All reactions were carried out under argon atmosphere and performed with dry solvents if not stated differently. All enzymatic reactions were carried out under non-inert conditions. The following compounds were synthesized according to literature protocols: hydroxycarbamate 1a and 1-hydroxy-3-phenylurea 3f,^11,29^3a, 3b, 3c,^30^3e,^31^3h,^32^*E*,*E*-18,^33^*E*,*Z*-18.^24^ Solvents were dried with the help of a solvent drying system MB-SPS-800 from M. Braun. Silica gel from Merck (Millipore 60, 40–60 μm, 240–400 mesh) was used for column chromatography and silica pad filtrations. Reactions were monitored *via* thin layer chromatography (TLC) using precoated silica gel plates from Machery-Nagel (TLC Silica gel 60 F_254_). The spots were identified using irradiation with UV-light and a staining solution (basic potassium permanganate solution).^1^H- and ^13^C-NMR spectra were measured with a Bruker Avance NEO 400 at 20 °C. The chemical shifts are reported in ppm related to the signal of residual solvent of CDCl_3_ or DMSO-d_6_ (^1^H: (CDCl_3_) = 7.26 ppm, ^13^C: (CDCl_3_) = 77.2 ppm, ^1^H: (DMSO-d_6_) = 2.50 ppm, ^13^C: (DMSO-d_6_) = 39.5 ppm). Infrared spectra were recorded as thin film on a Bruker Alpha Eco ATR FTIR device. High resolution mass spectrometry was performed on an Agilent 6530 QTOF spectrometer.

### Representative protocol for the enzymatic intramolecular nitroso ene reaction: 3-hydroxy-4-(prop-1-en-2-yl)oxazolidine-2-one (2a)

To a 10 mM solution of *N*-hydroxycarbamate 1a in 7 mL phosphate buffer (pH 7.0, 100 mM) was added 70 U HRP and 70 U GOx. The reaction was initiated by adding d-glucose (50 mM) and incubated at room temperature. Full conversion was reached after 2 h and the reaction mixture was extracted 3× with EtOAc. The combined organic phases were dried over Na_2_SO_4_, filtered and the solvent was removed under reduced pressure. The crude was purified *via* silica pad filtration using EtOAc to yield 2a as colorless crystals (9.7 mg, 68 μmol, 143.14 g mol^−1^, 97%). Upscale reactions were scaled according to this procedure. ***R***_**f**_ = 0.31 (1 : 1 *n*-Hep/EtOAc). ^**1**^**H-NMR** (400 MHz, CDCl_3_): *δ* 8.29 (bs, 1H), 5.10 (dd, *J* = 8.4, 7.1 Hz, 2H), 4.49–4.30 (m, 2H), 4.08–3.97 (m, 1H), 1.81–1.76 (m, 3H). ^**13**^**C-NMR** (100 MHz, CDCl_3_): *δ* 160.9, 139.0, 117.0, 65.7, 64.8, 16.9. **FT-IR** (ATR) *ν* [cm^−1^] = 3236 (w), 2923 (w), 1743 (s), 1215 (m), 1095 (m). Spectral data are in agreement with literature precedent.^12^

### Representative protocol for the enzymatic intermolecular nitroso ene reaction: benzyl (2,3-dimethylbut-3-en-2-yl)(hydroxy)carbamate (4a)

To a solution of benzyl *N*-hydroxycarbamate 3a in 7 mL phosphate buffer (pH 7.0, 100 mM) containing 20 vol% *n*-heptane was added 100 mg Brij 35, 70 U HRP, 70 U GOx and 5.0 eq. of tetramethylethylene (5). The reaction was initiated by adding d-glucose (50 mM) and incubated at room temperature. Full conversion was observed after 45 min. 8 mL MeCN was added, and the reaction mixture was extracted 4× with EtOAc. The combined organic phases were dried over Na_2_SO_4_, filtered and the solvent was removed under reduced pressure. The crude was purified *via* column chromatography on silica gel (1 : 1 *n*-Hep/EtOAc) to yield 4a (16.2 mg, 65.0 μmol, 249.31 g mol^−1^, 93%). ***R***_**f**_ = 0.68 (1 : 1 *n*-Hep/EtOAc). ^**1**^**H-NMR** (400 MHz, CDCl_3_): *δ* [ppm] = 7.39–7.29 (m, 5H), 6.18 (bs, 1H), 5.18 (s, 2 h), 4.85 (s, 1H), 4.79–4.76 (m, 1 H), 1.75 (s, 3H), 1.48 (s, 6H). ^**13**^**C-NMR** (100 MHz, CDCl_3_): *δ* [ppm] = 158.2, 149.6, 135.9, 128.6, 128.4, 128.3, 109.6, 68.2, 66.1, 25.5, 19.2. **FT-IR** (ATR) *ν* [cm^−1^] = 3308 (vw), 2987 (vw), 2924 (vw), 1682 (m), 1454 (w), 1396 (w), 1327 (m), 1284 (w), 1164 (w), 1100 (m). Spectral data are in agreement with literature precedent.^12^

### Representative protocol for the enzymatic intermolecular nitroso Diels–Alder reaction: benzyl 2-oxa-3-azabicyclo[2.2.2]oct-5-ene-3-carboxylate (11a)

To a 10 mM solution of benzyl *N*-hydroxycarbamate 3a in 7 mL phosphate buffer (pH 7.0, 100 mM) containing 20 vol% EtOAc was added 70 U HRP, 14 U GOx and 3.0 eq. of cyclohexa-1,3-diene (10). The reaction was initiated by adding d-glucose (50 mM) and incubated at room temperature. Full conversion was reached after 2 h and the reaction mixture was extracted 3× with EtOAc. The combined organic phases were dried over Na_2_SO_4_, filtered and the solvent was removed under reduced pressure. The crude was purified *via* column chromatography on silica gel (8 : 1 *n*-Hep/EtOAc) to yield 11a (14.8 mg, 60.3 μmol, 245.28 g mol^−1^, 88%). ***R***_**f**_ = 0.68 (1 : 1 *n*-Hep/EtOAc). ^**1**^**H NMR** (400 MHz, CDCl_3_) *δ* [ppm] = 7.42–7.30 (m, 5H), 6.64–6.52 (m, 2H), 5.19 (dd, *J* = 29.2, 12.3 Hz, 2H), 4.85 (td, *J* = 5.4, 2.8 Hz, 1H), 4.79 (ddd, *J* = 5.5, 3.8, 1.9 Hz, 1H), 2.29–2.19 (m, 1H), 2.15 (ddt, *J* = 12.4, 9.0, 3.1 Hz, 1H), 1.52 (tt, *J* = 12.2, 2.9 Hz, 1H), 1.45–1.36 (m, 1H). ^**13**^**C NMR** (100 MHz, CDCl_3_) *δ* [ppm] = 158.2, 136.0, 132.0, 131.7, 128.5, 128.2, 128.1, 71.1, 67.7, 50.2, 23.5, 20.6. **FT-IR** (ATR): *ν* [cm^−1^] = 3062 (vw), 2983 (vw), 1736 (m), 1669 (m), 1260 (m), 1071 (s). Spectral data are in agreement with literature precedent.^25^

## Author contributions

Christina Jäger: Conceptualization (supporting); investigation – experimental (lead); writing – original draft (lead); validation analytical interpretation (lead). Bernhard J. Gregori: Investigation – experimental (supporting). Juhana A. S. Aho: Investigation – experimental (supporting). Marleen Hallamaa: Investigation – experimental (supporting). Jan Deska: Conceptualization (lead); writing – review and editing (lead); supervision (lead).

## Conflicts of interest

There are no conflicts to declare.

## Supplementary Material

GC-025-D2GC04827B-s001
