# Supplementary material for: Peroxidase-induced C–N bond formation via nitroso ene and Diels–Alder reactions
Source: Green Chem. 2023 Mar 21;25(8):3166–74. doi: 10.1039/d2gc04827b (PMC10124104; doi:10.1039/d2gc04827b)
Supplement: GC-025-D2GC04827B-s001 [file GC-025-D2GC04827B-s001.pdf]

Electronic Supplementary Material (ESI) for Green Chemistry.
This journal is © The Royal Society of Chemistry 2023

Peroxidase-induced C-N Bond Formation via Nitroso Ene and Diels-Alder Reactions

Christina Jäger, Bernhard J. Gregori, Juhana A. S. Aho, Marleen Hallamaa, Jan Deska

Department of Chemistry, University of Helsinki
A.I. Virtasen aukio 1, 00560 Helsinki, Finland
Email: jan.deska@helsinki.fi, Web: www.deskalab.com

Table of contents

General methods & materials 2

Synthesis of *E*,*E*-**18** and *E*,*Z*-**18** 3

Nitroso ene reactions (**4a**-**e**) 4

Solvent/additive screening 4

Enzymatic synthesis & analytical data 4

Nitroso Diels Alder reactions 6

Enzyme screening 6

GOx loading 6

Stability test (**3a**) 7

Synthesis of cis/trans-**19** references 7

Enzymatic synthesis & analytical data 8

HPLC-traces cis/trans-**19** products 13

E-factor calculations 14

NMR-spectra 16

Supplementary references 39

General methods & materials

Commercially available reagents were used without further purification. glucose oxidase (lypophilized powder, type II, 158200 U/g, *Aspergillus niger*) and horseradish peroxidase (lypophilized powder, beige, 173 U/mg) were obtained from Sigma Aldrich. All reactions were carried out under argon atmosphere and performed with dry solvents if not stated differently. All enzymatic reactions were carried out under non-inert conditions. The following compounds were synthesized according to literature protocols: hydroxycarbamate 1a and 1-hydroxy-3-phenylurea **3f**,[11,24] **3a**, **3b**, **3c**,[25] **3e** ,[26] **3h**,[27] *E*,*E*-**18**,[28], *E*,*Z*-**18**.[22] Solvents were dried with the help of a solvent drying system MB-SPS-800 from M. Braun. Silica gel from Merck (Millipore 60, 40-60 µm, 240-400 mesh) was used for column chromatography and silica pad filtrations. Reactions were monitored via thin layer chromatography (TLC) using precoated silica gel plates from Machery-Nagel (TLC Silica gel 60 F_{254}). The spots were identified using irradiation with UV-light and a staining solution (basic potassium permanganate solution).^{1}H- and ^{13}C-NMR spectra were measured with a Bruker Avance NEO 400 at 20 °C. The chemical shifts are reported in ppm related to the signal of residual solvent of $CDCl_3$ or DMSO-d_6 (^{1}H: ($CDCl_3$) = 7.26 ppm, ^{13}C: ($CDCl_3$) = 77.2 ppm, ^{1}H: (DMSO-d_6) = 2.50 ppm, ^{13}C: (DMSO-d_6) = 39.5 ppm). Infrared spectra were recorded as thin film on a Bruker Alpha Eco ATR FTIR device. High resolution mass spectrometry was performed on an Agilent 6530 QTOF spectrometer.

Synthesis of ***E,E*-18 and *E,Z*-18**

(2*E*,4*E*)-6-((tert-butyldimethylsilyl)oxy)hexa-2,4-dien-1-yl hydroxycarbamate (*E,E*-**18**)

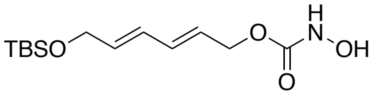

E,E-**18**

To a 0.2 M solution of (2*E*,4*E*)-6-((*tert*-butyldimethylsilyl)-oxy)hexa-2,4-dien-1-ol (0.860 g, 3.77 mmol, 1.0 eq.) in MeCN was added CDI (0.916 g, 5.66 mmol, 1.5 eq.). After full conversion (followed by TLC), imidazole (1.026 g, 15.06 mmol, 4.0 eq.) and hydroxylamine hydrochloride (1.309 g, 18.83 mmol, 5.0 eq.) were added. After observing full conversion of the intermediate, the solvent was removed under reduced pressure. The resulted solid was dissolved in EtOAc and 1 M HCl solution. The phases were separated, and the aqueous phase was washed 3x with EtOAc. The combined organic phases were dried over Na_2SO_4, filtered and the solvent was removed under reduced pressure. The crude was purified via column chromatography on silica gel (5:1 → 3:1 *n*-Hep/EtOAc) to yield *E,E*-**18** as an oil (595 mg, 2.07 mmol, 287.43 g/mol, 55 %). $\mathbf{R_f}$ = 0.11 (2:1 *n*-Hep/EtOAc). **^{1}H-NMR** (400 MHz, $CDCl_3$) δ [ppm] = 7.23 (s, 1H), 6.58 (s, 1H), 6.35 – 6.19 (m, 2H), 5.82 (dt, *J* = 14.9, 4.8 Hz, 1H), 5.72 (dt, *J* = 14.1, 6.9 Hz, 1H), 4.67 (d, *J* = 6.6 Hz, 2H), 4.22 (d, *J* = 4.8 Hz, 2H), 0.90 (s, 9H), 0.07 (s, 6H). **^{13}C-NMR** (100 MHz, $CDCl_3$) δ [ppm] = 159.1, 135.0, 134.7, 128.3, 125.4, 66.6, 63.3, 26.1, 18.5, -5.1. **FT-IR** (ATR) ν [cm^{-1}] = 3291 (vw), 2929 (vw), 1716 (m), 1458 (w), 1251 (m), 1097(s). **ESI-HRMS**: [$C_{13}H_{25}NO_4Si$, M+Na^+] calculated: 310.1445; found: 310.1445.

(2*E*,4*Z*)-6-((tert-butyldimethylsilyl)oxy)hexa-2,4-dien-1-yl hydroxycarba-mate (*E,Z*-**18**)

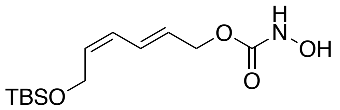

E,Z-**18**

To a 0.2 M solution of (2*E*,4*Z*)-6-((tert-butyldimethylsilyl)oxy)hexa-2,4-dien-1-ol (305.2 mg, 1.34 mmol, 1.0 eq.) in MeCN was added CDI (325 mg, 2.00 mmol, 1.5 eq.). After full conversion (followed by TLC), imidazole (364 mg, 5.36 mmol, 4.0 eq.) and hydroxylamine hydrochloride (465 g, 6.7 mmol, 5.0 eq.) were added. After observing full conversion of the intermediate, the solvent was removed under reduced pressure. The resulted solid was dissolved in EtOAc and 1 M HCl solution. The phases were separated, and the aqueous phase was washed 3x with EtOAc. The combined organic phases were dried over Na_2SO_4, filtered and the solvent was removed under reduced pressure. The crude was purified via column chromatography on silica gel (5:1 → 3:1 *n*-Hep/EtOAc) to yield *E,Z*-**18** as an oil (267 mg, 0.93 mmol, 287.43 g/mol, 69 %). $\mathbf{R_f}$ = 0.13 (2:1 *n*-Hep/EtOAc). **^{1}H-NMR** (400 MHz, $CDCl_3$) δ [ppm] = 7.06 – 7.01 (m, 2H), 6.56 (dd, J = 15.6, 10.1 Hz, 1H), 5.99 (t, J = 11.2 Hz, 1H), 5.74 (dt, J = 15.0, 6.6 Hz, 1H), 5.59 (dt, J = 11.8, 5.9 Hz, 1H), 4.67 (d, J = 6.5 Hz, 2H), 4.35 (dd, J = 6.4, 1.5 Hz, 2H), 0.90 (s, 9H), 0.08 (s, 6H). **^{13}C-NMR** (100 MHz, CDCl3) δ [ppm] = 159.1, 132.7, 129.7, 127.7, 127.3, 66.3, 59.7, 25.9, 18.3, -5.1**.** **FT-IR** (ATR) ν [cm^{-1}] = 3303 (vw), 2930 (vw), 1717 (m), 1473 (w), 1256 (m), 1094 (s). **ESI-HRMS**: [$C_{13}H_{25}NO_4Si$, M+Na^+] calculated: 310.1445; found: 310.1442.

Nitroso ene reactions

Solvent/additive screening

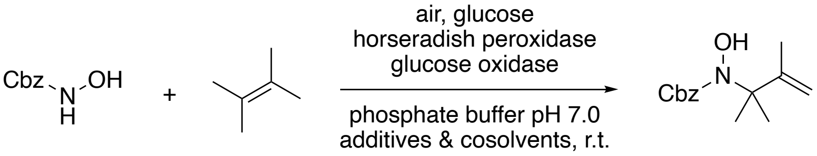

3a **5** **4a**

Table S1. Screening of 2,3-dimethylbut-2-ene loading in the nitroso-ene reaction of **3a** using GOx/HRP.

entry	**5** [equiv.]	solvent [% v/v]	additive	yield [%][a]
1	1.0	EtOAc (10)	–	30
2	1.5	EtOAc (10)	–	51
3	5.0	EtOAc (10)	–	55
4	–	**5** (10)	–	90
5	5.0	heptane (20)	Brij 35	93

[a] isolated yield.

Enzymatic synthesis & analytical data:

General procedure for the enzyme-mediated nitroso ene reactions

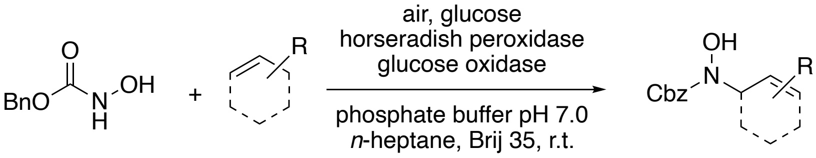

3a **4a-e**

To a 10 mM solution of benzyl hydroxycarbamate **3a** in 7 mL phosphate buffer (pH 7.0, 100 mM) containing 20 vol% *n*-heptane was added 100 mg Brij 35, 70 U HRP, 70 U GOx and 5.0 eq. of olefin. The reaction was initiated by adding D-glucose (50 mM) and incubated at room temperature. After full conversion (followed by TLC), 8 mL MeCN was added, and the reaction mixture was extracted 4x with EtOAc. The combined organic phases were dried over Na_2SO_4, filtered and the solvent was removed under reduced pressure. The crude was purified via column chromatography on silica gel.

Benzyl hydroxy(3-methylbut-3-en-2-yl)carbamate (**4b**)

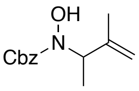

4b

According to the general procedure 2-methylbut-2-ene **6** was reacted. Full conversion was observed after 45 min. The crude was purified via column chromatography on silica gel (1:1 *n*-Hep/EtOAc) to yield **4b** (9.1 mg, 38.68 µmol, 235.28 g/mol, 55 %). **^{1}H-NMR** (400 MHz, $CDCl_3$): δ [ppm] = 7.39 – 7.29 (m, 5H), 6.09 (bs, 1H), 5.20 (s, 2H), 4.98 – 4.90 (d, 2H), 4.67 – 4.59 (q, 1H), 1.75 (s, 3H), 1.40 – 1.33 (d, 3H). **R_f** = 0.78 (1:1 *n*-Hep/EtOAc). **^{13}C-NMR** (100 MHz, $CDCl_3$): δ [ppm] = 157.2, 143.9, 136.1, 128.7, 128.5, 128.2, 112.8, 68.2, 58.5, 20.8, 15.2. **FT-IR** (ATR) ν [cm^{-1}] = 3274 (vw), 2977 (vw), 2943 (vw), 1696 (m), 1449 (w), 1410 (w), 1358 (w), 1303 (w), 1120 (w), 1075 (w). **ESI-HRMS**: [$C_{13}H_{17}NO_3$, M+Na^+] calculated: 258.1101; found: 258.1107.

Benzyl hydroxy(1-hydroxy-3-methylbut-3-en-2-yl)carbamate (**4c**)

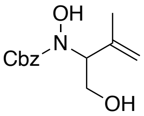

4c

According to the general procedure prenol **7** was reacted. Full conversion was observed after 60 min. The crude was purified via column chromatography on silica gel (1:1 *n*-Hep/EtOAc) to yield **4c** (13.5 mg, 53.72 µmol, 251.28 g/mol, 77 %). $\mathbf{R_f}$ = 0.14 (1:1 *n*-Hep/EtOAc). **^{1}H-NMR** (400 MHz, $CDCl_3$): δ [ppm] = 7.42 – 7.26 (m, 5H), 5.23 – 5.11 (q, 2H), 4.94 (s, 1H), 4.89 (s, 1H), 4.85 (s, 2H), 4.63 – 4.55 (dd, 1H), 3.96 – 3.86 (dd, 1H), 3.80 – 3.71 (dd, 1H), 1.75 (s, 3H). **^{13}C-NMR** (100 MHz, $CDCl_3$): δ [ppm] = 159.7, 142.7, 137.9, 129.5, 129.1, 128.9, 113.9, 68.6, 66.0, 60.6, 21.4. **FT-IR** (ATR) ν [cm^{-1}] = 3290 (vw), 2941 (vw), 1718 (w), 1456 (vw), 1403 (vw), 1299 (vw), 1255 (vw), 1104 (w). Spectral data are in agreement with literature reports.[1]

Benzyl cyclohex-2-en-1-yl(hydroxy)carbamate (**4d**)

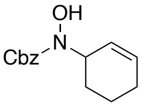

4d

According to the general procedure cyclohexene **8** was reacted as cosolvent (0.4 mL **8** + 1.0 mL *n*-heptane). Full conversion was observed after 45 min. The crude was purified via column chromatography on silica gel (1:1 *n*-Hep/EtOAc) to yield **4d** (5.7 mg, 23.05 µmol, 247.29 g/mol, 33 %). $\mathbf{R_f}$ = 0.76 (1:1 *n*-Hep/EtOAc). **^{1}H-NMR** (600 MHz, $CDCl_3$): δ [ppm] = 7.39 – 7.31 (m, 5H), 5.97 – 5.91 (m, 1H), 5.73 (bs, 1H), 5.59 – 5.53 (dd, 1H), 5.21 (s, 2H), 4.71 – 4.65 (m, 1H), 2.10 – 2.02 (m, 1H), 2.01 – 1.94 (m, 1H), 1.93 – 1.81 (m, 3H), 1.67 – 1.59 (m, 1H). **^{13}C-NMR** (150 MHz, $CDCl_3$): δ [ppm] = 157.4, 136.1, 132.3, 128.7, 128.5, 128.3, 126.4, 68.2, 55.7, 25.8, 24.5, 21.2. **FT-IR** (ATR) ν [cm^{-1}] = 3301 (vw), 3031 (vw), 2937 (vw), 2865 (vw), 1697 (w), 1455 (vw), 1411 (vw), 1344 (vw), 1295 (w), 1102 (w). Spectral data are in agreement with literature reports.[1]

Benzyl hydroxy(2-methylcyclohex-2-en-1-yl)carbamate *twix*-**4e** and benzyl hydroxy(2-methylenecyclohexyl)carbamate *twin*-**4e**

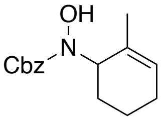

twix-**4e**

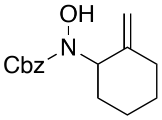

twin-**4e**

According to the general procedure 1-methylcyclohex-1-ene **9** was reacted as cosolvent (0.4 mL **9** + 1.0 mL *n*-heptane). Full conversion was observed after 45 min. The crude was purified via column chromatography on silica gel (1:1 *n*-Hep/EtOAc) to yield *twix*-**4e** and *twin*-**4e** in a 6:1 ratio (4.8 mg, 38.68 µmol, 261.32 g/mol, 26 %). $\mathbf{R_f}$ = 0.71 (1:1 *n*-Hep/ EtOAc).Mixture of isomers: **^{1}H-NMR** (600 MHz, $CDCl_3$): δ [ppm] = 7.42 – 7.31 (m, 5H), 5.76 (bs, 1H), 5.68 (bs, 1H), 5.23 – 5.20 (m, 2H), 4.80 (s, 1H), 4.68 (s, 1H), 4.57 (bs, 2H), 4.53 – 4.47 (m, 2H), 2.46 – 2.35 (m, 2H), 2.09 – 2.00 (m, 1H), 1.99 – 1.78 (m, 4H), 1.62 (s, 3H), 1.60 – 1.53 (m, 1H). **^{13}C-NMR** (100 MHz, $CDCl_3$): δ [ppm] = 157.5, 156.5, 155.4, 145.6, 136.1, 135.0, 134.1, 131.8, 129.8, 129.2, 128.9, 128.8, 128.7, 128.5, 128.4, 128.3, 128.2, 127.9, 106.4, 71.8, 68.8, 68.7, 68.2, 68.1, 64.4, 61.6, 58.2, 36.2, 35.2, 30.4, 29.8, 27.1, 26.9, 26.2, 25.6, 25.1, 21.2, 20.8, 20.3.**FT-IR** (ATR) ν [cm^{-1}] = 3280 (vw), 2935 (vw), 1696 (m), 1455 (w), 1408 (w), 1292 (w), 1214 (w), 1100 (w). Spectral data are in agreement with literature reports.[1]

Nitroso Diels Alder reactions

Enzyme screening

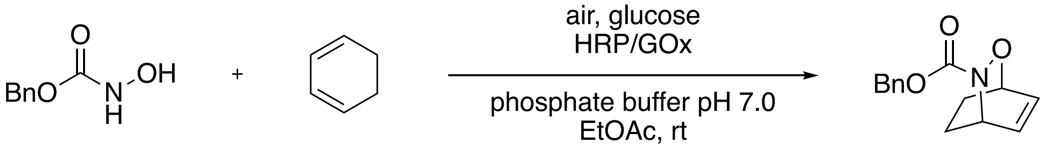

3a **10** **11a**

To a 10 mM solution of benzyl hydroxycarbamate **3a** in 7 mL phosphate buffer (pH 7.0, 100 mM) containing 10 vol% EtOAc was added 100 U laccase or the HRP/GOx couple (70 U each). 1.5 eq. of 1,3-cyclohexadiene **10** was added and the reaction was initiated by adding D-glucose (50 mM). After full conversion (followed by TLC), the mixture was extracted 3x with DCM. The organic phase was filtered through a short silica plug and the solvent was removed under reduced pressure. The yield of **11a** was determined by quantitative ^{1}H-NMR with dimethylsulfone as internal standard.

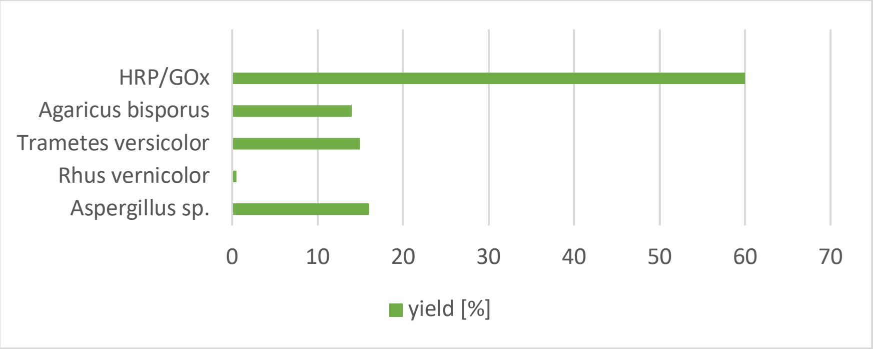

Figure S1. Screening of different laccases in comparison to HRP/GOx for the intermolecular nitroso-Diels-Alder reaction of **3a** and **10**.

GOx-loading

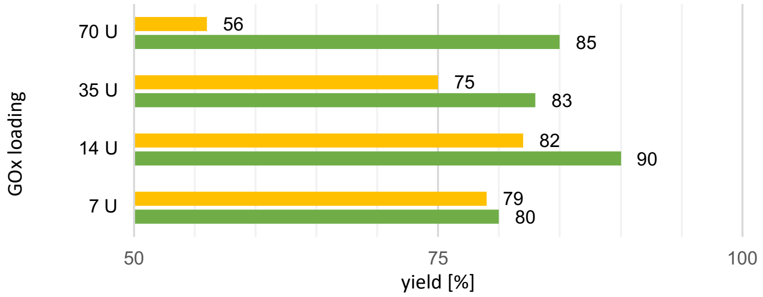

Figure S2. Influence of GOx loading in the intermolecular nitroso Diels-Alder reaction. Reaction conditions: To a solution of 10 mM of **3a** in 7 mL phosphate buffer (pH 7.0, 100 mM) containing 20 vol% cosolvent was added 70 U HRP, 70 U GOx, D-glucose (50 mM) and 3.0 eq. of **10**. The reaction was incubated at room temperature. ■ *n*-heptane, ■ EtOAc.

Stability test

Table S2. Stability of **3a** under the reaction conditions.

#	conditions	full conversion after x h	yield **11a** [%]
1	no enzymes	-	-
2	no GOx	-	-
3	no HRP	-	-
4	no diene[a]	20	0
5	-	2	47

Reaction conditions: 70 U HRP, 14 U GOx and 5 equiv. D-Glucose were dissolved in 6 mL phosphate buffer (0.1 M, pH 7) and a solution of 0.14 mmol **11a** in 1 mL EtOAc was added. After 20 h, the mixture was extracted three times with 20 mL EtOAc. The organic phase was filtered through a short silica plug and the solvent was removed under reduced pressure. [a] Reaction conditions: 0.28 mmol **11a**, 5.0 eq. D-glucose, 140 U HRP, 28 U GOx, 4 mL phosphate buffer, 2 mL EtOAc.

Synthesis of *cis/trans*-19 references

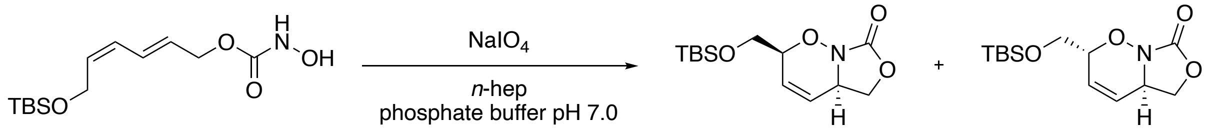

E,Z-**18** *cis*-**19** *trans*-**19**

(2*E*,4*Z*)-6-((tert-butyldimethylsilyl)oxy)hexa-2,4-dien-1-yl hydroxycarbamate *E,Z*-**18** (21 mg, 0.07 mmol, 1 eq.) was dissolved in 1 mL *n*-heptane. 1 mL phosphate buffer was added, and the mixture was cooled to 0 °C followed by the addition of sodium periodate (22.6 mg, 0.106 mmol, 1.45 eq.). The mixture was stirred vigorously at 0 °C for 10 min. The reaction was allowed to warm to room temperature and stirred for 1 h. The phases were separated, and the aqueous phase was extracted 4x with EtOAc. The combined organic phases were washed with brine, dried over Na_2SO_4 and the solvent was removed under reduced pressure. The crude was purified via column chromatography (5:1 *n*-Pen/Et_2O, **R_f** = 0.56 (*trans*), 0.58 (*cis*) (1:1 *n*-Hep/EtOAc)) to separate *trans*- and *cis*-isomers of *cis*/*trans*-**19** (18.5 mg, 89 %, *trans*+*cis* combined, 76:24 ratio) as white solid.

trans-**19**: **R_f** = 0.56 (1:1 *n*-Hep/EtOAc). **^{1}H-NMR** (400 MHz, $CDCl_3$) δ [ppm] = 6.13 (dt, *J* = 10.7, 1.3 Hz, 1H), 5.89 (ddd, *J* = 10.5, 2.4, 1.0 Hz, 1H), 4.80 – 4.75 (m, 1H), 4.48 – 4.41 (m, 2H), 4.14 (dt, *J* = 9.8, 4.2 Hz, 1H), 3.82 (dd, *J* = 10.9, 4.4 Hz, 1H), 3.69 (dd, *J* = 10.9, 5.3 Hz, 1H), 0.88 (s, 9H), 0.07 (s, 6H). **^{13}C-NMR** (100 MHz, $CDCl_3$) δ [ppm] = 157.6, 129.8, 123.9, 74.5, 66.7, 63.4, 52.6, 26.0, 18.3, -5.2.

cis-**19**: **R_f** = 0.58 (1:1 *n*-Hep/EtOAc). **^{1}H-NMR** (400 MHz, $CDCl_3$) δ [ppm] = (ddd, *J* = 10.7, 3.4, 1.7 Hz, 1H), 5.92 (dt, *J* = 10.8, 1.7 Hz, 1H), 4.53 – 4.39 (m, 3H), 4.09 (dd, *J* = 8.0, 2.4 Hz, 1H) 4.02 (dd, *J* = 10.1, 5.4 Hz, 1H), 3.80 (dd, *J* = 10.3, 8.3 Hz, 1H), 0.89 (s, 9H), 0.08 (d, *J* = 6.0 Hz,

6H). **^{13}C-NMR** (100 MHz, $CDCl_3$) δ [ppm] = 156.9, 128.1, 124.2, 79.4, 65.7, 64.8, 50.9, 25.9, 18.3, -5.4.

FT-IR (ATR) ν [cm^{-1}] = 2927 (vw), 1792 (s), 1541 (w), 1082 (s)
HPLC: 98:3 *n*-hexane/IPA; Chiralpak AS. t_R [min] = 18.4 & 27.5 (*cis*-**19**), 20.8 & 31.8 (*trans*-**19**)

Enzymatic synthesis & analytical data

General procedure for the enzyme mediated nitroso Diels Alder reactions

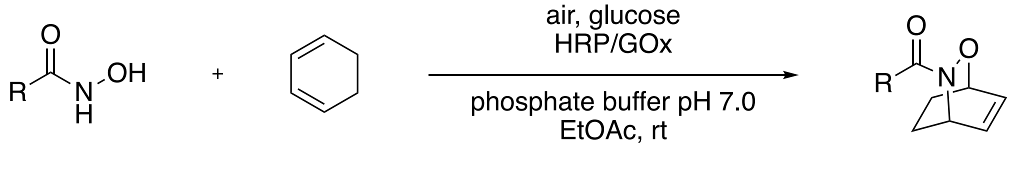

3a-g **10** **11a-g**

To a 10 mM solution of hydroxycarbamate in 7 mL phosphate buffer (pH 7.0, 100 mM) containing 20 vol% EtOAc was added 70 U HRP, 14 U GOx and 3.0 eq. of diene. The reaction was initiated by adding D-glucose (50 mM) and incubated at room temperature. After full conversion (followed by TLC), the reaction mixture was extracted 3x with EtOAc. The combined organic phases were dried over Na_2SO_4, filtered and the solvent was removed under reduced pressure. The crude was purified via column chromatography on silica gel.

1-(2-oxa-3-azabicyclo[2.2.2]oct-5-en-3-yl)-2-phenylethan-1-one (**11b**)

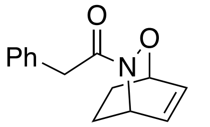

11b

According to the general procedure *N*-hydroxy-2-phenylacetamide **3b** was reacted. Full conversion was observed after 2 h. The crude was purified via column chromatography on silica gel (3:1 *n*-Hep/EtOAc) to yield **11b** (8.1 mg, 35.33 µmol, 229.28 g/mol, 50 %). **R_f** = 0.40 (1:1 *n*-Hep/EtOAc). **^{1}H-NMR** (400 MHz, $CDCl_3$) δ [ppm] = 7.40 – 7.17 (m, 5H), 6.65 – 6.56 (m, 1H), 6.47 – 6.39 (m, 1H), 5.26 (d, *J* = 4.2 Hz, 1H), 4.74 (d, *J* = 3.6 Hz, 1H), 3.61 (q, *J* = 14.5 Hz, 2H), 2.18 – 1.98 (m, 2H), 1.52 – 1.39 (m, 2H).**^{13}C-NMR** (100 MHz, $CDCl_3$) δ [ppm] = 170.5, 134.8, 133.0, 131.3, 129.5, 129.4, 128.3, 126.5, 71.9, 46.6, 40.1, 23.4, 21.0.**FT-IR** (ATR): ν [cm^{-1}] = 3061 (vw), 2937 (vw), 1644 (m), 1495 (w), 1363 (w), 1164 (w). Spectral data are in agreement with literature reports.[2]

(2-oxa-3-azabicyclo[2.2.2]oct-5-en-3-yl)(phenyl)methanone (**11c**)

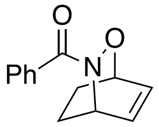

11c

According to the general procedure *N*-hydroxybenzamide **3c** was reacted. Full conversion was observed after 2 h. The crude was purified via column chromatography on silica gel (5:1 *n*-

Hep/EtOAc) to yield **11c** (12.7 mg, 59.0 µmol, 215.25 g/mol, 85 %). R_f = 0.38 (1:1 *n*-Hep/EtOAc). Rotameric mixture: **^{1}H-NMR** (400 MHz, $CDCl_3$) δ [ppm] = 7.64 (s, 2H), 7.45 – 7.36 (m, 3H), 6.65 (s, 1H), 6.54 (s, 1H), 5.35 (s, 1H), 4.80 (s, 1H, H-2/H-5), 2.23 – 2.19 (m, 2H), 1.57 – 1.48 (m, 2H). **^{13}C-NMR** (100 MHz, $CDCl_3$) δ [ppm] = 168.9, 134.4, 133.1, 131.8, 130.8, 128.5, 128.0, 72.0, 47.1, 23.6, 21.2. **FT-IR** (ATR): ν [cm^{-1}] = 3059 (vw), 2936 (vw), 1631 (m), 1610 (m), 1448 (w), 1268 (w), 921 (m). Spectral data are in agreement with literature reports.[2]

1-(2-oxa-3-azabicyclo[2.2.2]oct-5-en-3-yl)ethan-1-one (**11d**)

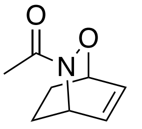

11d

According to the general procedure commercial *N*-hydroxyacetamide **3d** was reacted. The reaction was stopped after 3 h. The crude was purified via column chromatography on silica gel (DCM + 5 % MeOH) to yield **11d** (1.9 mg, 12.4 µmol, 153.18 g/mol, 18 %). R_f = 0.67 (95:5 DCM/MeOH). **^{1}H-NMR** (400 MHz, $CDCl_3$) δ [ppm] = 6.68 – 6.56 (m, 1H), 6.56 – 6.43 (m, 1H), 5.27 (d, *J* = 1.6 Hz, 1H), 4.74 (d, *J* = 5.0 Hz, 1H), 2.21 (td, *J* = 9.3, 3.9 Hz, 1H), 2.12 – 2.05 (m, 1H), 1.96 (s, 3H), 1.54 – 1.42 (m, 2H). **^{13}C-NMR** (100 MHz, $CDCl_3$) δ [ppm] = 170.4, 133.1, 131.3, 71.8, 46.2, 23.5, 21.1, 21.0. **FT-IR** (ATR): ν [cm^{-1}] = 3414 (vw), 2929 (m), 1649 (s), 1365 (m), 1047 (m), 951 (m). Spectral data are in agreement with literature reports.[2]

Allyl 2-oxa-3-azabicyclo[2.2.2]oct-5-ene-3-carboxylate (**11e**)

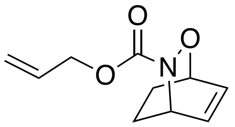

11e

According to the general procedure allyl hydroxycarbamate **3e** was reacted. Full conversion was observed after 1 h. The crude was purified via silica plug filtration (1:1 *n*-Hep/EtOAc) to yield **11e** (11.2 mg, 57.4 µmol, 195.22 g/mol, 82 %). R_f = 0.62 (1:1 *n*-Hep/EtOAc). **^{1}H-NMR** (400 MHz, $CDCl_3$) δ [ppm] =6.64 – 6.50 (m, 2H), 5.92 (ddt, *J* = 17.1, 10.5, 5.7 Hz, 1H), 5.26 (ddq, *J* = 29.3, 10.4, 1.4 Hz, 2H), 4.82 (td, *J* = 5.4, 2.8 Hz, 1H), 4.76 (qd, *J* = 3.5, 1.5 Hz, 1H), 4.68 – 4.55 (m, 2H), 2.26 – 2.08 (m, 2H), 1.51 (tt, *J* = 12.3, 2.8 Hz, 1H), 1.46 – 1.34 (m, 1H). **^{13}C-NMR** (100 MHz, $CDCl_3$) δ [ppm] = 157.9, 132.2, 132.0, 131.6, 118.3, 71.1, 66.7, 50.1, 23.5, 20.6. **FT-IR** (ATR): ν [cm^{-1}] = 2937 (vw), 1736 (w), 1699 (m), 1386 (w), 1260 (s), 1073 (m). **ESI-HRMS**: [$C_{10}H_{13}NO_3$, M+Na$^+$] calculated: 218.0788; found: 218.0788.

N-phenyl-2-oxa-3-azabicyclo[2.2.2]oct-5-ene-3-carboxamide (**11f**)

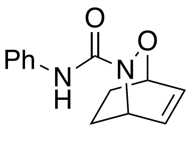

11f

According to the general procedure 1-hydroxy-3-phenylurea **3f** was reacted. Full conversion was observed after 1.5 h. The crude was purified via column chromatography on silica gel (8:1 *n*-Hep/EtOAc) to yield **11f** (15.9 mg, 69.1 µmol, 230.27 g/mol, 98 %). R_f = 0.57 (1:1 *n*-

Hep/EtOAc).^{1}**H-NMR** (400 MHz, $CDCl_3$) δ [ppm] = 7.65 (bs, 1H, -NH-), 7.46 (dt, *J* = 8.7, 1.6 Hz, 2H), 7.34 – 7.27 (m, 2H), 7.10 – 7.04 (m, 1H), 6.66 – 6.58 (m, 1H), 6.58 – 6.51 (m, 1H), 5.05 (ddd, *J* = 5.8, 4.0, 2.4 Hz, 1H), 4.82 (ddd, *J* = 5.5, 3.9, 1.6 Hz, 1H), 2.32 – 2.12 (m, 2H), 1.60 (ddd, *J* = 12.9, 7.7, 3.0 Hz, 1H), 1.45 – 1.38 (m, 1H). ^{13}**C-NMR** (100 MHz, $CDCl_3$) δ [ppm] = 159.4, 137.7, 132.7, 130.4, 128.9, 123.6, 119.3, 71.2, 50.0, 23.9, 20.0. **FT-IR** (ATR): ν [cm^{-1}] = 3393 (vw), 2935 (vw), 167,2 (m), 1518 (s), 1442 (s), 1213 (m). Spectral data are in agreement with literature reports.[2]

2-oxa-3-azabicyclo[2.2.2]oct-5-ene-3-carboxamide (**11g**)

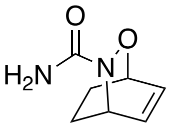

11g

According to the general procedure commercial hydroxyurea **3g** was reacted. The reaction was stopped after 3 h. The crude was purified via column chromatography on silica gel (DCM + 5 % MeOH) to yield **11g** (1.2 mg, 7.78 µmol, 154.17 g/mol, 11 %). $\mathbf{R_f}$ = 0.62 (95:5 DCM/MeOH). ^{1}**H-NMR** (400 MHz, $CDCl_3$) δ [ppm] = 6.61 – 6.54 (m, 1H), 6.54 – 6.47 (m, 1H), 5.20 (bs, 2H), 4.95 – 4.89 (m, 1H), 4.69 (qd, *J* = 3.5, 1.6 Hz, 1H), 2.23 – 2.07 (m, 2H), 1.52 (ddd, *J* = 12.8, 7.5, 2.8 Hz, 1H), 1.41 – 1.32 (m, 1H). ^{13}**C-NMR** (100 MHz, $CDCl_3$) δ [ppm] = 163.23, 132.39, 130.49, 70.86, 49.64, 23.75, 20.1. **FT-IR** (ATR): ν [cm^{-1}] = 3541 (vw), 3286 (vw), 2935 (vw), 1669 (s), 1578 (m), 1400 (m). Spectral data are in agreement with literature reports.[2]

Benzyl 4,5-dimethyl-3,6-dihydro-2H-1,2-oxazine-2-carboxylate (**13**)

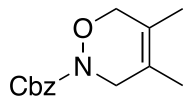

13

According to the general procedure benzyl hydroxycarbamate **3a** and 2,3-dimethylbuta-1,3-diene **12** were reacted. Full conversion was observed after 2 h. The crude was purified via column chromatography on silica gel (10:1 *n*-Hep/EtOAc) to yield **13** (7.0 mg, 28.3 µmol, 247.29 g/mol, 41 %). $\mathbf{R_f}$ = 0.72 (1:1 *n*-Hep/EtOAc). ^{1}**H-NMR** (400 MHz, $CDCl_3$) δ [ppm] = 7.42 – 7.29 (m, 5H), 5.21 (s, 2H), 4.22 (d, *J* = 0.8 Hz, 2H), 3.97 (d, *J* = 0.9 Hz, 2H), 1.74 – 1.62 (m, 3H), 1.64 – 1.53 (m, 3H). ^{13}**C-NMR** (100 MHz, $CDCl_3$) δ [ppm] = 155.5, 136.1, 128.6, 128.2, 128.2, 123.1, 121.8, 71.7, 67.7, 48.4, 15.2, 13.8. **FT-IR** (ATR): ν [cm^{-1}] = 2971 (vw), 2849 (vw), 1708 (m), 1407 (w), 1216 (s), 1086 (m). Spectral data are in agreement with literature reports.[2]

Benzylmethyl-3,6-dihydro-2H-1,2-oxazine-2-carboxylate (*prox*-**15**) and (*dist*-**15**)

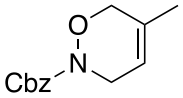

prox-**15**

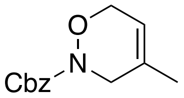

dist-**15**

According to the general procedure benzyl hydroxycarbamate **3a** and isoprene **14** were reacted. Full conversion was observed after 2.5 h. The crude was purified via column chromatography on silica gel (20:1 *n*-Hep/EtOAc) to yield *dist*-**15** and *prox*-**15** in a 2:1 ratio (4.1 mg, 17.58 µmol, 233.27 g/mol, 26 %). $\mathbf{R_f}$ = 0.67 (prox), 0.63 (dist)(1:1 *n*-Hep/EtOAc).Major

Isomer: **^{1}H-NMR** (400 MHz, $CDCl_3$) δ [ppm] = 7.45 – 7.31 (m, 5H), 5.55 (dtd, *J* = 5.0, 3.1, 1.6 Hz, 1H), 5.33 – 5.18 (m, 2H), 4.41 (dt, *J* = 4.5, 2.2 Hz, 2H), 4.04 (d, *J* = 0.7 Hz, 2H), 1.79 – 1.73 (m, 3H). **^{13}C-NMR** (100 MHz, $CDCl_3$) δ [ppm] = 155.5, 136.1, 131.6, 130.1, 128.6, 128.3, 128.2, 118.0, 116.3, 71.7, 67.7, 48.5, 19.7. Minor Isomer (selected signals): **^{1}H-NMR** (400 MHz, $CDCl_3$) δ [ppm] = 4.30 (s, 2H), 4.13 (dt, *J* = 5.3, 2.2 Hz, 2H) , 1.73 – 1.65 (m, 3H). **^{13}C-NMR** (100 MHz, $CDCl_3$) δ [ppm] = 155.7, 71.6, 68.2, 44.8, 18.3. **FT-IR** (ATR): ν [cm^{-1}] = 2917 (vw), 2850 (vw), 1704 (m), 1405 (w), 1215 (s), 1097 (m). Spectral data are in agreement with literature reports.[2]

Benzyl(hydroxymethyl)methyl-3,6-dihydro-2H-1,2-oxazine-2-carboxylat (*prox*-**17**) and (*dist*-**17**)

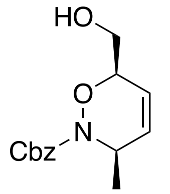

prox-**17**

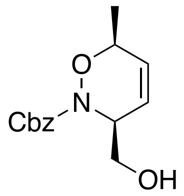

dist-**17**

According to the general procedure benzyl hydroxycarbamate **3a** and sorbic alcohol *E,E*-**16** were reacted. Full conversion was observed after 2 h. The crude was purified via column chromatography on silica gel (6:1 → 3:1 *n*-Hep/EtOAc) to yield *dist*-**17** and *prox*-**17** in a 3:7 ratio (15.1 mg, 57.35 µmol, 263.29 g/mol, 83 %). **R_f** = 0.36 (prox), 0.31 (dist)(1:1 *n*-Hep/EtOAc). Major Isomer: **^{1}H-NMR** (400 MHz, $CDCl_3$) δ [ppm] = 7.44 – 7.31 (m, 5H), 5.95 (ddd, *J* = 10.3, 4.4, 2.2 Hz, 1H), 5.72 (dt, *J* = 10.3, 1.6 Hz, 1H), 5.24 (q, *J* = 12.3 Hz, 2H), 4.74 – 4.66 (m, 1H), 4.62 – 4.50 (m, 1H), 3.76 (dtd, *J* = 32.5, 12.0, 6.2 Hz, 2H), 2.28 (bs, 1H), 1.35 (d, *J* = 6.7 Hz, 3H). **^{13}C-NMR** (100 MHz, $CDCl_3$) δ [ppm] = 154.9, 136.0, 131.4, 130.2, 128.6, 128.3, 128.1, 123.8, 122.4, 78.8, 67.6, 63.6, 50.6, 18.3. Minor Isomer (selected signals): **^{1}H-NMR** (400 MHz, $CDCl_3$) δ [ppm] = 5.86 (d, *J* = 10.4 Hz, 1H), 5.81 (ddd, *J* = 10.4, 3.9, 1.8 Hz, 1H), 1.86 (bs, 1H), 1.29 (d, *J* = 6.7 Hz, 2H). **^{13}C-NMR** (100 MHz, $CDCl_3$) δ [ppm] = 67.8, 63.3, 18.8. **FT-IR** (ATR): ν [cm^{-1}] = 3414 (vw), 2931 (vw), 2873 (vw), 1699 (s), 1410 (m), 1305 (s), 1112 (s), 1068 (s). Spectral data are in agreement with literature reports.[3]

2-(((tert-Butyldimethylsilyl)oxy)methyl)-4a,5-dihydro-2H,7H-oxazolo[3,4-b][1,2]oxazin-7-one (*cis*-**19**)

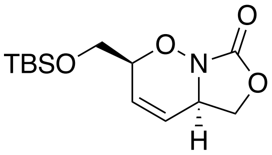

cis-**19**

Ten parallel reactions were performed. To a 10 mM solution of hydroxycarbamate *E,E*-**18** in 7 mL phosphate buffer (pH 7.0, 100 mM) containing 2 mL *n*-heptane, was added 70 U HRP and 70 U GOx. The reaction was initiated by adding D-glucose (50 mM) and incubated at 25 °C. Full conversion was reached after 1.5 h and all ten reactions were combined for the work-up. The reaction mixture was extracted 3x with EtOAc. The combined organic phases were dried over Na_2SO_4, filtered and the solvent was removed under reduced pressure. The crude was purified via column chromatography on silica gel (5:1 *n*-Pen/Et_2O) to yield *cis*-**19** as white solid

as single isomer (143.4 mg, 0.5 mmol, 285.42 g/mol, 72 %). **^{1}H-NMR** (400 MHz, $CDCl_3$) δ [ppm] = (ddd, *J* = 10.7, 3.4, 1.7 Hz, 1H), 5.92 (dt, *J* = 10.8, 1.7 Hz, 1H), 4.53 – 4.39 (m, 3H), 4.09 (dd, *J* = 8.0, 2.4 Hz, 1H) 4.02 (dd, *J* = 10.1, 5.4 Hz, 1H), 3.80 (dd, *J* = 10.3, 8.3 Hz, 1H), 0.89 (s, 9H), 0.08 (d, *J* = 6.0 Hz, 6H). **^{13}C-NMR** (100 MHz, $CDCl_3$) δ [ppm] = 156.9, 128.1, 124.2, 79.4, 65.7, 64.8, 50.9, 25.9, 18.3, -5.4. **FT-IR** (ATR) ν [cm^{-1}] = 2930 (vw), 1792 (s), 1541 (w), 1088 (s). **Melting point**: 69.2 – 70.2 °C. **ESI-HRMS**: [$C_{13}H_{23}NO_4Si$, M+H$^+$] calculated: 286.1469; found: 286.1489.

2-(((tert-Butyldimethylsilyl)oxy)methyl)-4a,5-dihydro-2H,7H-oxazolo[3,4-b][1,2]oxazin-7-one (*trans*-**19**)

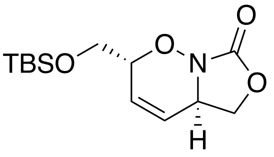

trans-**19**

Ten parallel reactions were performed. To a 10 mM solution of hydroxycarbamate *E,Z*-**18** in 18 mL phosphate buffer (pH 7.0, 100 mM) containing 5.1 mL *n*-heptane, was added 180 U HRP and 180 U GOx. The reaction was initiated by adding D-glucose (50 mM) and incubated at 25 °C. Full conversion was reached after 1.5 h and all ten reactions were combined for the work-up. The reaction mixture was extracted 3x with EtOAc. The combined organic phases were dried over Na_2SO_4, filtered and the solvent was removed under reduced pressure. The crude was purified via column chromatography on silica gel (5:1 *n*-Pen/Et_2O) to yield *trans*-**19** as white solid in a *cis*/*trans* ratio of 45:55 (22.5 mg, 78.8 µmol, 285.42 g/mol, 44 %).

HPLC-traces *cis*/*trans*-19 products

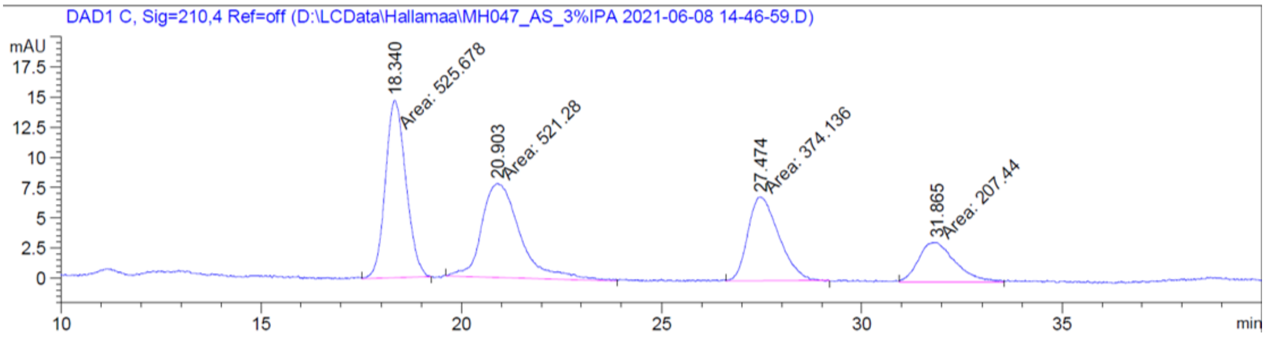

Figure S3. HPLC-chromatogram of *cis*/*trans*-**19**.

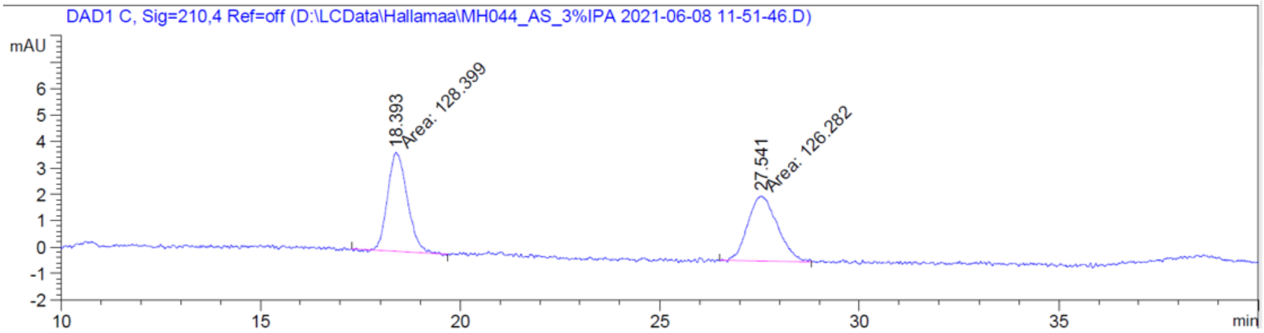

Figure S4. HPLC-chromatogram of *rac*-*cis*-**19**.

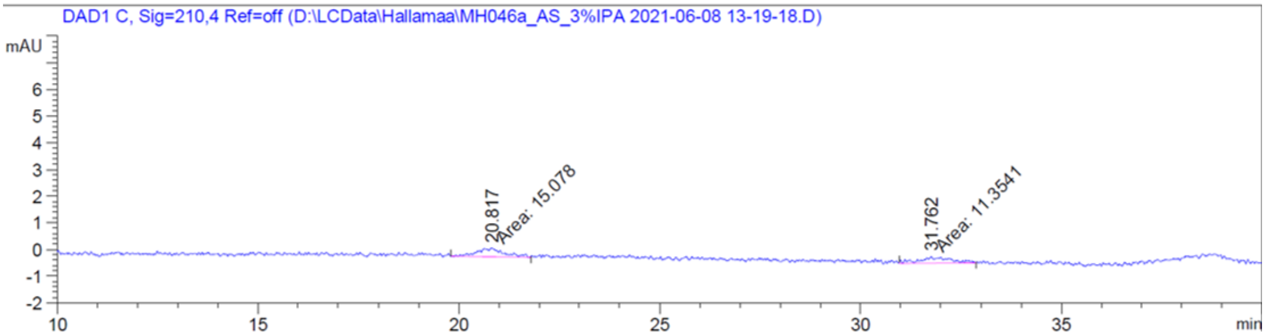

Figure S5. HPLC-chromatogram of *rac*-*trans*-**19**.

E-factor calculations

Oxidation with $PhI(OAc)_2$ [4]

DMF	2.15 g	sEF	37,35
DCM	63.24 g	**cEF**	**749**
$PhI(OAc)_2$	0.483 g		
thiosulfat	3.953 g		
H_2O	25 g		
m olefin	0.337 g		
m starting material	0.098 g		
m product	0.127 g		

Oxidation with $FeCl_3/H_2O_2$ [5]

isopropanol	4.68 g	sEF	6,40
$FeCl_3$	0.0054 g	**cEF**	**580**
H_2O_2	0.24 g		
aq. HCl	20 g		
m starting material	0.073 g		
m product	0.043 g		

Oxidation with CuCl/pyridine/air [1]

THF	17.8 g	sEF	32.0
CuCl	0.0034 g	**cEF**	**443**
pyridine	0.00066 g		
EDTA	2.93 g		
water	20 g		
m starting material	0.1 g		
m product	0.092 g		

Oxidation with HRP/GOx/Glc/air, unoptimized [6]

water	7	sEF	17.9
KH_2PO_4/K_2HPO_4	0.111 g	**cEF**	**732**
GOx	0.000442 g		
HRP	0.000405 g		
Glucose	0.063 g		
m starting material	0.0102 g		
m product	0.0098 g		

Oxidation with HRP/GOx/Glc/air, 25 mM

water	7	sEF	8.2
KH_2PO_4/K_2HPO_4	0.111 g	**cEF**	**329**
GOx	0.000442 g		
HRP	0.000405 g		
Glucose	0.063 g		
m starting material	0.0254 g		
m product	0.0218 g		

Oxidation with HRP/GOx/Glc/air, 50 mM

water	7	sEF	5.4
KH_2PO_4/K_2HPO_4	0.111 g	**cEF**	**205**
GOx	0.000442 g		
HRP	0.000405 g		
Glucose	0.063 g		
m starting material	0.0508 g		
m product	0.0351 g		

Oxidation with HRP/GOx/Glc/air, recycling study

water	7	sEF	3.7
KH_2PO_4/K_2HPO_4	0.111 g	**cEF**	**145**
GOx	0.000442 g		
HRP	0.000405 g		
Glucose	0.063 g		
m starting material	0.0508 g		
m product	0.0494 g		

NMR-Spectra

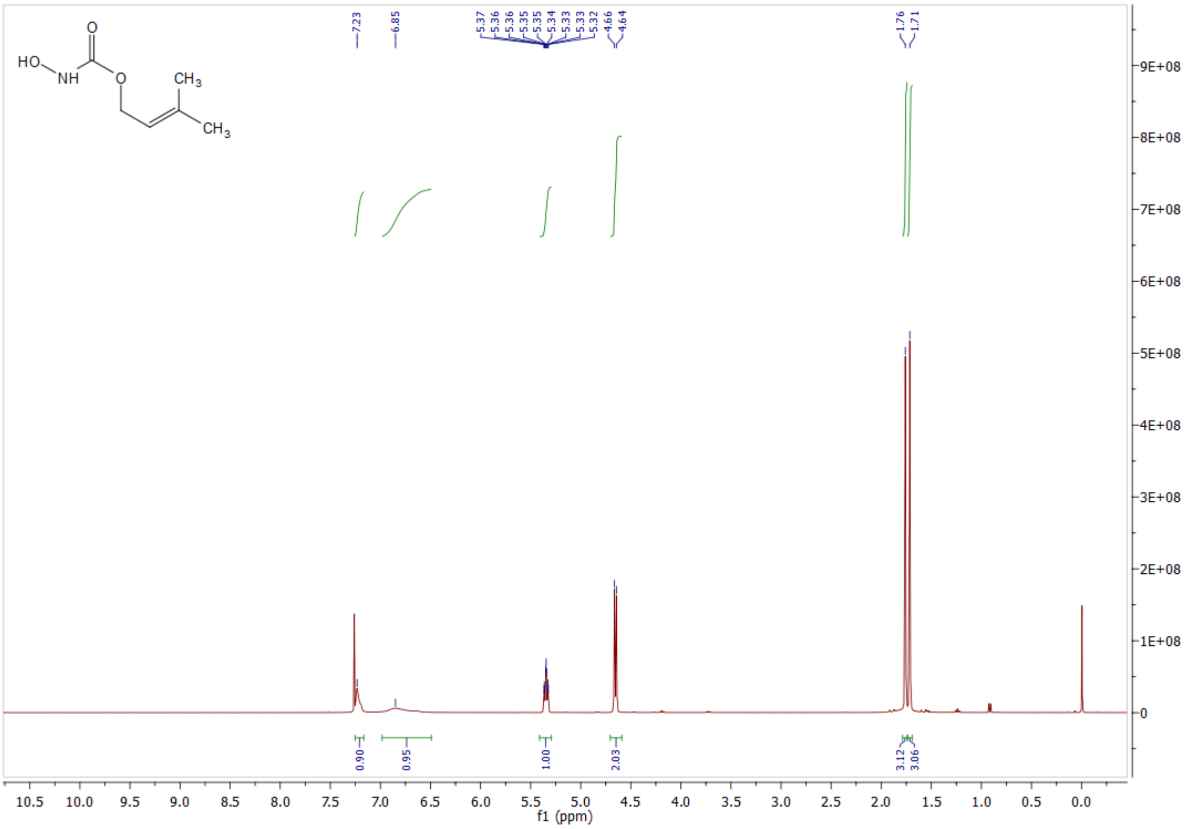

HO
NH
O
O
CH3
CH3
7.23
6.85
5.37
5.36
5.36
5.35
5.35
5.34
5.33
5.33
5.32
4.66
4.64
1.76
1.71
0.90
0.95
1.00
2.03
3.12
3.06
f1 (ppm)

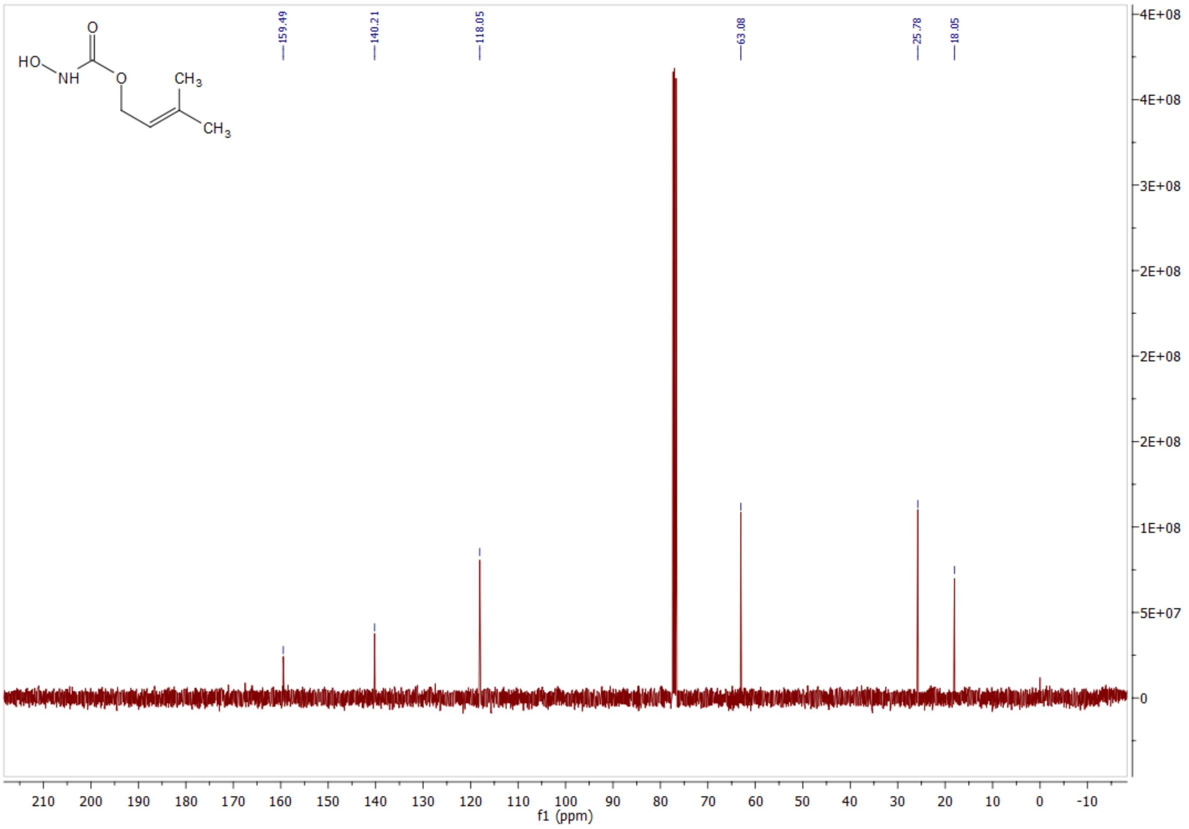

HO
NH
O
O
CH3
CH3
159.49
140.21
118.05
63.08
25.78
18.05
f1 (ppm)

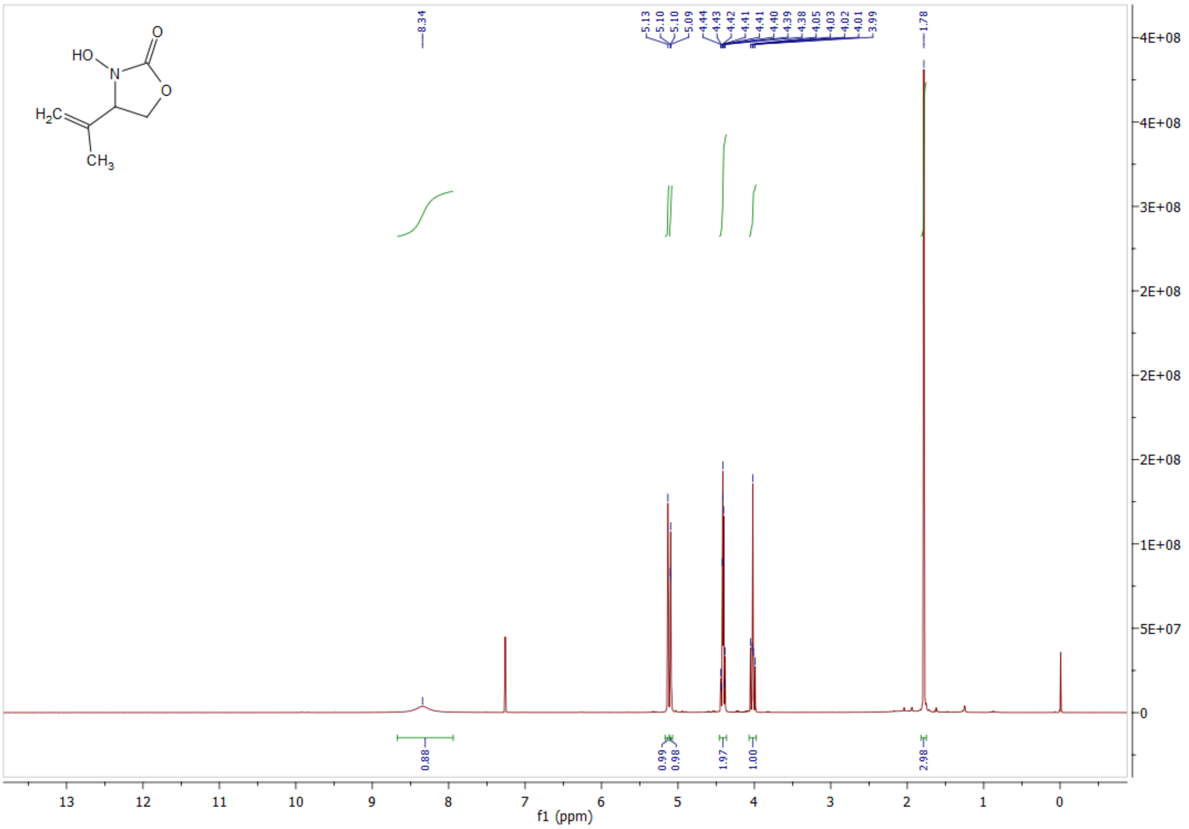
HO
N
O
O
H_2C
CH_3
8.34
5.13
5.10
5.10
5.09
4.44
4.43
4.42
4.41
4.41
4.40
4.39
4.38
4.05
4.03
4.02
4.01
3.99
1.78
0.88
0.99
0.98
1.97
1.00
2.98
f1 (ppm)

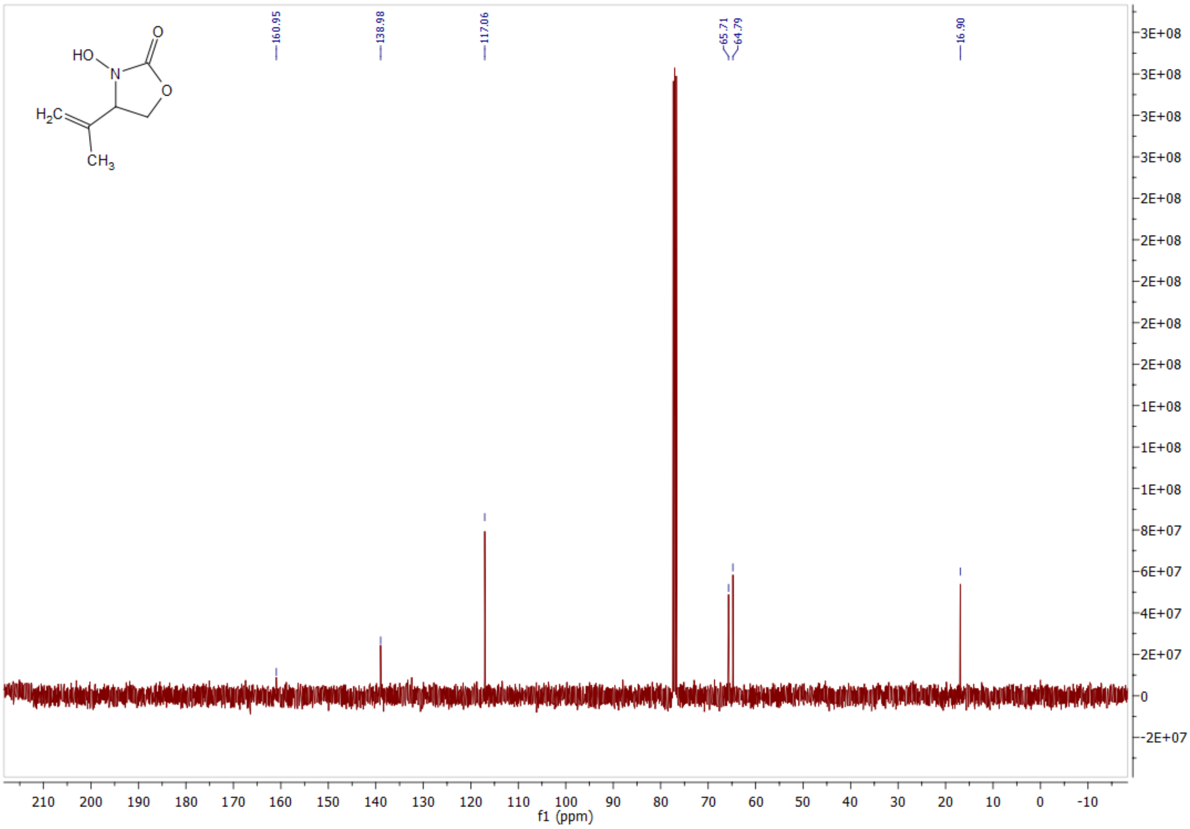
HO
N
O
O
H_2C
CH_3
160.95
138.98
117.06
65.71
64.79
16.90
f1 (ppm)

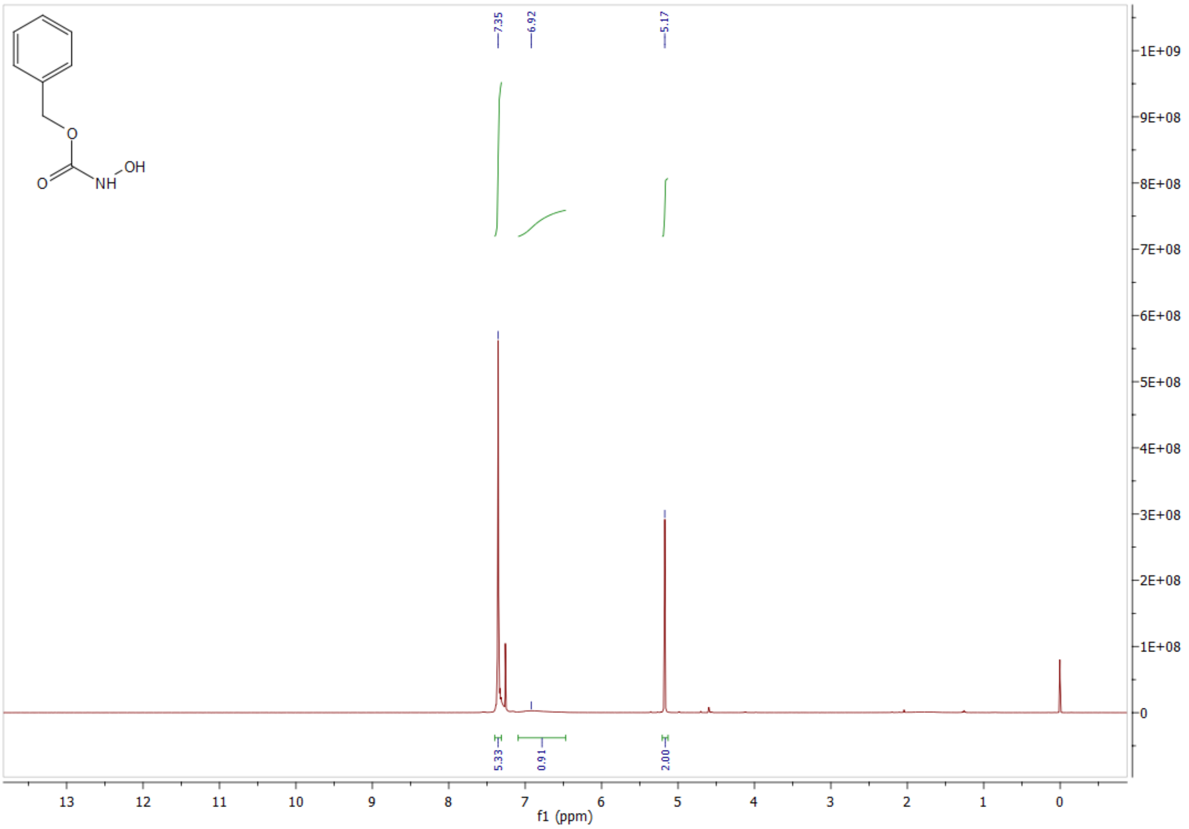

7.35
6.92
5.17
O
NH
OH
5.33
0.91
2.00
f1 (ppm)

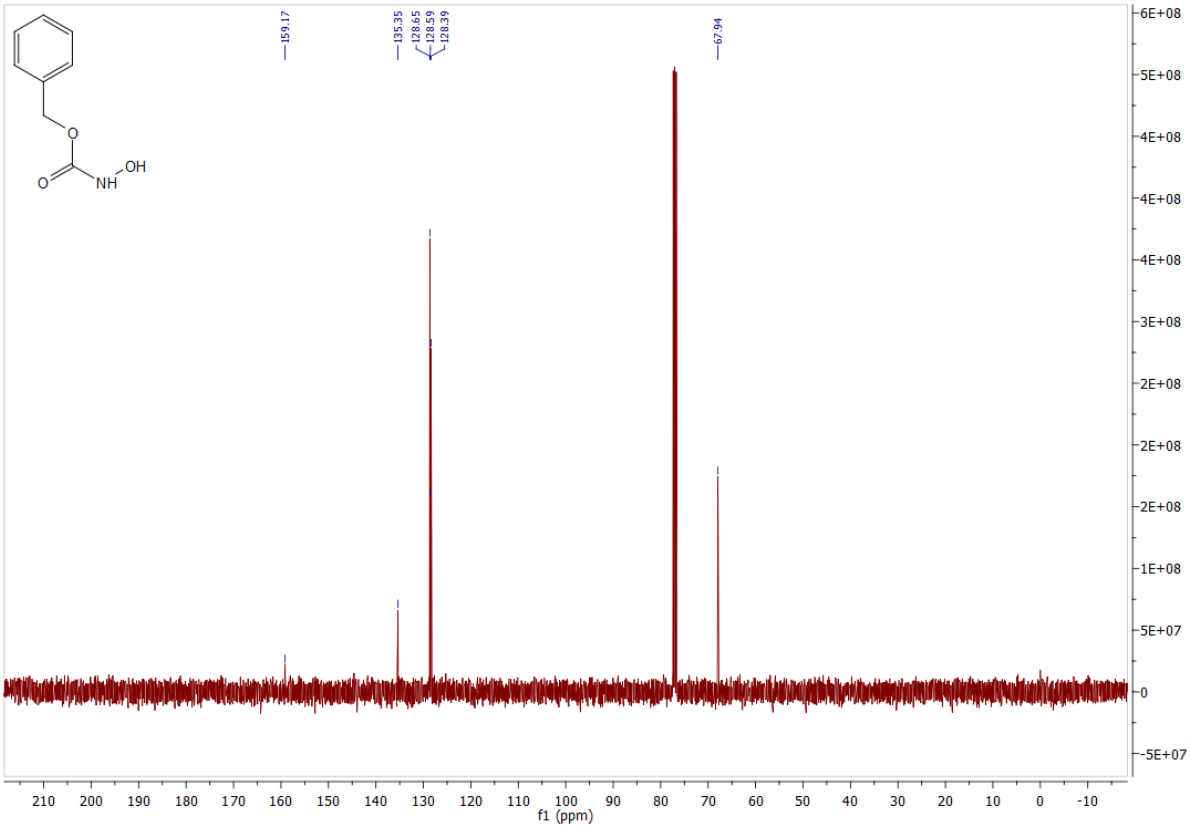

159.17
135.35
128.65
128.59
128.39
67.94
O
NH
OH
f1 (ppm)

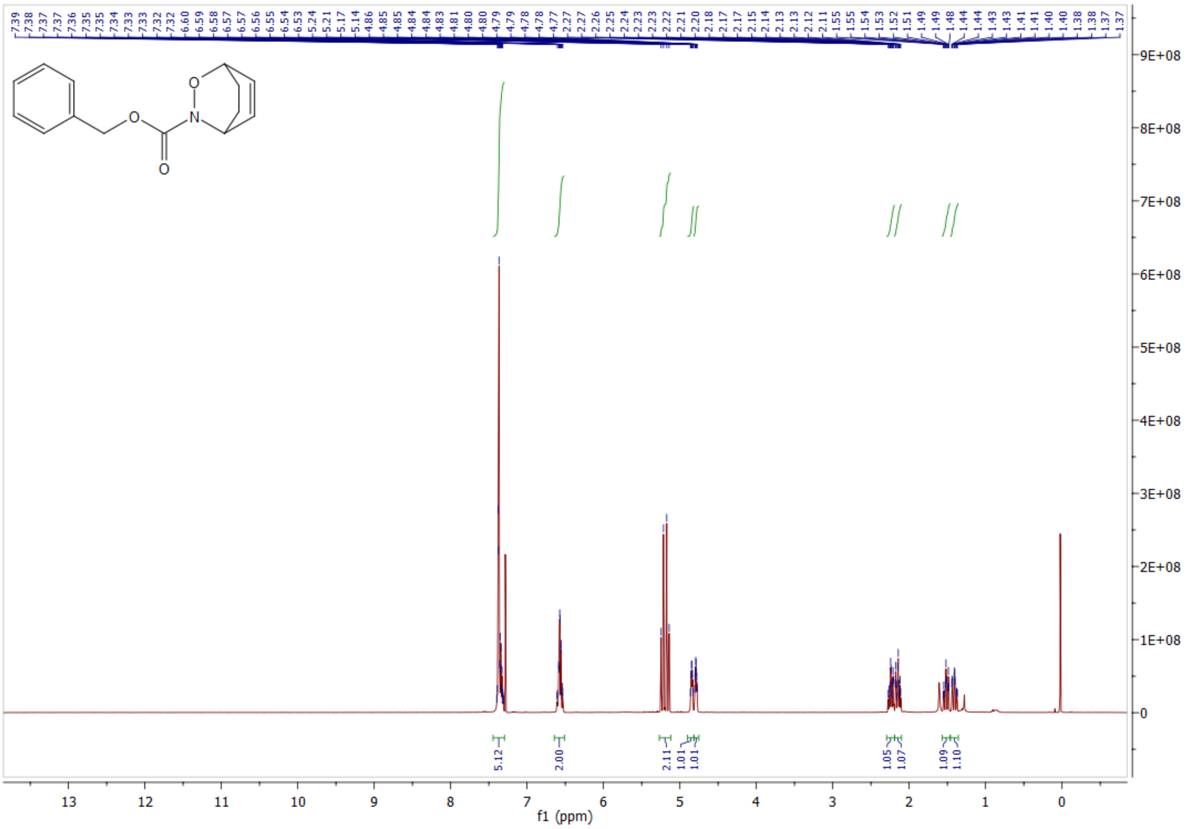
7.39 7.38 7.37 7.37 7.36 7.35 7.35 7.34 7.33 7.33 7.32 7.32 6.60 6.59 6.58 6.57 6.57 6.56 6.55 6.54 6.53 5.24 5.21 5.17 5.14 4.86 4.85 4.85 4.84 4.84 4.83 4.81 4.80 4.79 4.79 4.78 4.78 4.77 2.27 2.27 2.26 2.25 2.24 2.23 2.23 2.23 2.22 2.21 2.20 2.18 2.17 2.17 2.15 2.14 2.13 2.13 2.12 2.11 1.55 1.55 1.54 1.53 1.52 1.51 1.49 1.49 1.48 1.48 1.44 1.44 1.43 1.43 1.41 1.41 1.40 1.38 1.38 1.37 1.37
O
N
O
O
5.12
2.00
2.11
1.01
1.01
1.05
1.07
1.09
1.10
f1 (ppm)

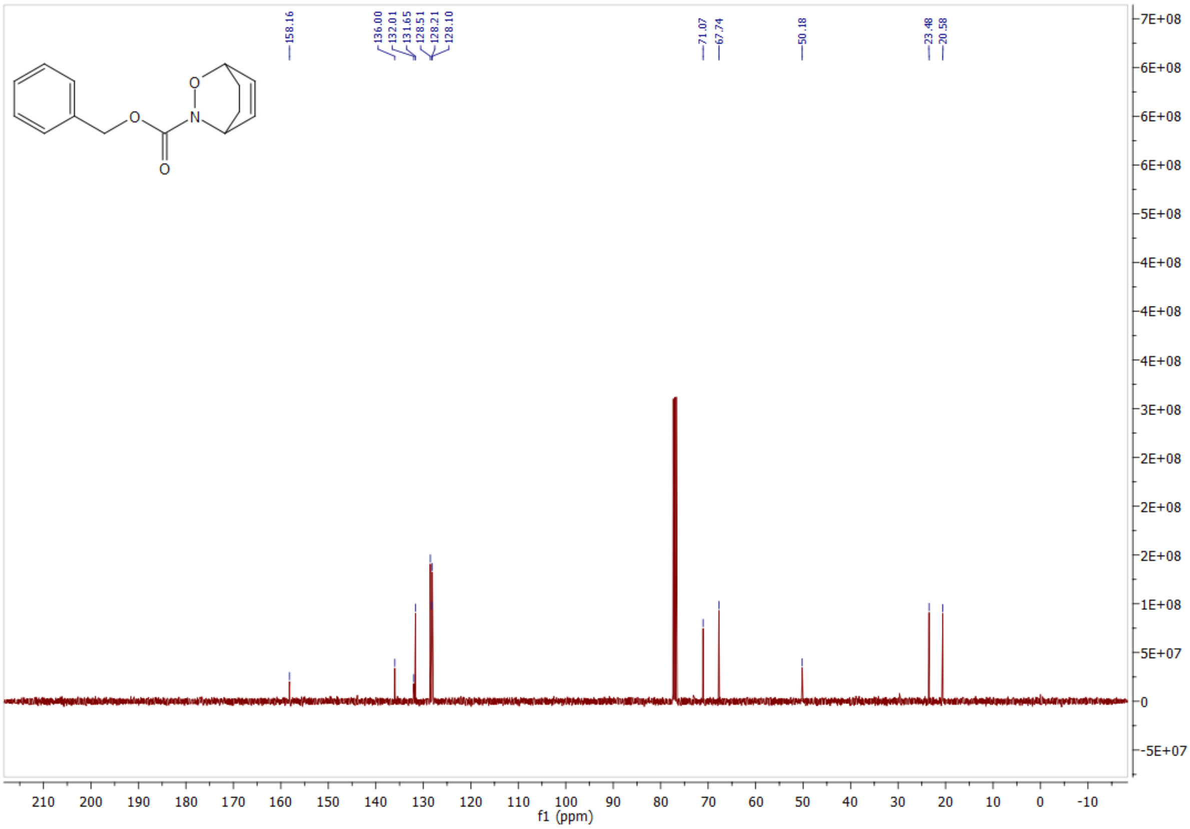
158.16
136.00 132.01 131.65 128.51 128.21 128.10
71.07
67.74
50.18
23.48
20.58
O
N
O
O
f1 (ppm)

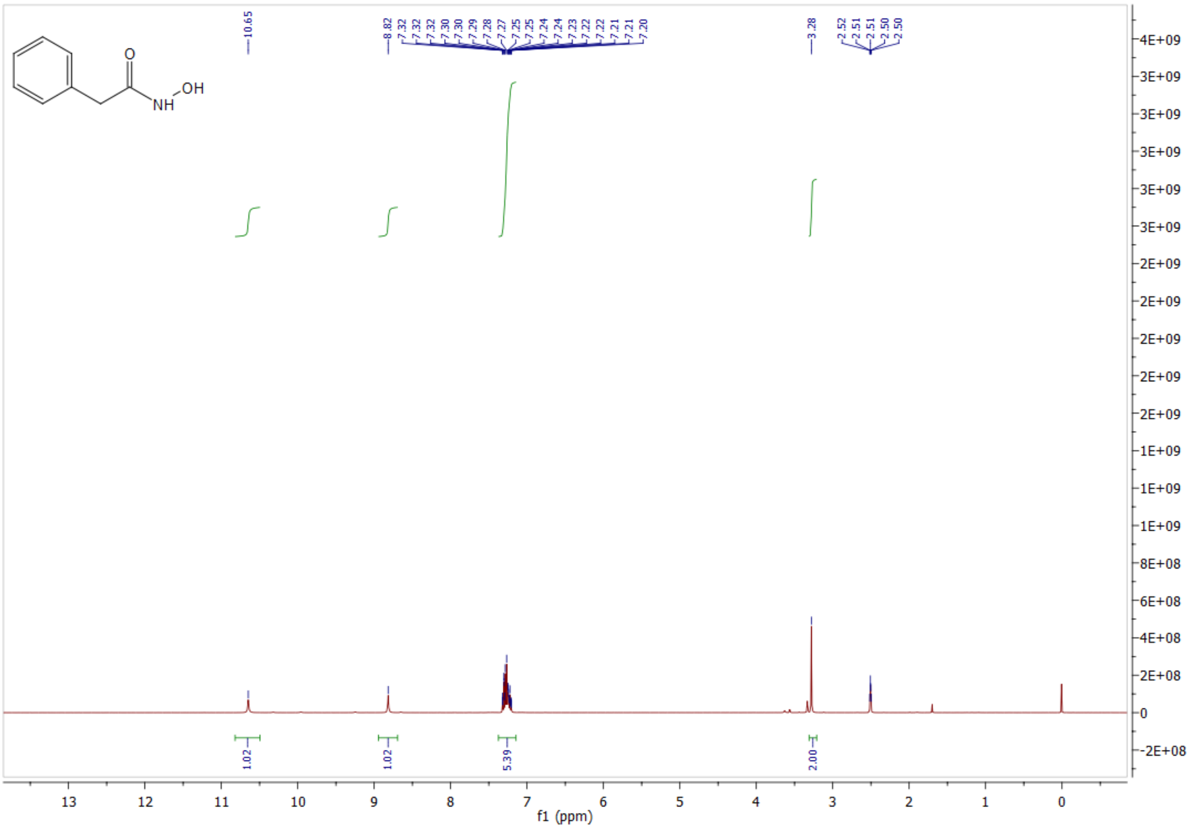
O
OH
NH
10.65
8.82
7.32 7.32 7.32 7.30 7.30 7.29 7.28 7.27 7.25 7.25 7.24 7.24 7.23 7.22 7.22 7.21 7.21 7.20
3.28
2.52 2.51 2.51 2.50 2.50
1.02
1.02
5.39
2.00
f1 (ppm)

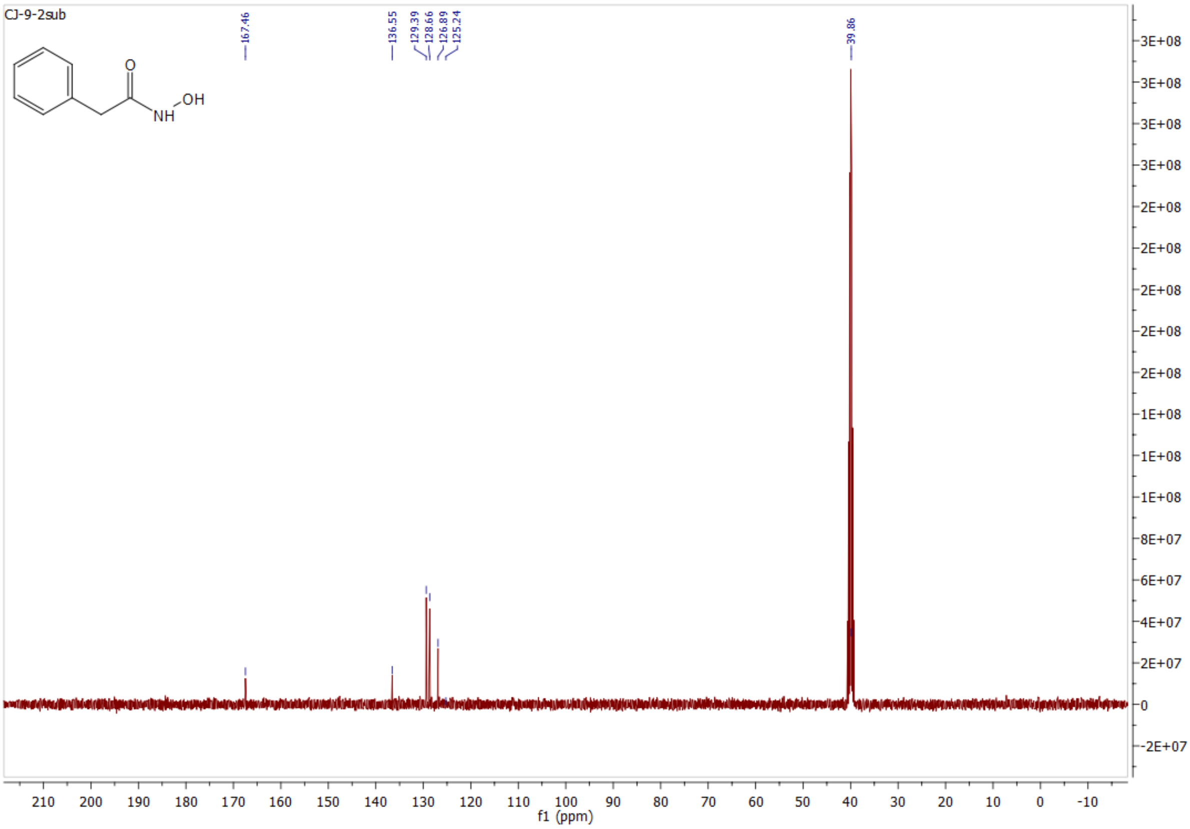
CJ-9-2sub
O
OH
NH
167.46
136.55
129.39 128.66 126.89 125.24
39.86
f1 (ppm)

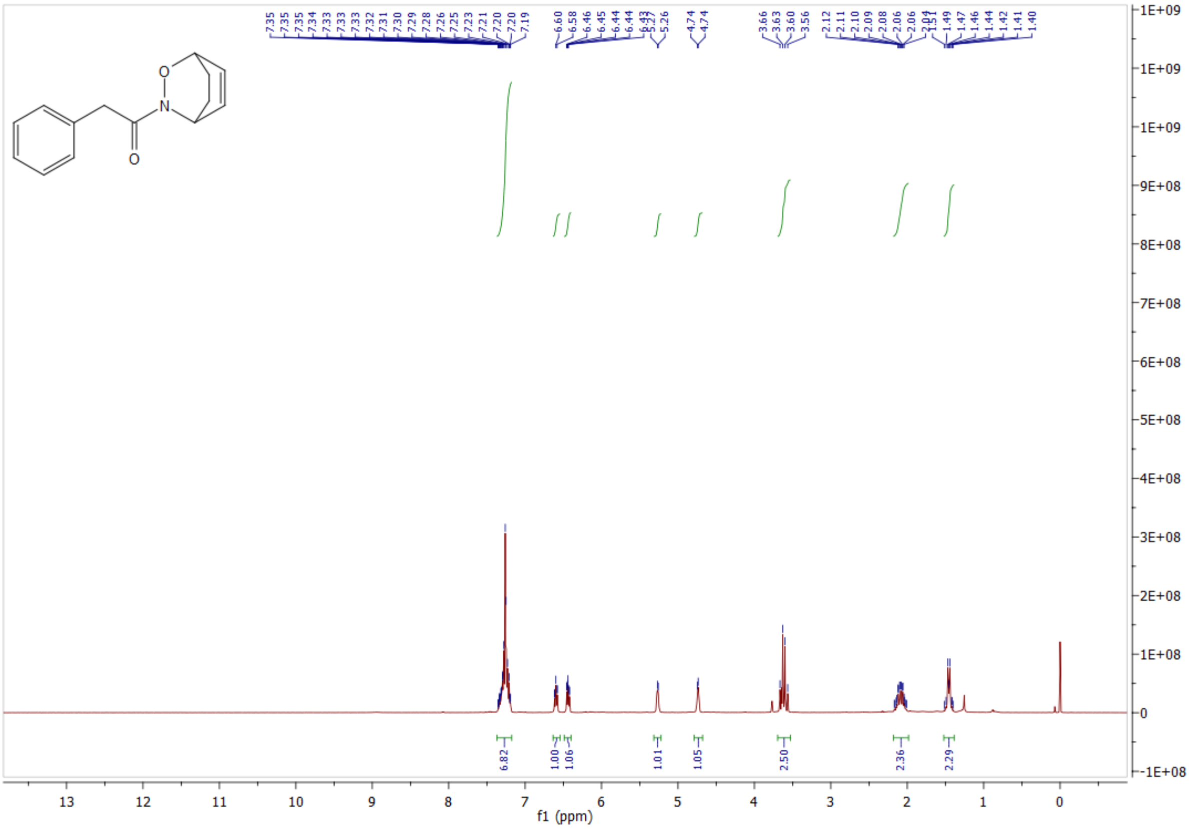

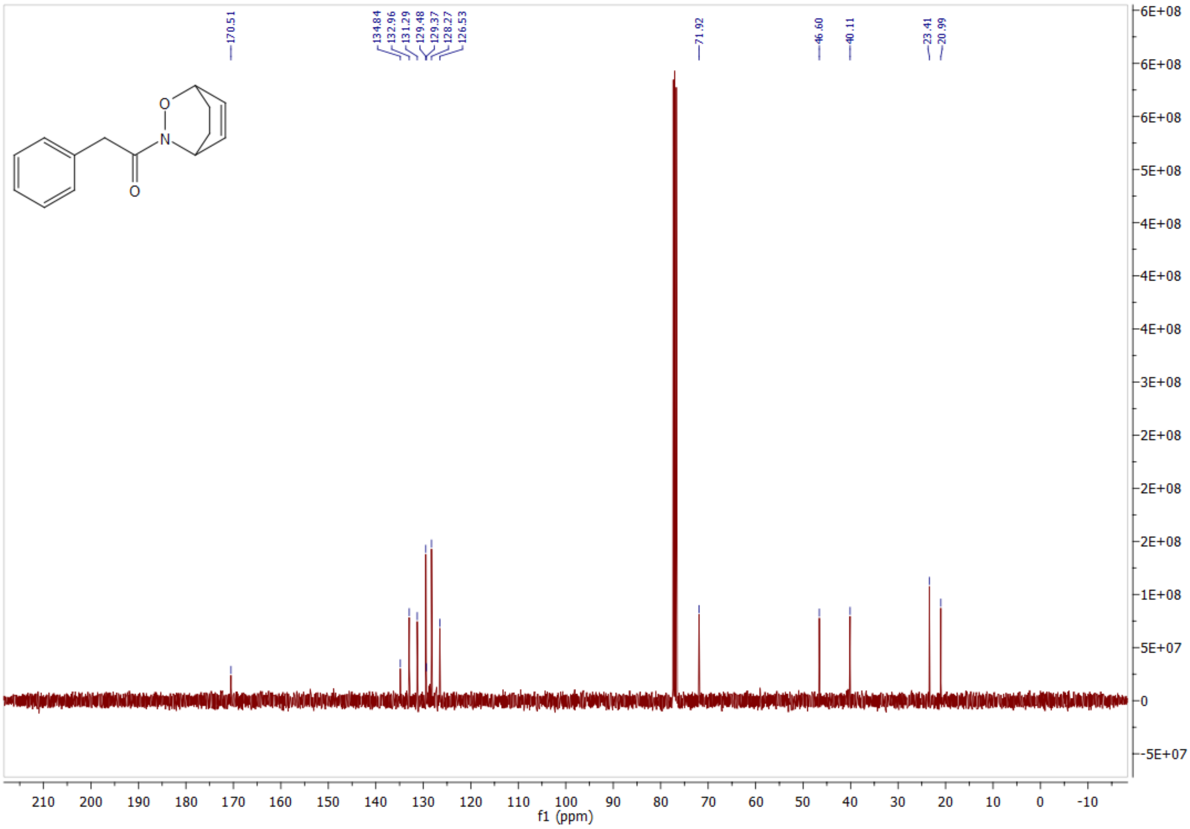

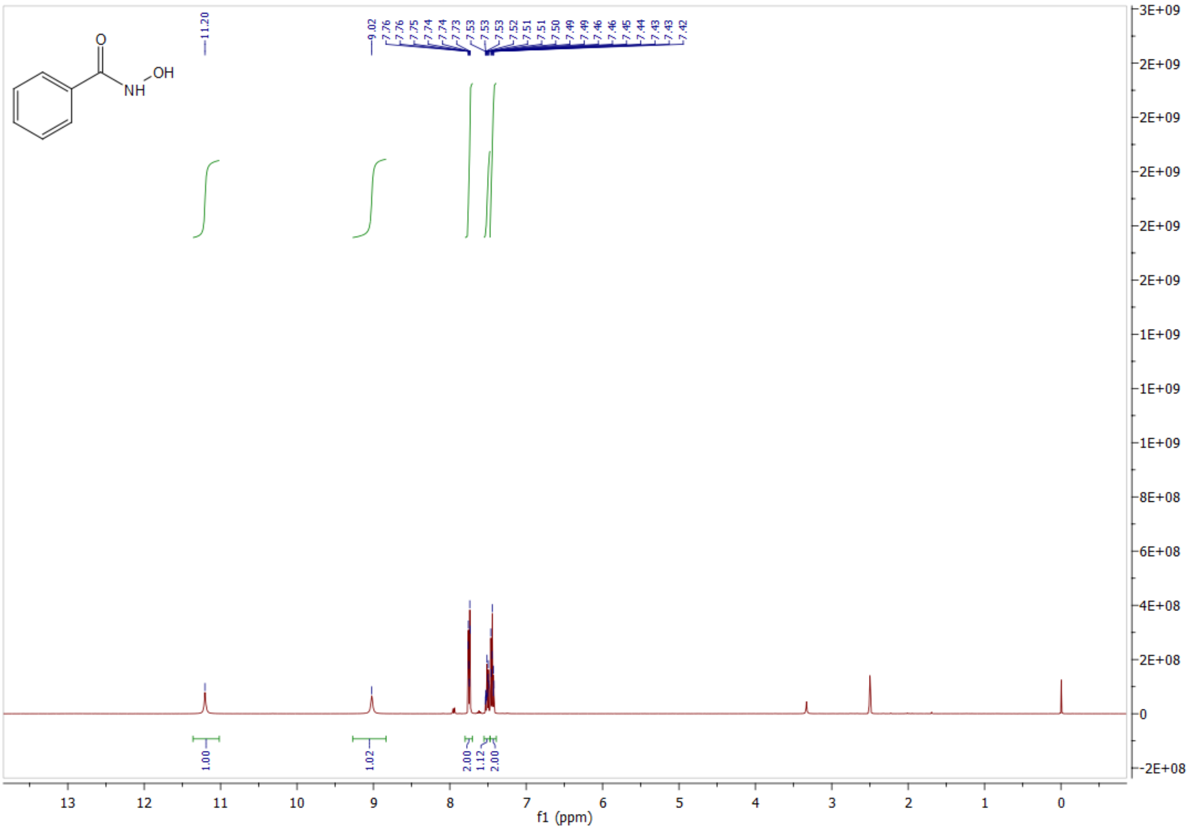
O
NH
OH
11.20
9.02
7.76
7.76
7.75
7.74
7.74
7.73
7.53
7.53
7.53
7.52
7.51
7.50
7.49
7.49
7.46
7.45
7.44
7.43
7.43
7.42
1.00
1.02
2.00
1.12
2.00
f1 (ppm)

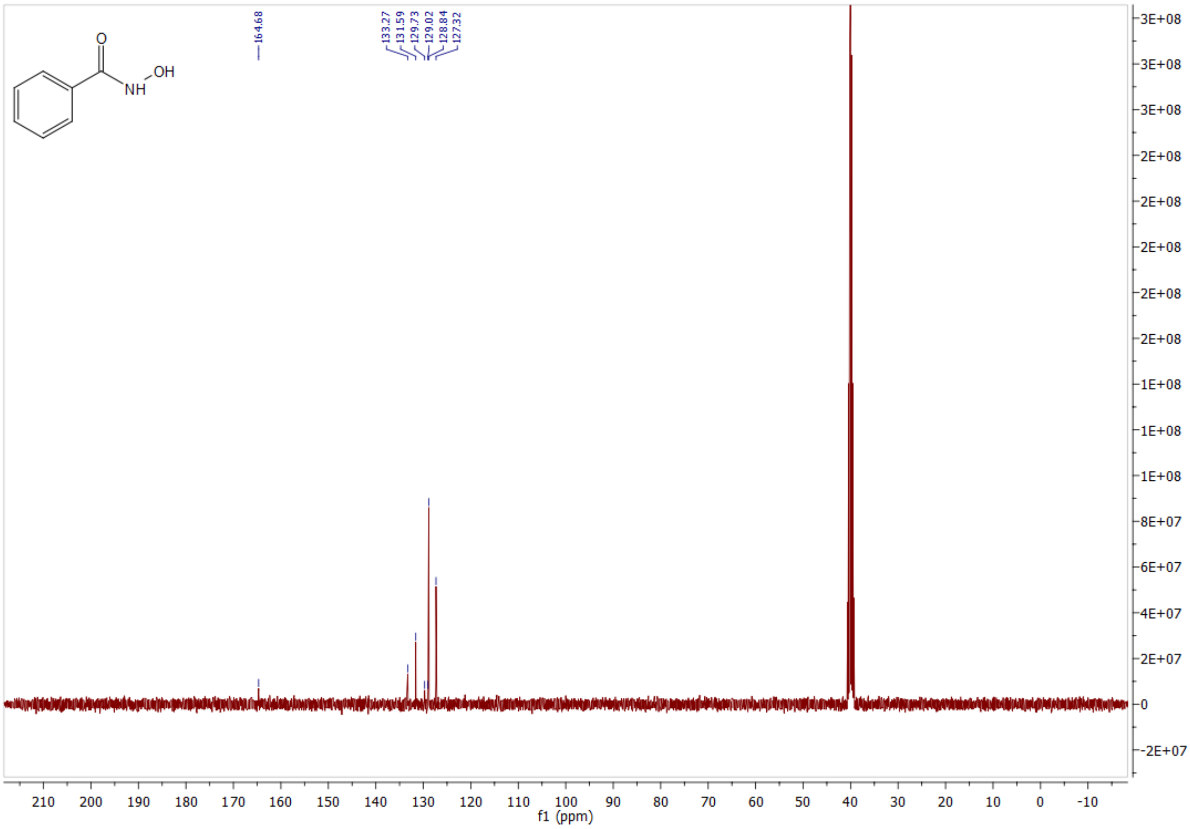
O
NH
OH
164.68
133.27
131.59
129.73
129.02
128.84
127.32
f1 (ppm)

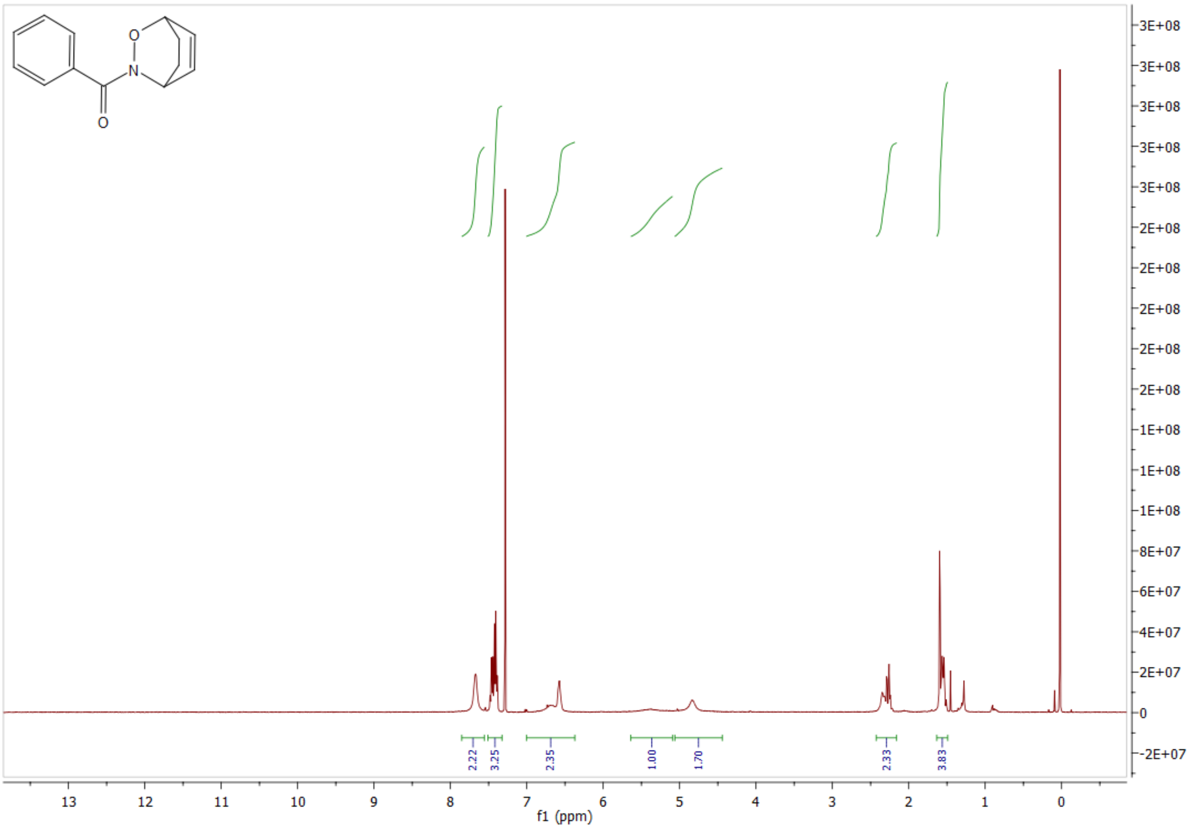
O
N
O
2.22
3.25
2.35
1.00
1.70
2.33
3.83
f1 (ppm)

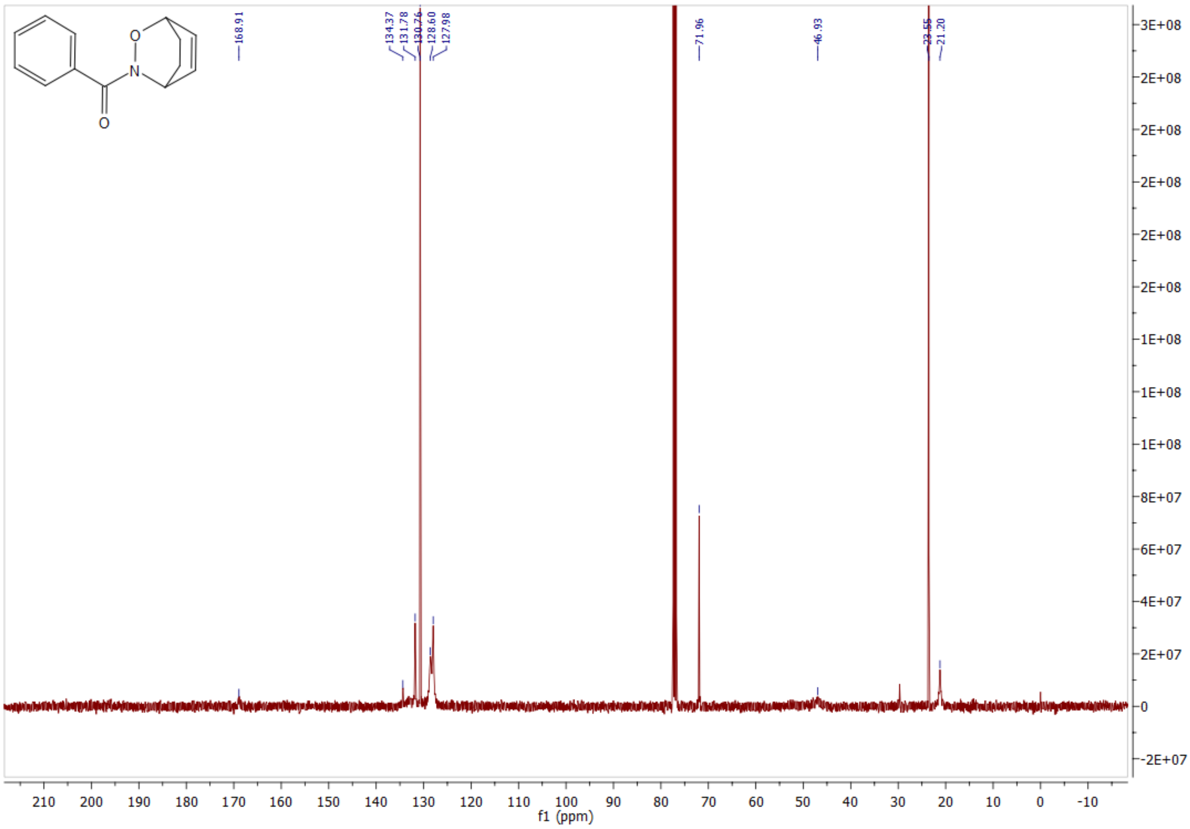
O
N
O
168.91
134.37
131.78
130.76
128.60
127.98
71.96
46.93
21.20
f1 (ppm)

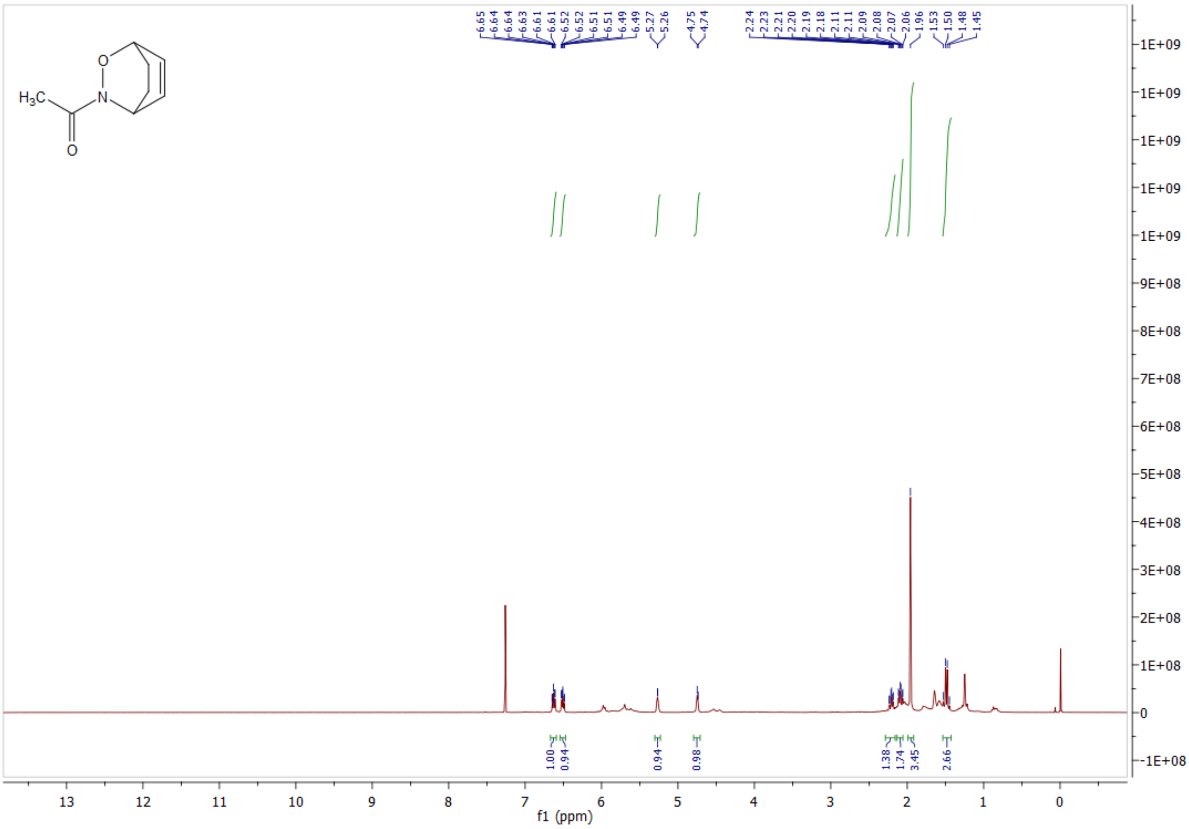
H3C
N
O
O
6.65
6.64
6.64
6.63
6.61
6.61
6.52
6.52
6.51
6.51
6.49
6.49
5.27
5.26
4.75
4.74
2.24
2.23
2.21
2.20
2.19
2.18
2.11
2.11
2.09
2.08
2.07
2.06
1.96
1.53
1.50
1.48
1.45
1.00
0.94
0.94
0.98
1.38
1.74
3.45
2.66
f1 (ppm)

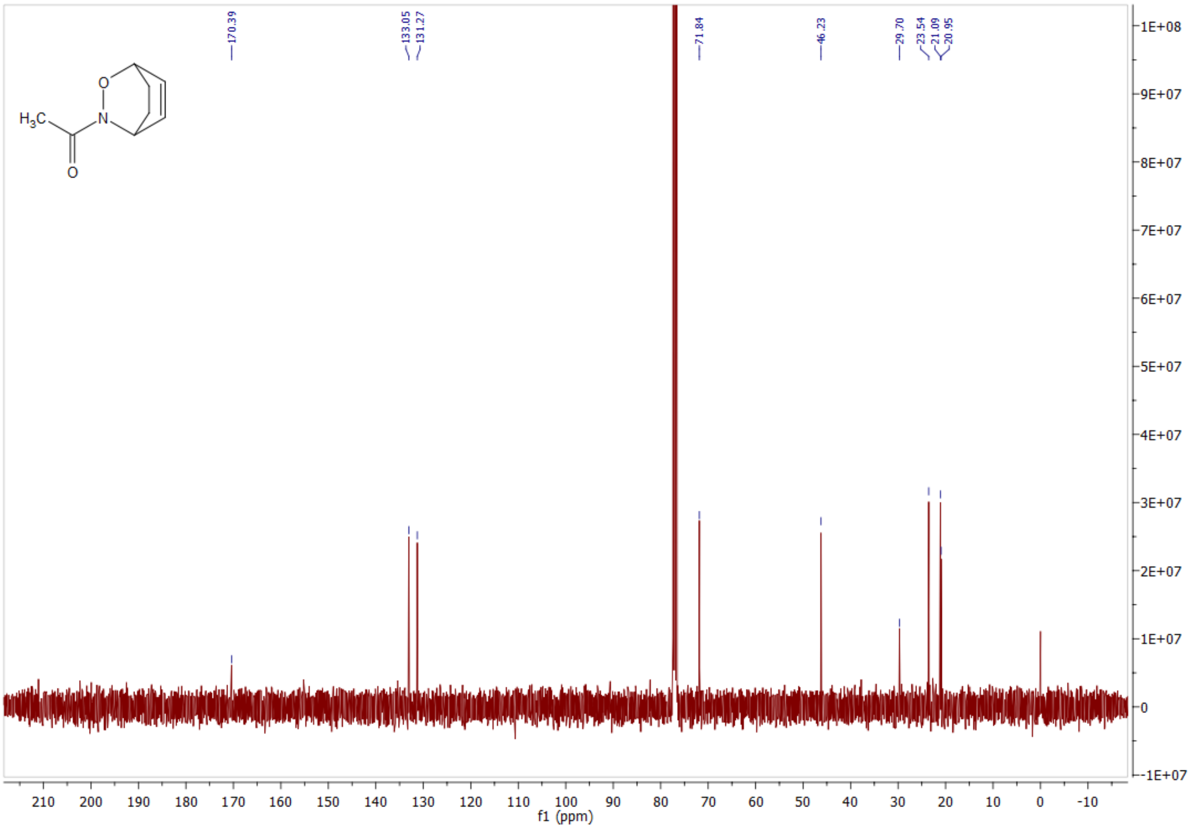
H3C
N
O
O
170.39
133.05
131.27
71.84
46.23
29.70
23.54
21.09
20.95
f1 (ppm)

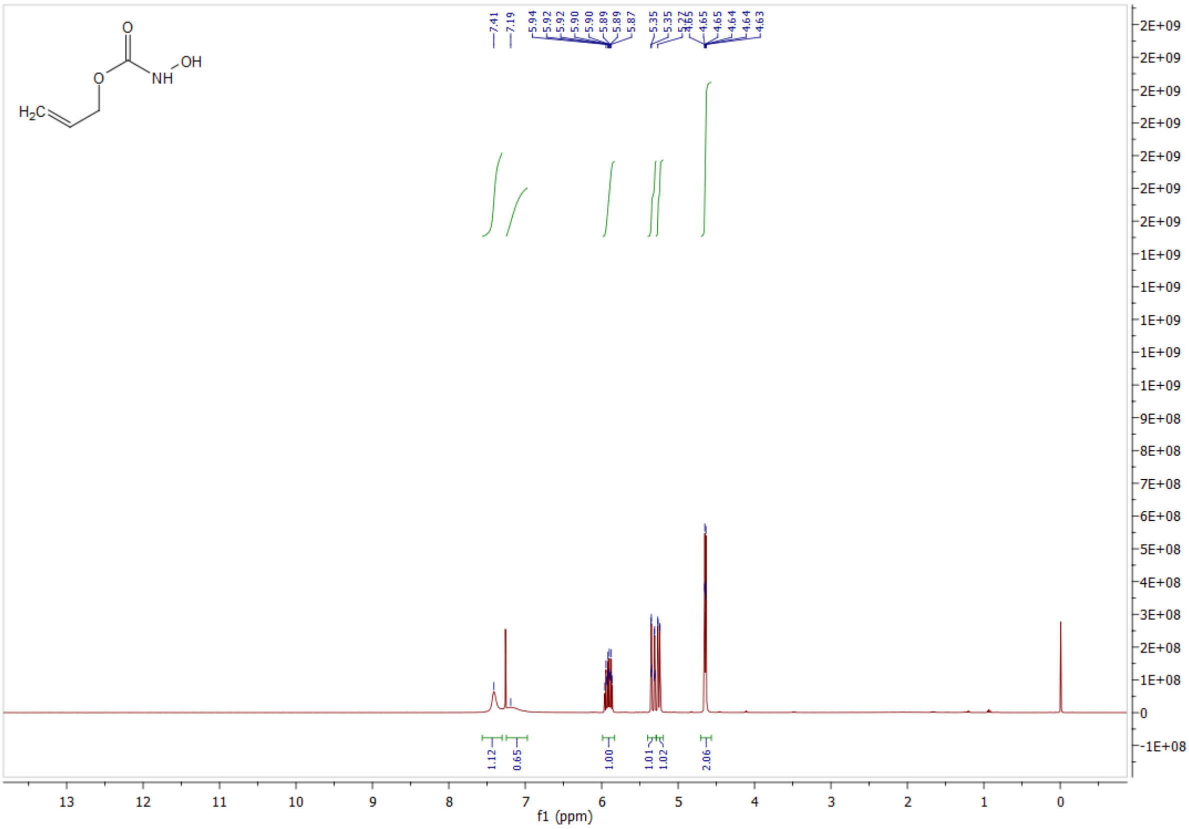
O
NH
OH
H2C
7.41
7.19
1.12
0.65
1.00
1.01
1.02
2.06
f1 (ppm)

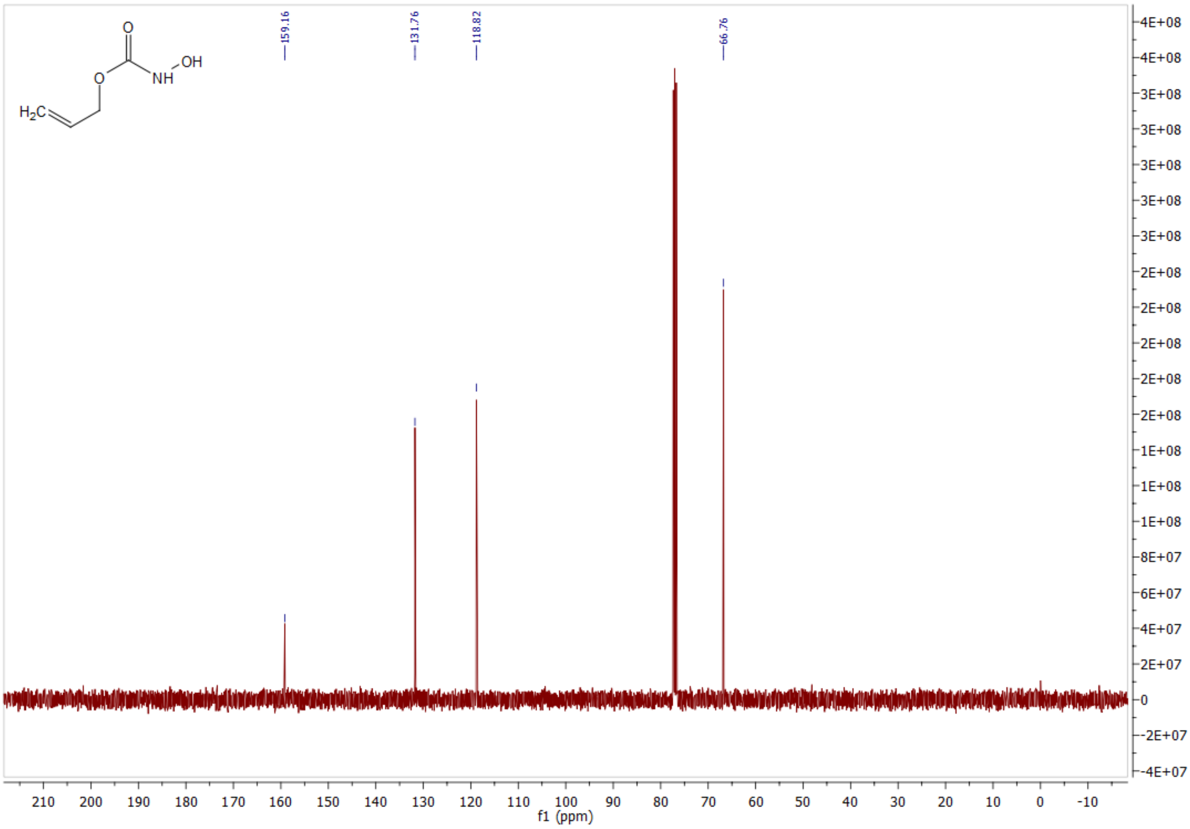
O
NH
OH
H2C
159.16
131.76
118.82
66.76
f1 (ppm)

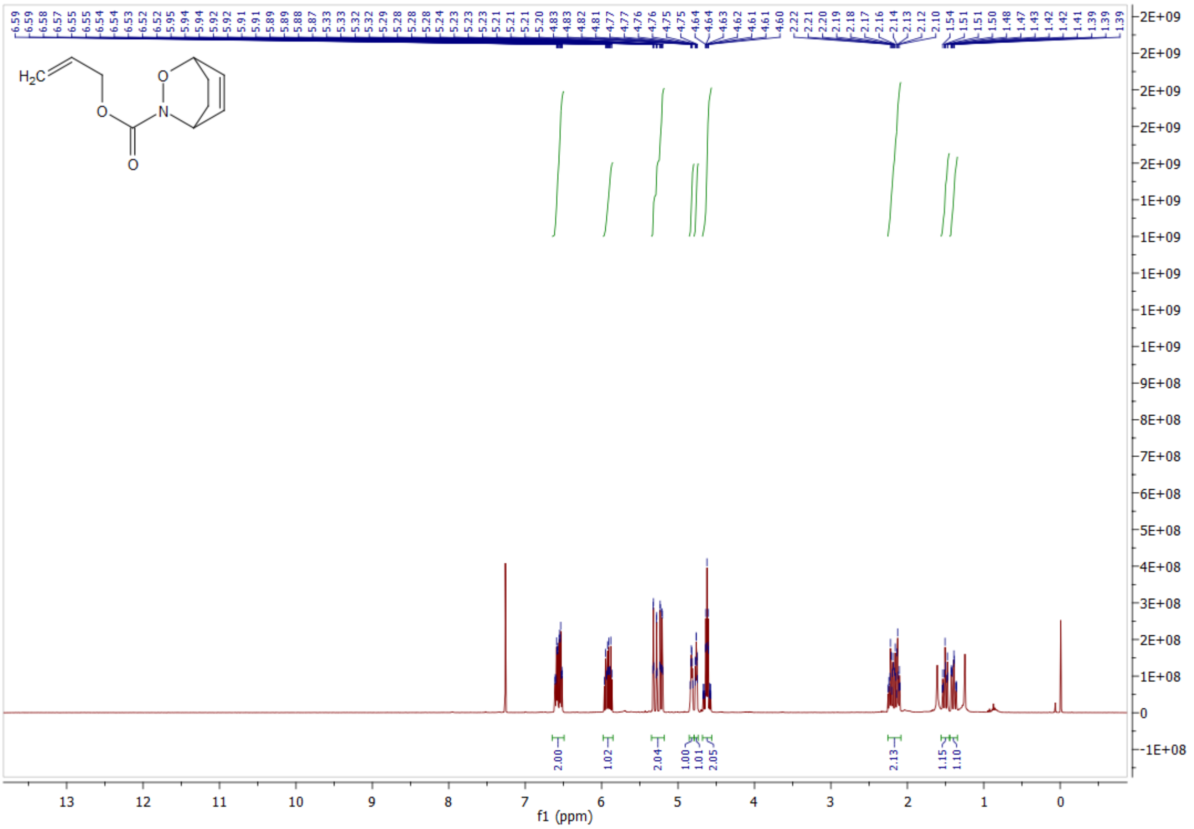
6.59 6.59 6.58 6.57 6.55 6.55 6.54 6.54 6.53 6.52 6.52 5.95 5.94 5.94 5.92 5.92 5.91 5.91 5.89 5.88 5.87 5.33 5.33 5.32 5.32 5.29 5.28 5.28 5.24 5.23 5.23 5.23 5.21 5.21 5.21 5.20 4.83 4.83 4.82 4.81 4.77 4.77 4.76 4.76 4.75 4.75 4.64 4.64 4.62 4.61 4.61 4.60 2.22 2.21 2.20 2.19 2.18 2.17 2.16 2.14 2.13 2.12 2.10 1.54 1.51 1.51 1.50 1.48 1.47 1.43 1.42 1.41 1.39 1.39
H_2C
O
N
O
O
2.00
1.02
2.04
1.00
1.01
2.05
2.13
1.15
1.10
f1 (ppm)

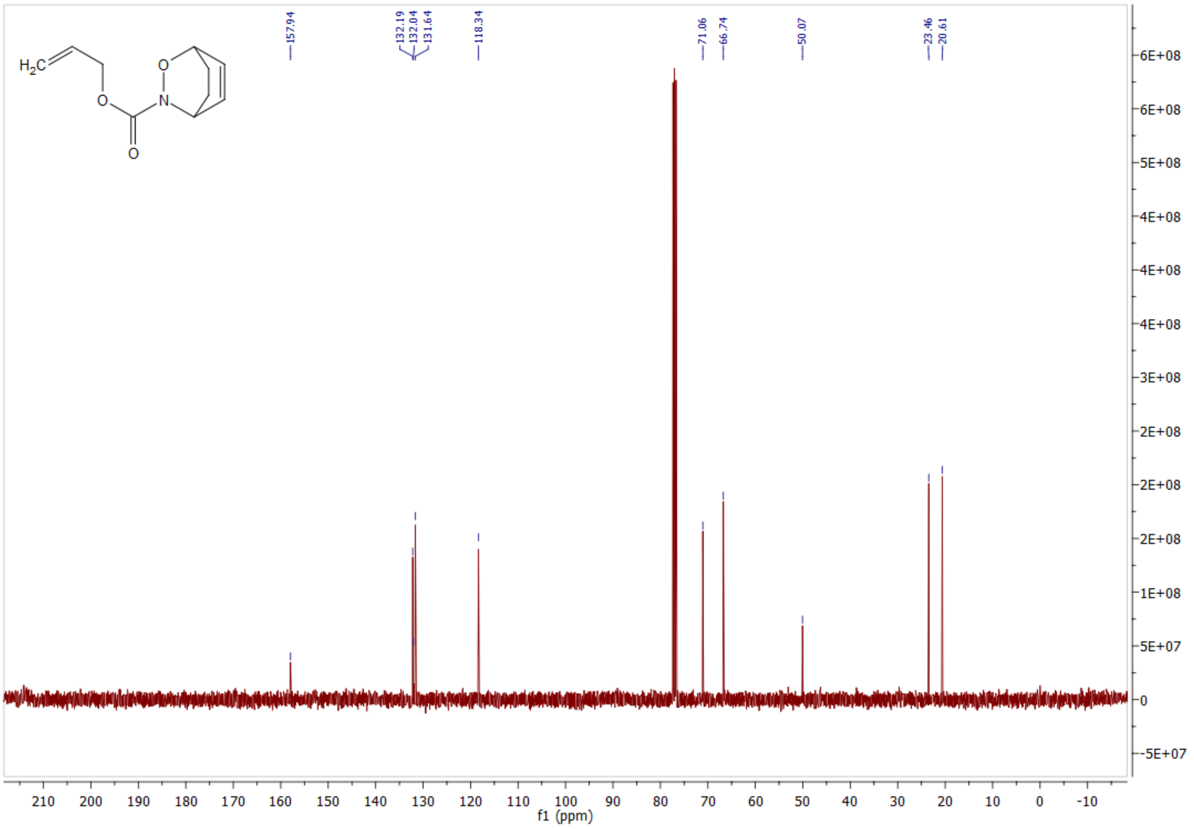
157.94
132.19
132.04
131.64
118.34
71.06
66.74
50.07
23.46
20.61
H_2C
O
N
O
O
f1 (ppm)

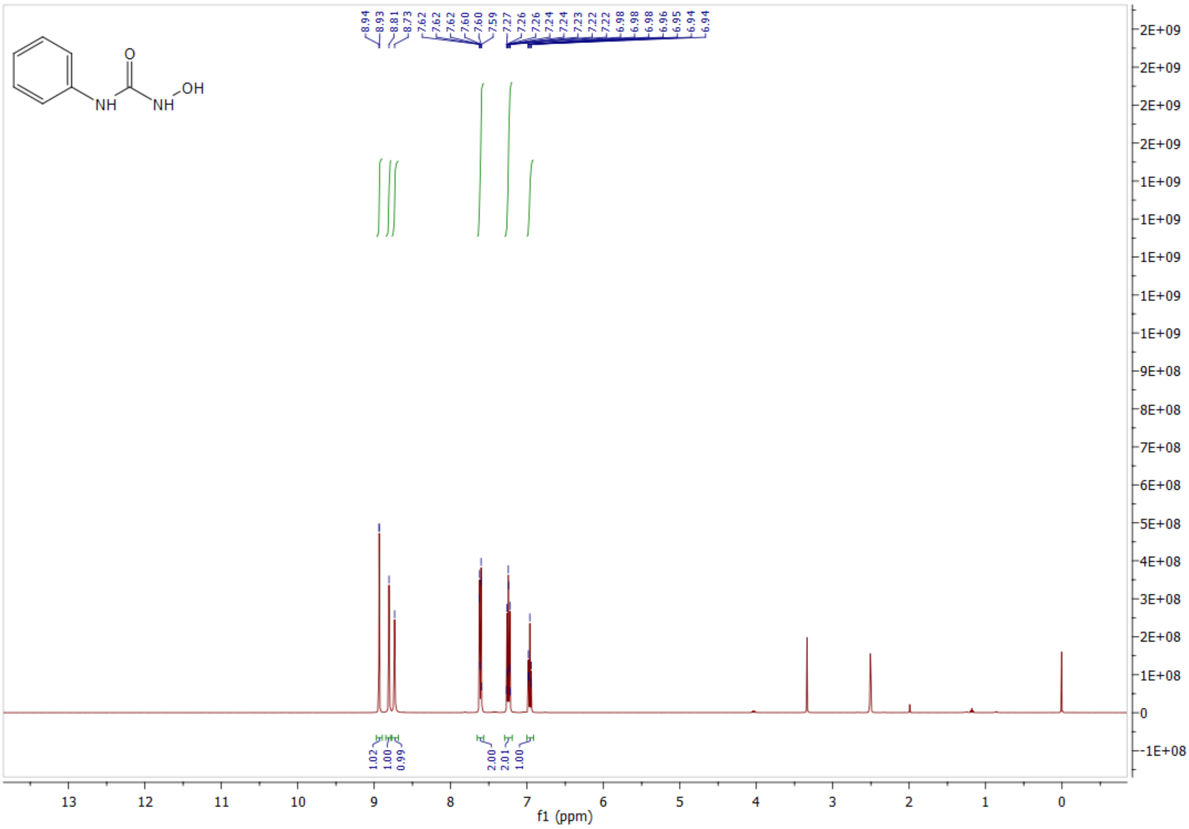

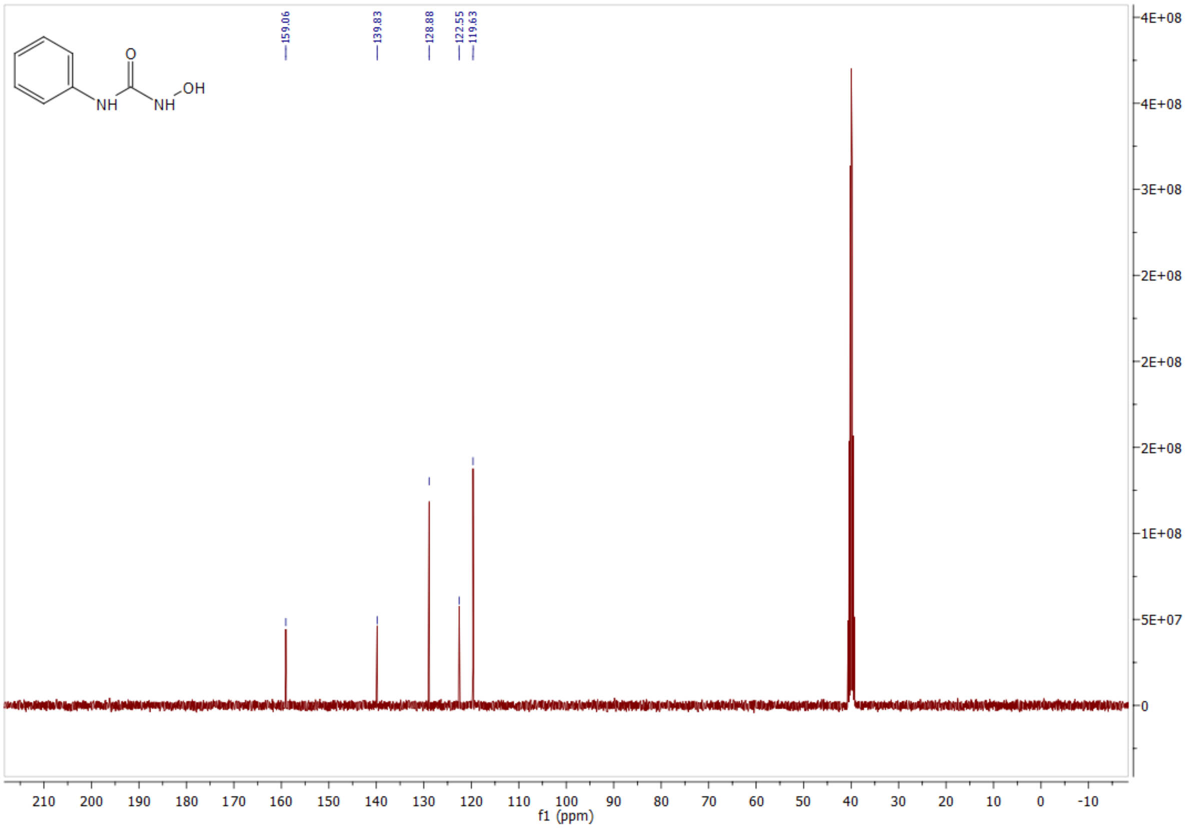

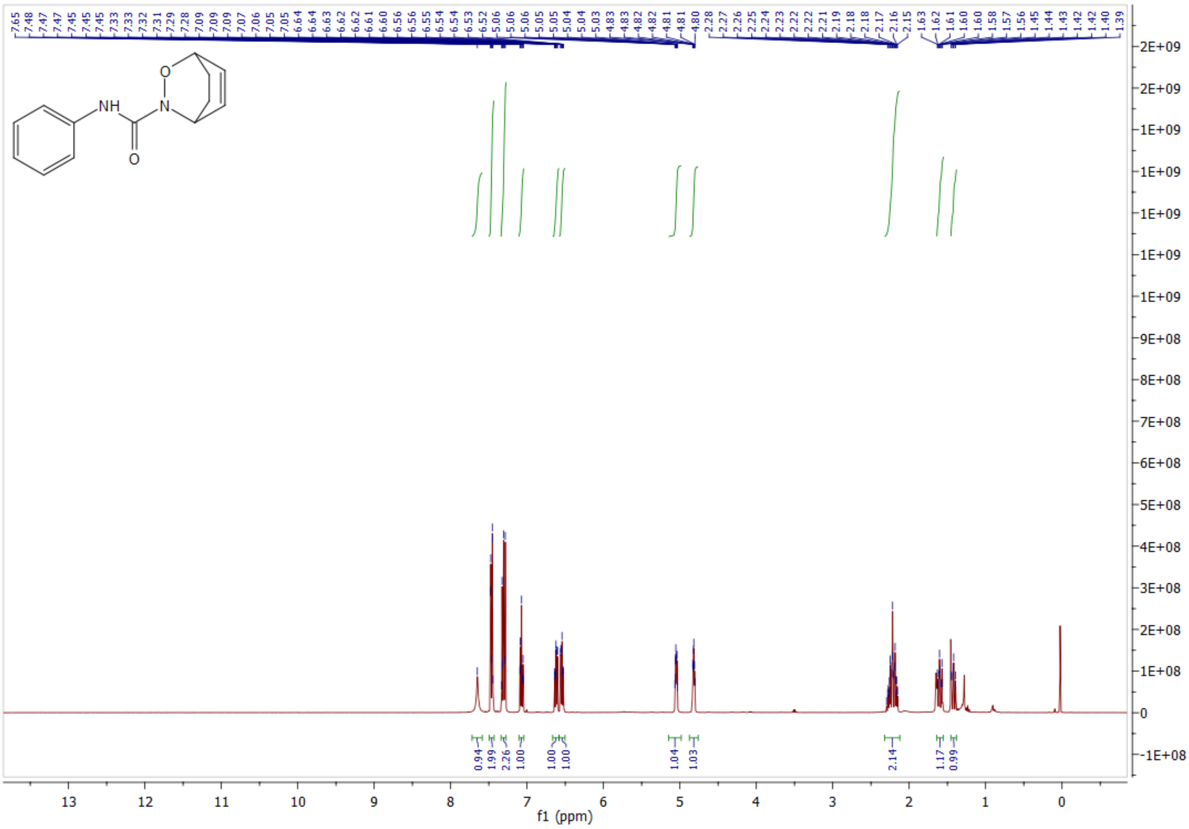
7.65 7.48 7.47 7.47 7.45 7.45 7.45 7.33 7.33 7.32 7.31 7.29 7.28 7.09 7.09 7.09 7.07 7.06 7.05 7.05 6.64 6.64 6.63 6.62 6.62 6.61 6.60 6.56 6.56 6.55 6.54 6.54 6.53 6.52 5.06 5.06 5.06 5.05 5.05 5.04 5.04 5.03 4.83 4.83 4.82 4.82 4.81 4.81 4.80 2.28 2.27 2.26 2.25 2.24 2.23 2.22 2.22 2.21 2.19 2.18 2.18 2.17 2.16 2.15 1.63 1.62 1.61 1.60 1.60 1.58 1.57 1.56 1.45 1.44 1.43 1.42 1.40 1.39
NH
O
N
O
0.94 1.99 2.26 1.00 1.00 1.00 1.04 1.03 2.14 1.17 0.99
f1 (ppm)

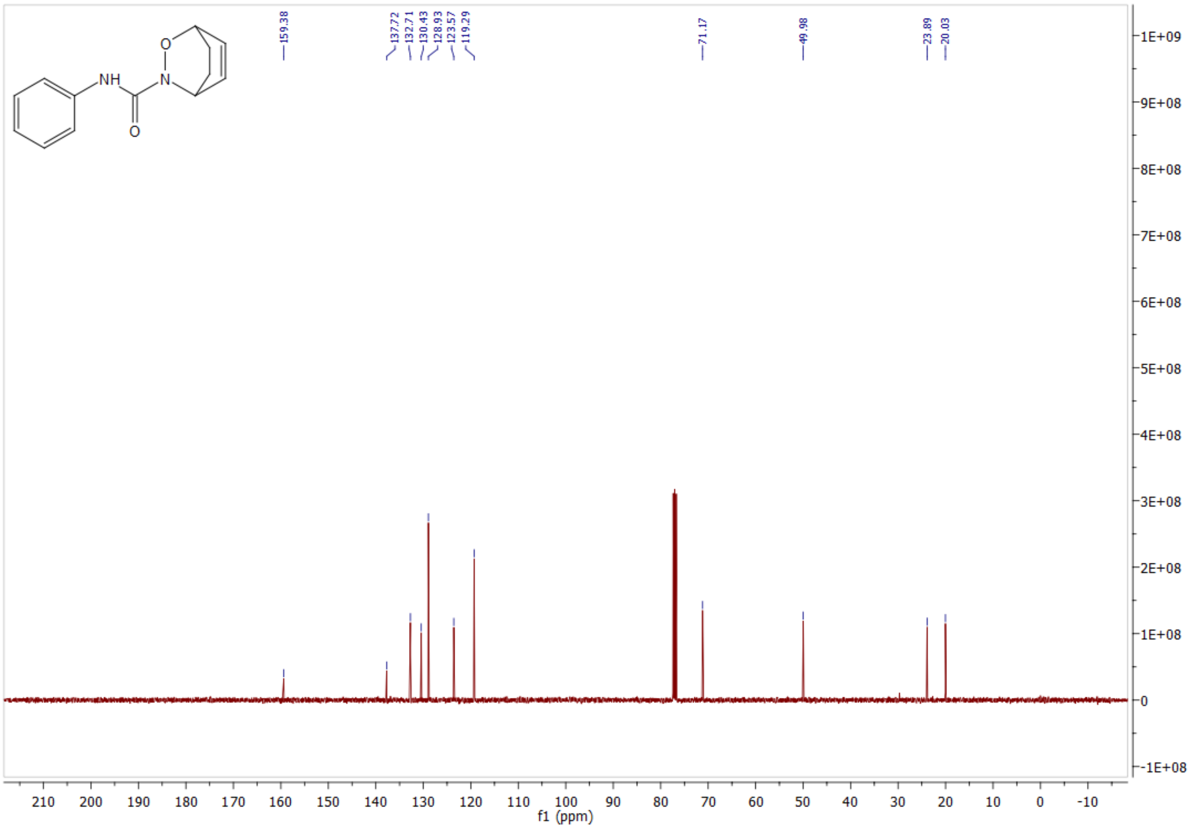
159.38
137.72 132.71 130.43 128.93 123.57 119.29
71.17
49.98
23.89 20.03
NH
O
N
O
f1 (ppm)

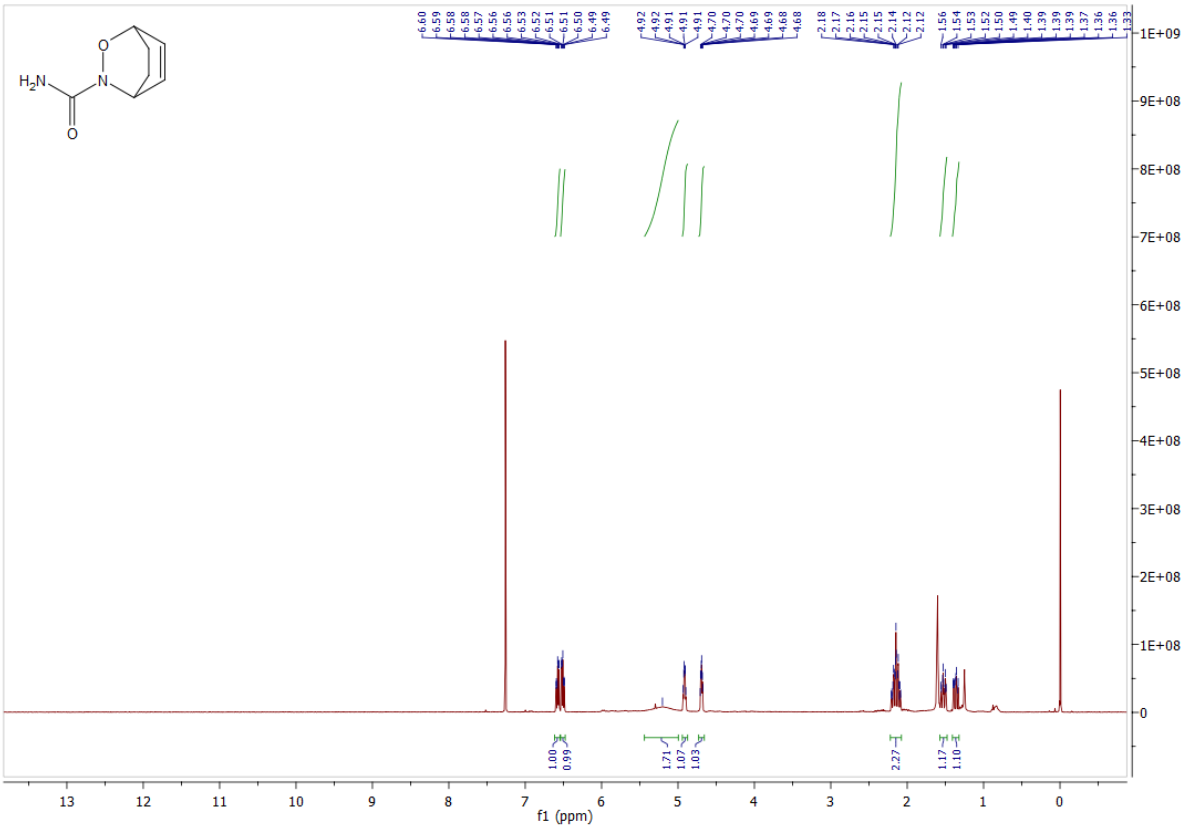
H2N
N
O
O
6.60
6.59
6.58
6.57
6.56
6.56
6.53
6.52
6.51
6.51
6.50
6.49
6.49
4.92
4.92
4.91
4.91
4.91
4.70
4.70
4.70
4.69
4.69
4.68
4.68
2.18
2.17
2.16
2.15
2.15
2.14
2.12
2.12
1.56
1.54
1.53
1.52
1.50
1.49
1.40
1.39
1.39
1.39
1.37
1.36
1.33
1.00
0.99
1.71
1.07
1.03
2.27
1.17
1.10
f1 (ppm)

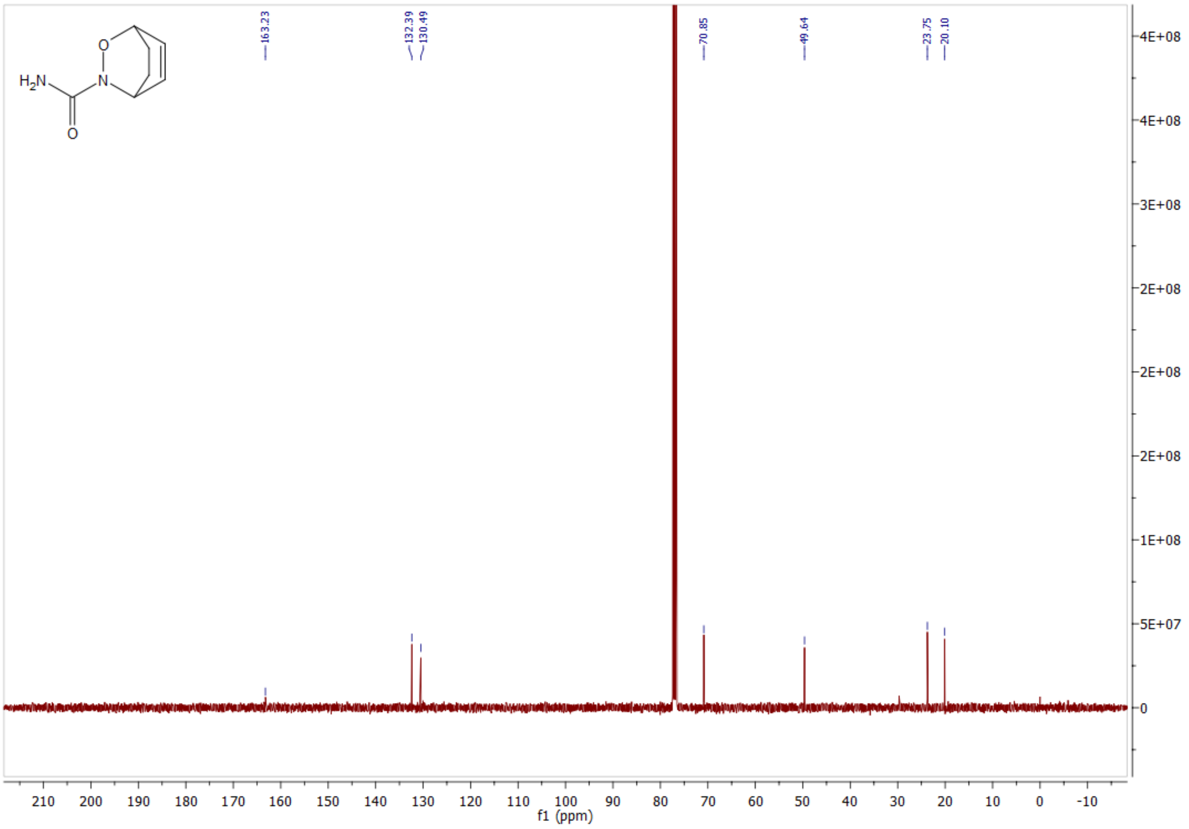
H2N
N
O
O
163.23
132.39
130.49
70.85
49.64
23.75
20.10
f1 (ppm)

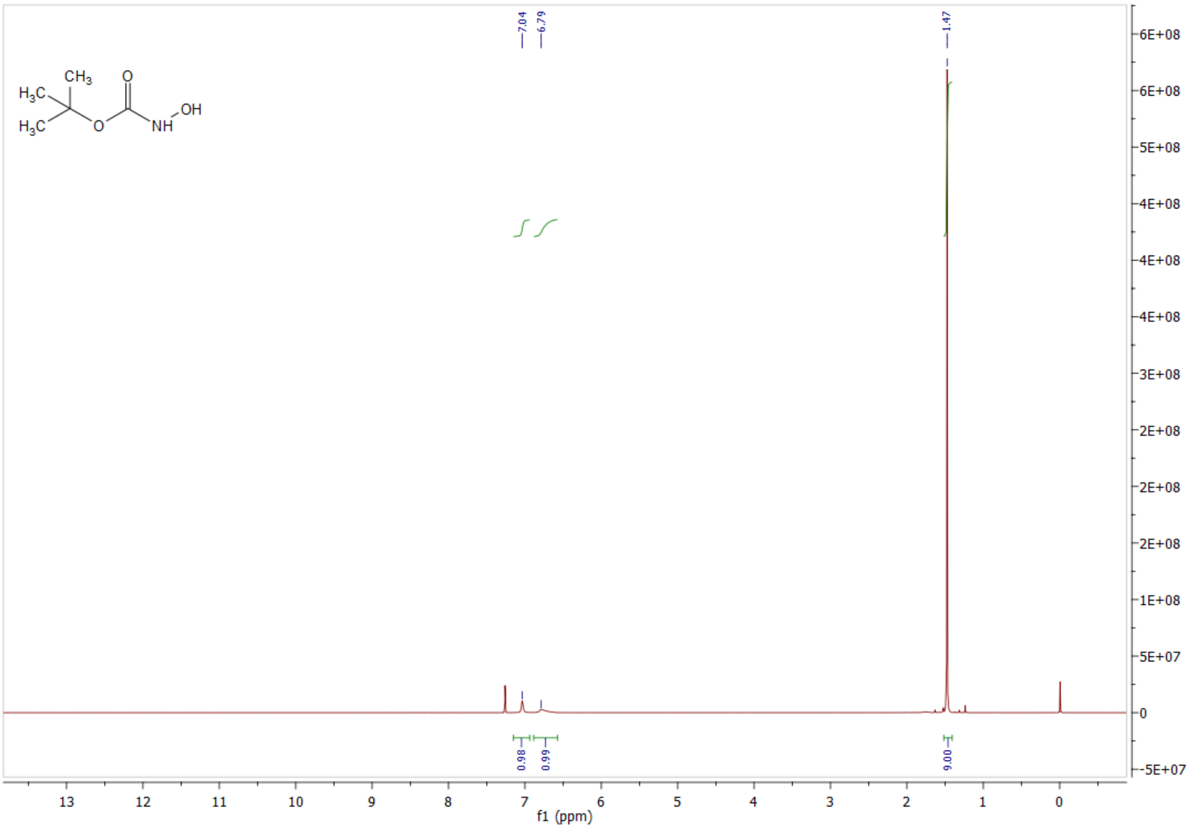

CH3
H3C
H3C
O
O
NH
OH
7.04
6.79
1.47
0.98
0.99
9.00
f1 (ppm)

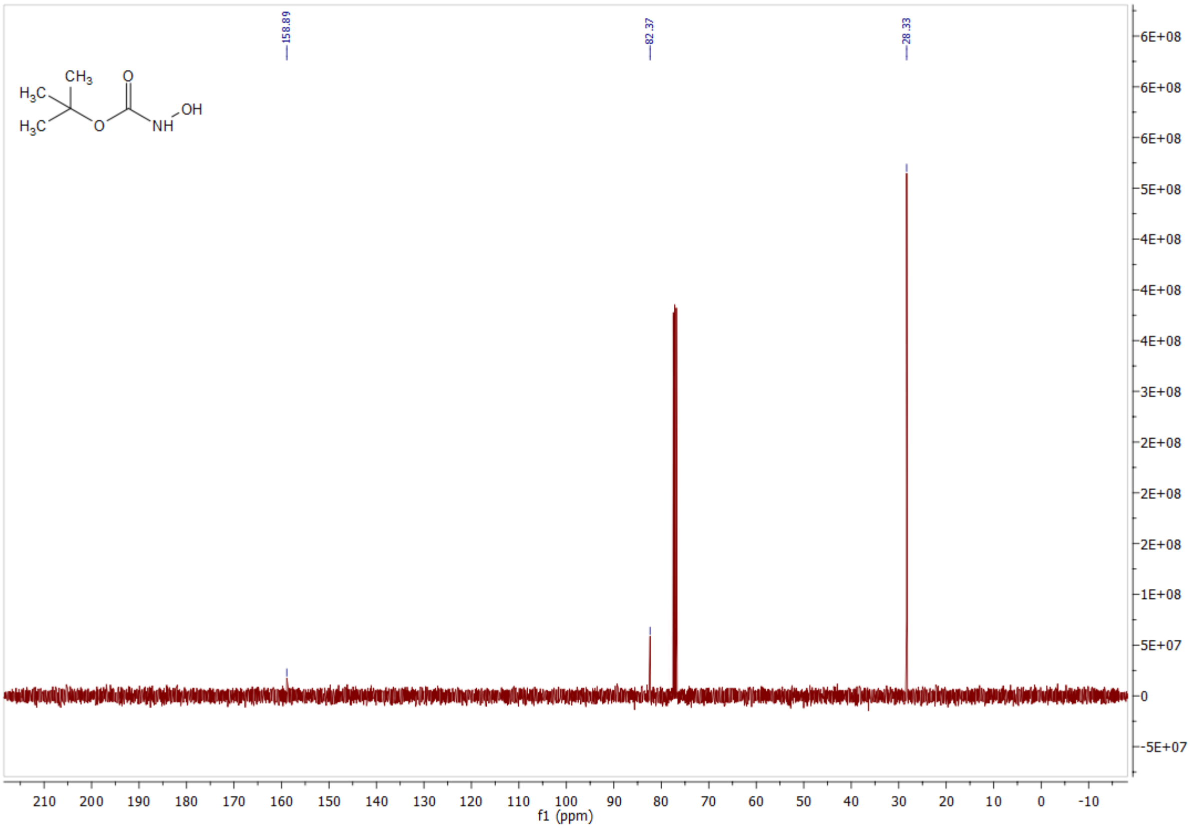

CH3
H3C
H3C
O
O
NH
OH
158.89
82.37
28.33
f1 (ppm)

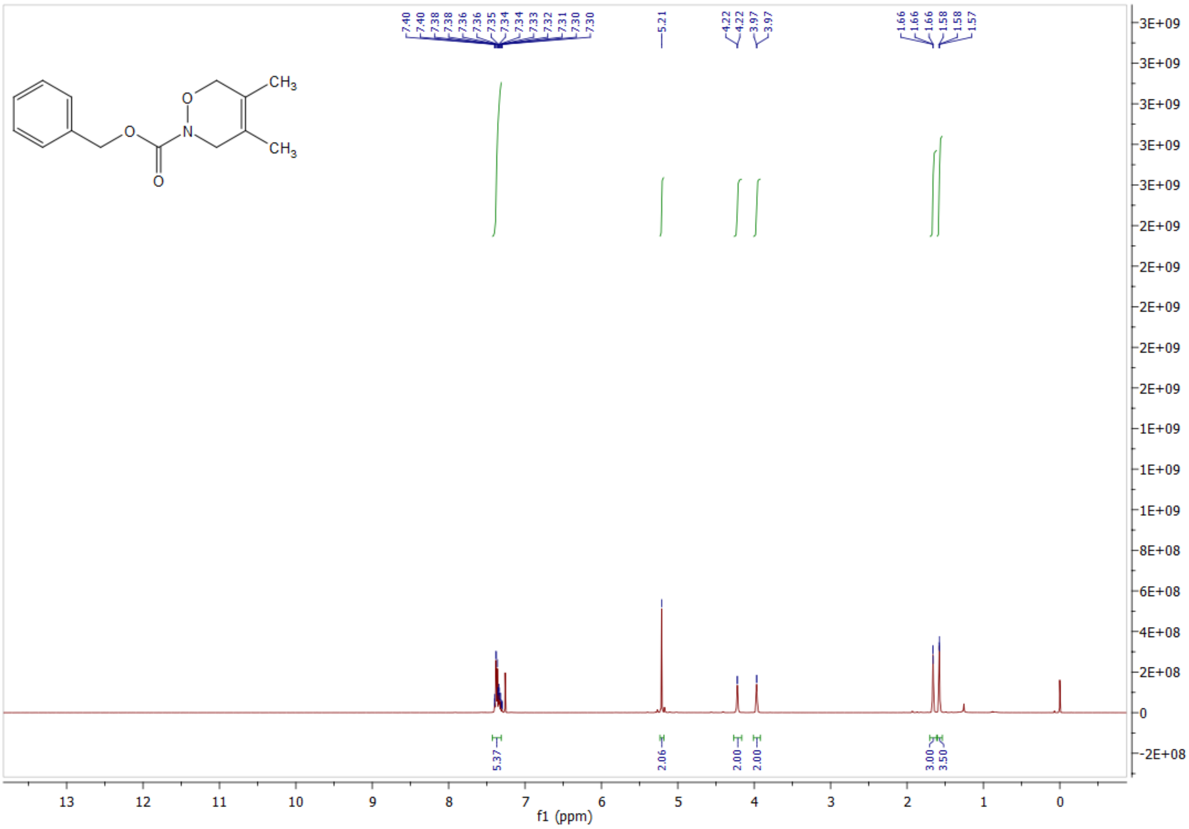
7.40
7.40
7.38
7.38
7.36
7.36
7.35
7.34
7.34
7.33
7.33
7.32
7.31
7.30
7.30
5.21
4.22
4.22
3.97
3.97
1.66
1.66
1.66
1.66
1.58
1.58
1.57
CH3
CH3
O
N
O
O
5.37
2.06
2.00
2.00
3.00
3.50
f1 (ppm)

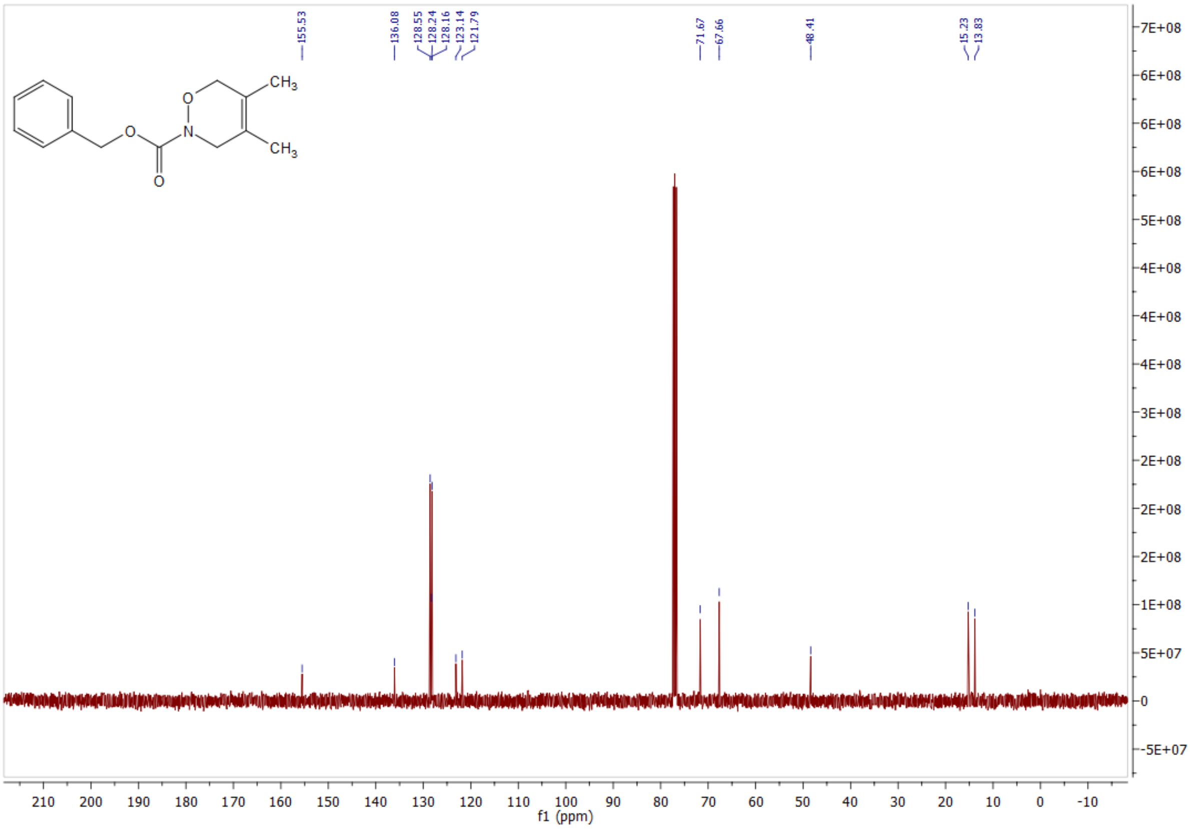
155.53
136.08
128.55
128.24
128.16
123.14
121.79
71.67
67.66
48.41
15.23
13.83
CH3
CH3
O
N
O
O
f1 (ppm)

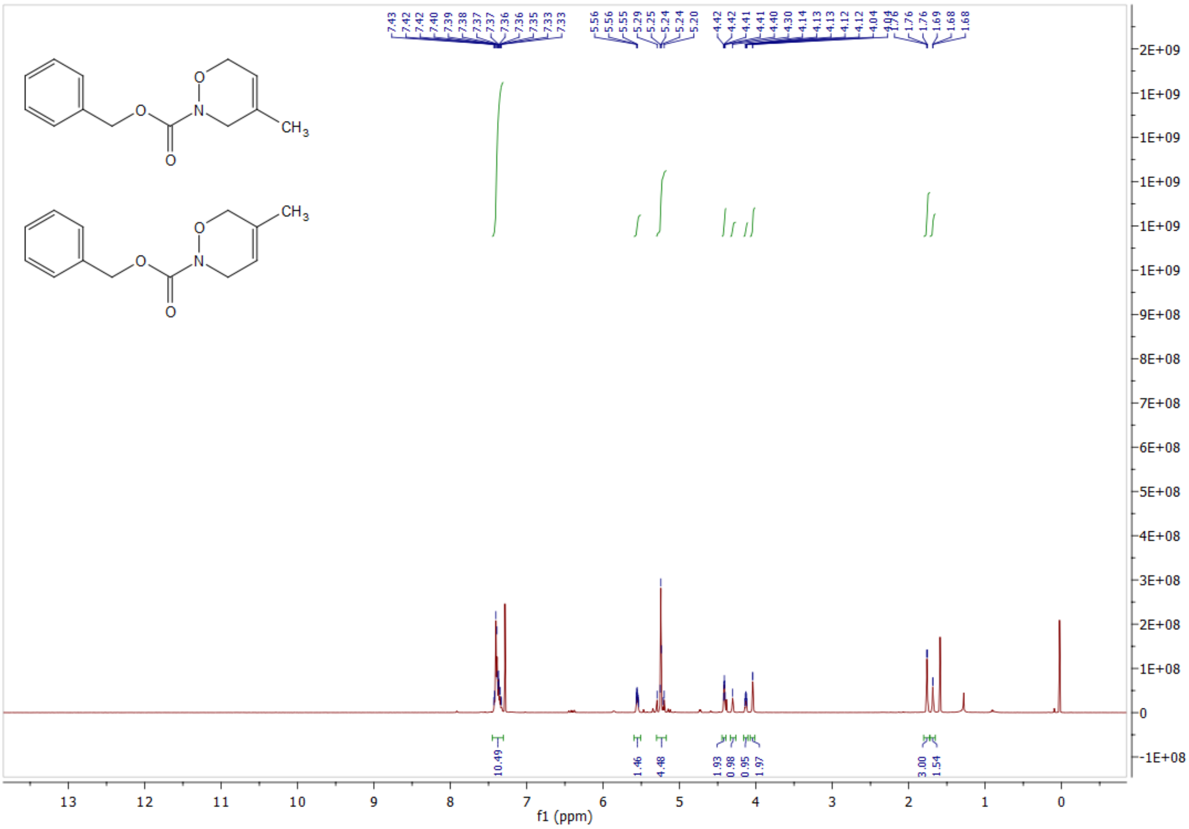
CH3
O
N
O
O
f1 (ppm)

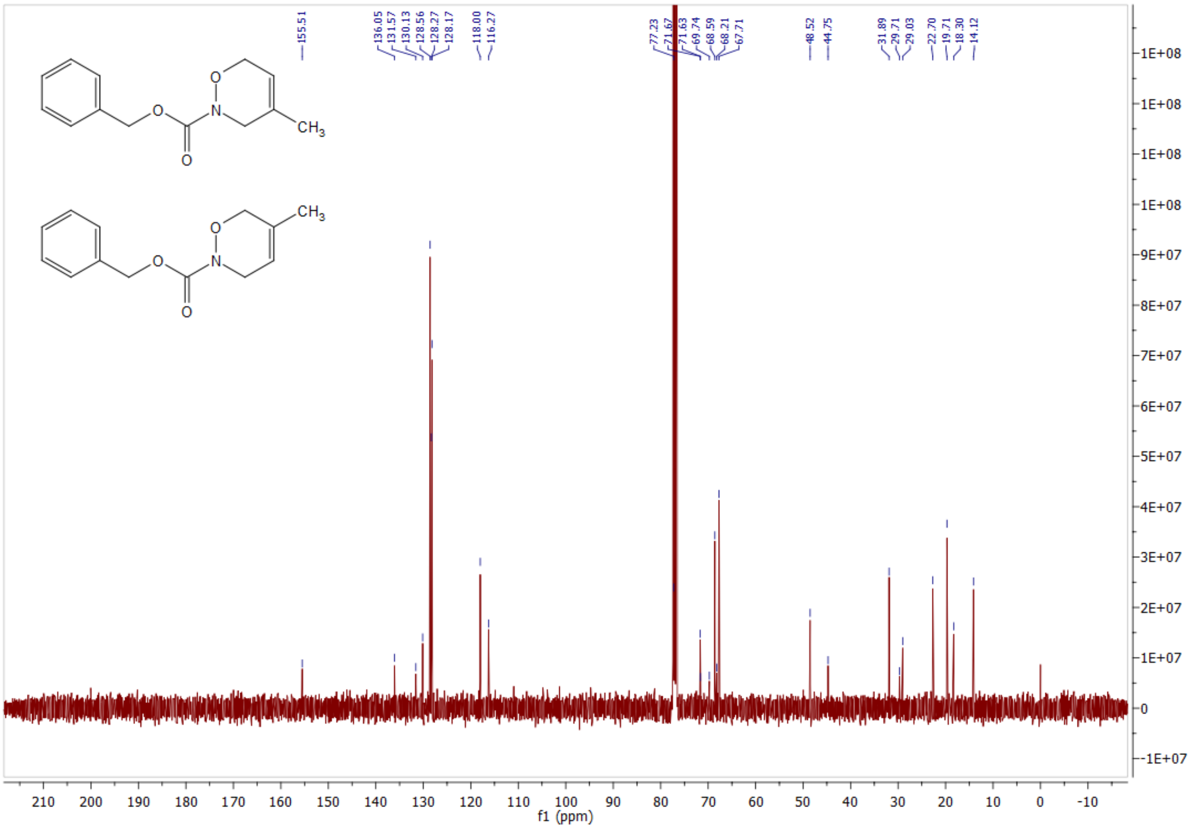
155.51
136.05
131.57
130.13
128.56
128.27
128.17
118.00
116.27
77.23
71.67
71.63
69.74
68.59
68.21
67.71
48.52
44.75
31.89
29.71
29.03
22.70
19.71
18.30
14.12
CH3
f1 (ppm)

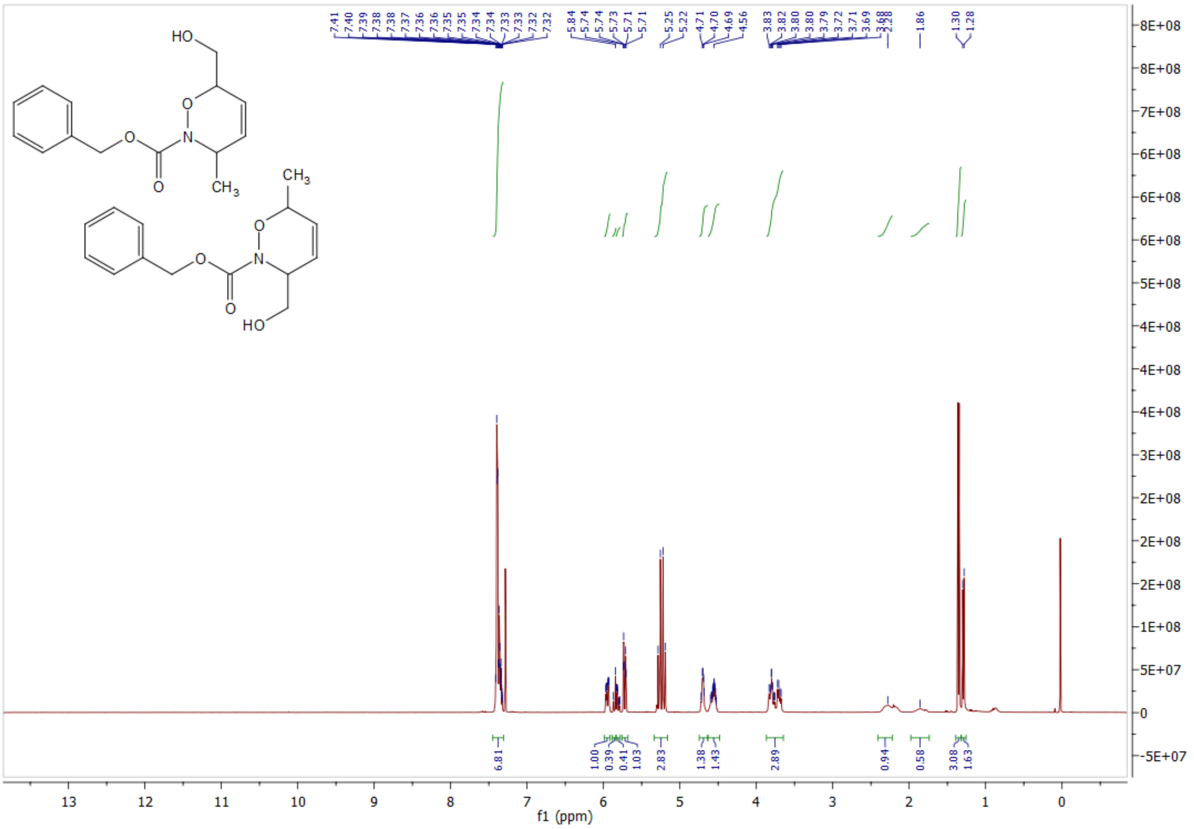
7.41 7.40 7.39 7.38 7.38 7.37 7.36 7.36 7.35 7.35 7.34 7.34 7.33 7.33 7.32 7.32
5.84 5.74 5.74 5.73 5.71 5.71
5.25 5.22
4.71 4.70 4.69 4.56
3.83 3.82 3.80 3.80 3.79 3.72 3.71 3.69 3.68 2.28
1.86
1.30 1.28
HO
O
N
O
O
CH3
CH3
O
N
O
O
HO
6.81
1.00 0.39 0.41 1.03
2.83
1.38 1.43
2.89
0.94
0.58
3.08 1.63
f1 (ppm)

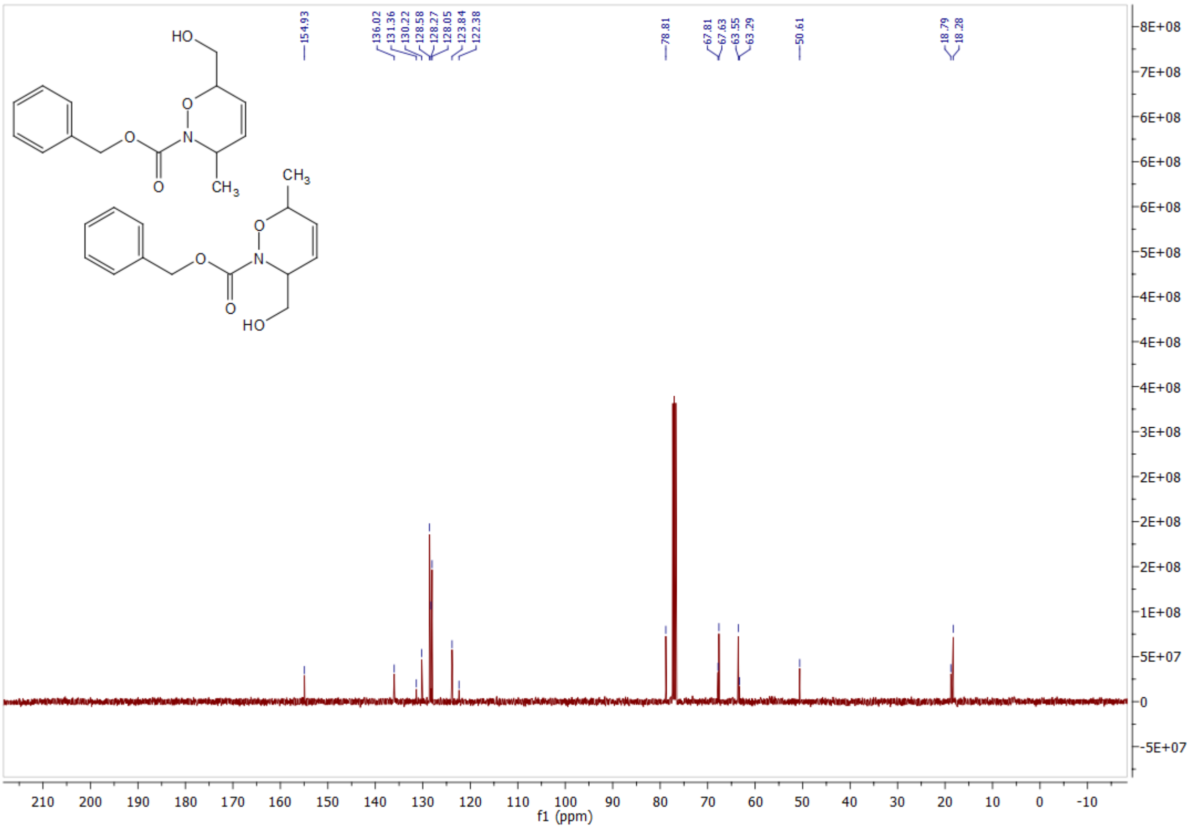
154.93
136.02 131.36 130.22 128.58 128.27 128.05 123.84 122.38
78.81
67.81 67.63 63.55 63.29
50.61
18.79 18.28
HO
O
N
O
O
CH3
CH3
O
N
O
O
HO
f1 (ppm)

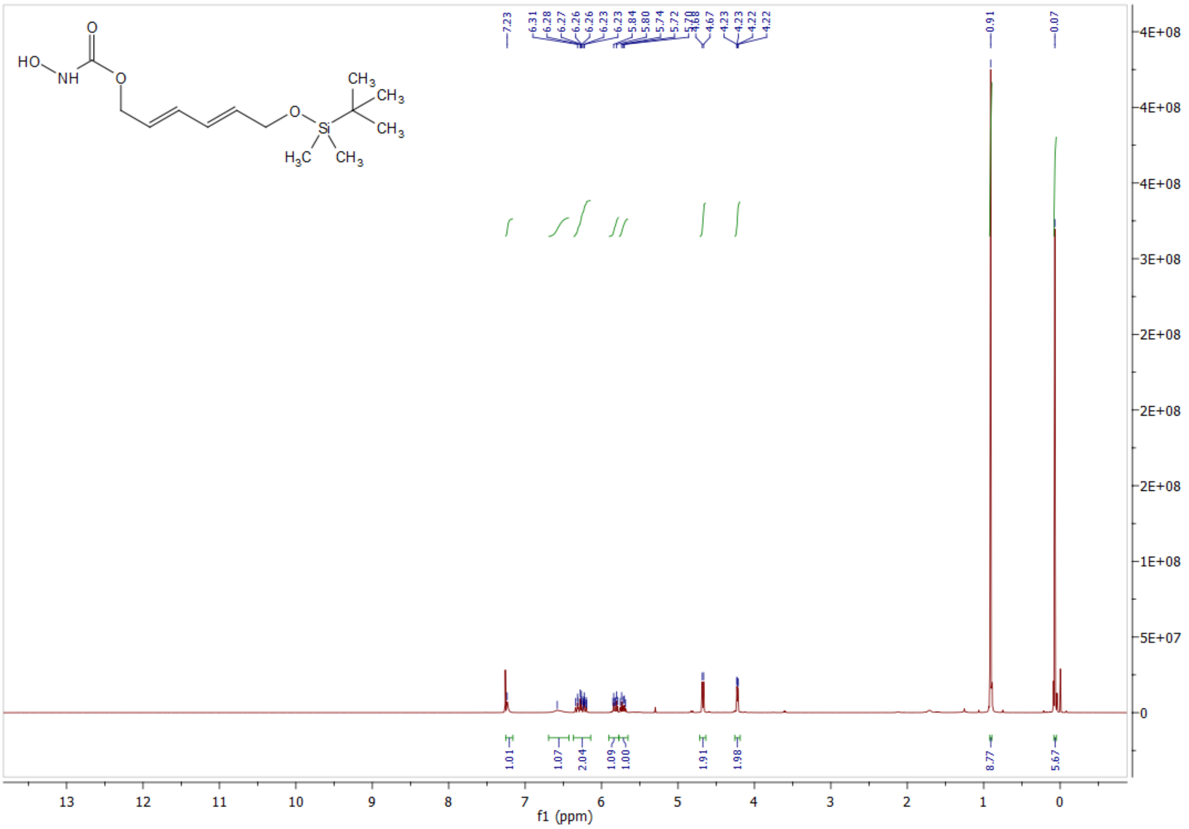
HO
NH
O
O
O
Si
CH3
CH3
CH3
H3C
CH3
7.23
6.31
6.28
6.27
6.26
6.26
6.23
6.23
5.84
5.80
5.74
5.72
5.70
4.68
4.67
4.23
4.23
4.22
4.22
0.91
0.07
1.01
1.07
2.04
1.09
1.00
1.91
1.98
8.77
5.67
f1 (ppm)

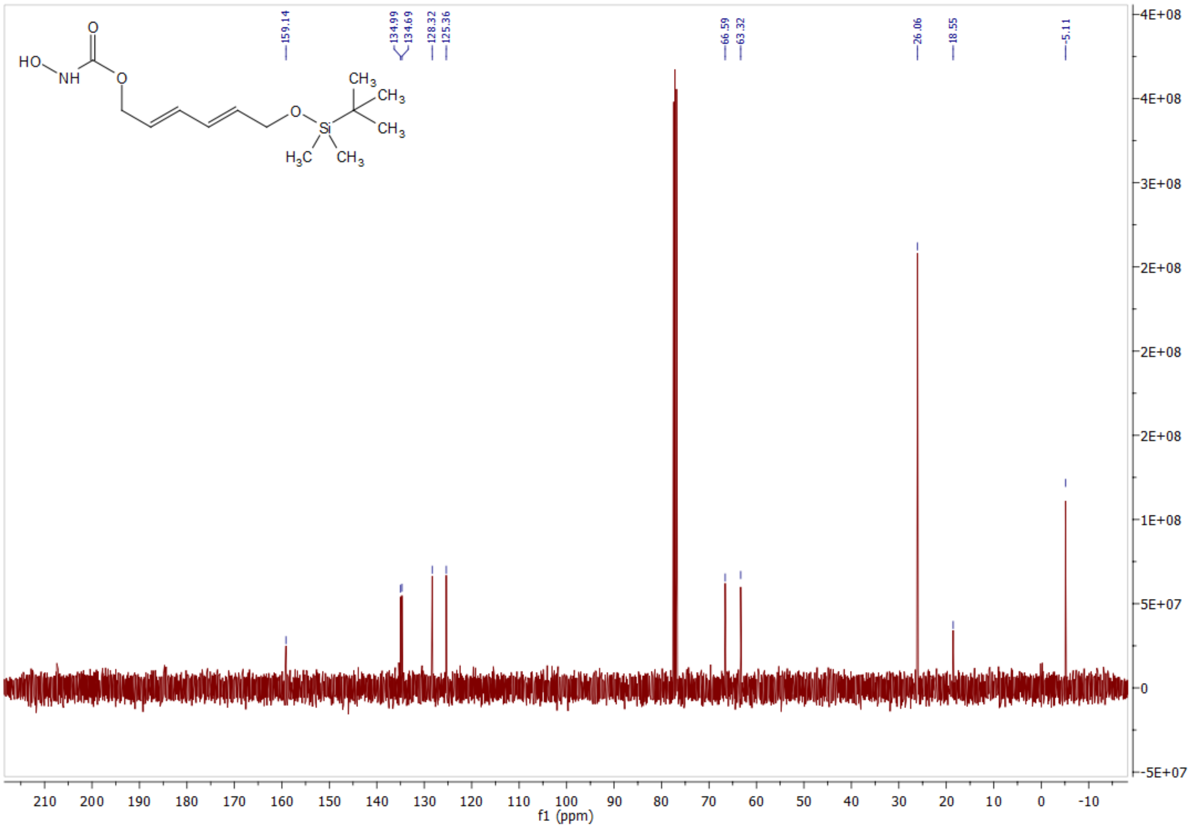
HO
NH
O
O
O
Si
CH3
CH3
CH3
H3C
CH3
159.14
134.99
134.69
128.32
125.36
66.59
63.32
26.06
18.55
-5.11
f1 (ppm)

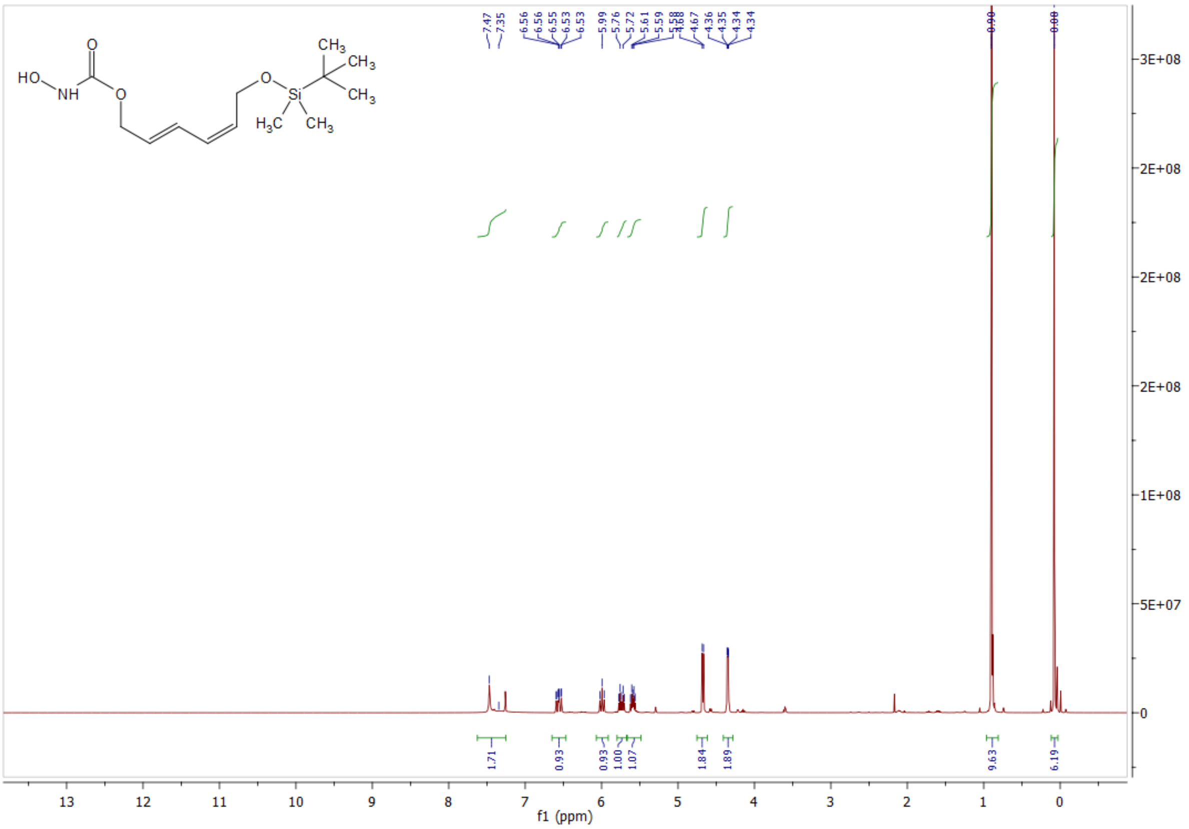

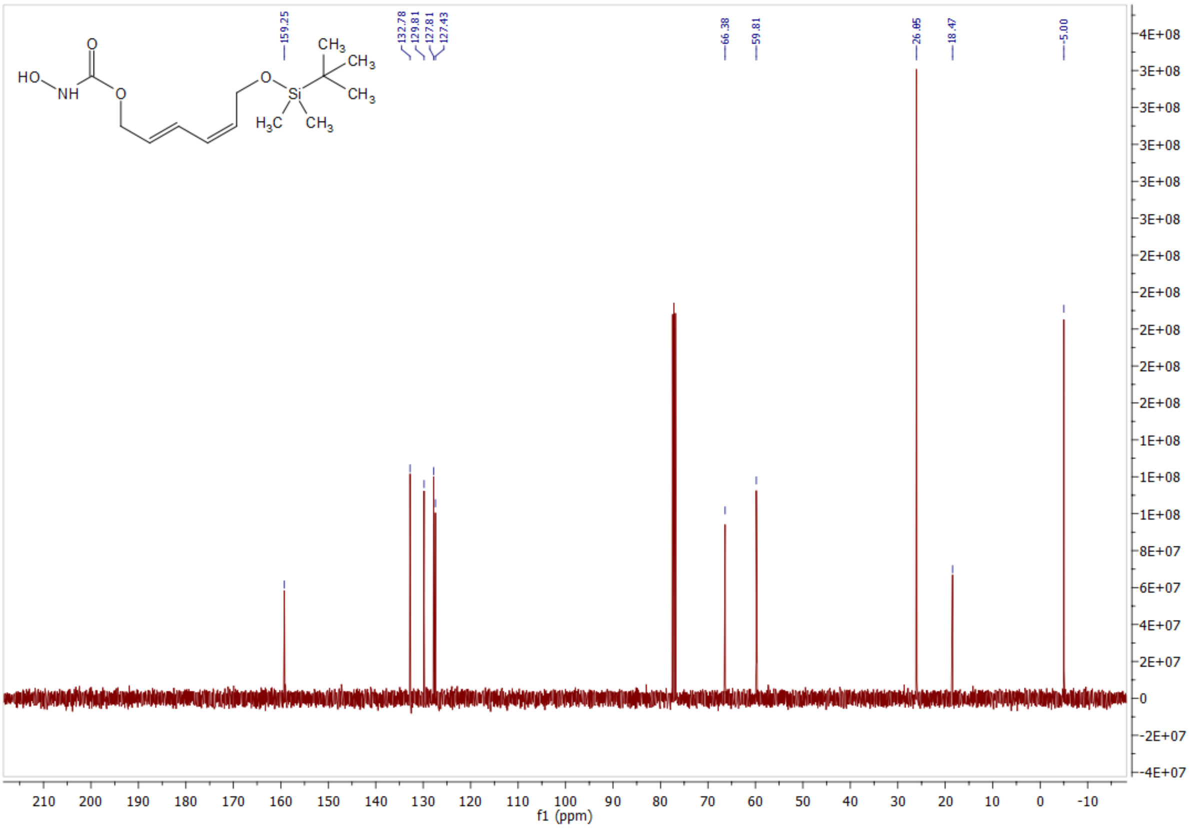

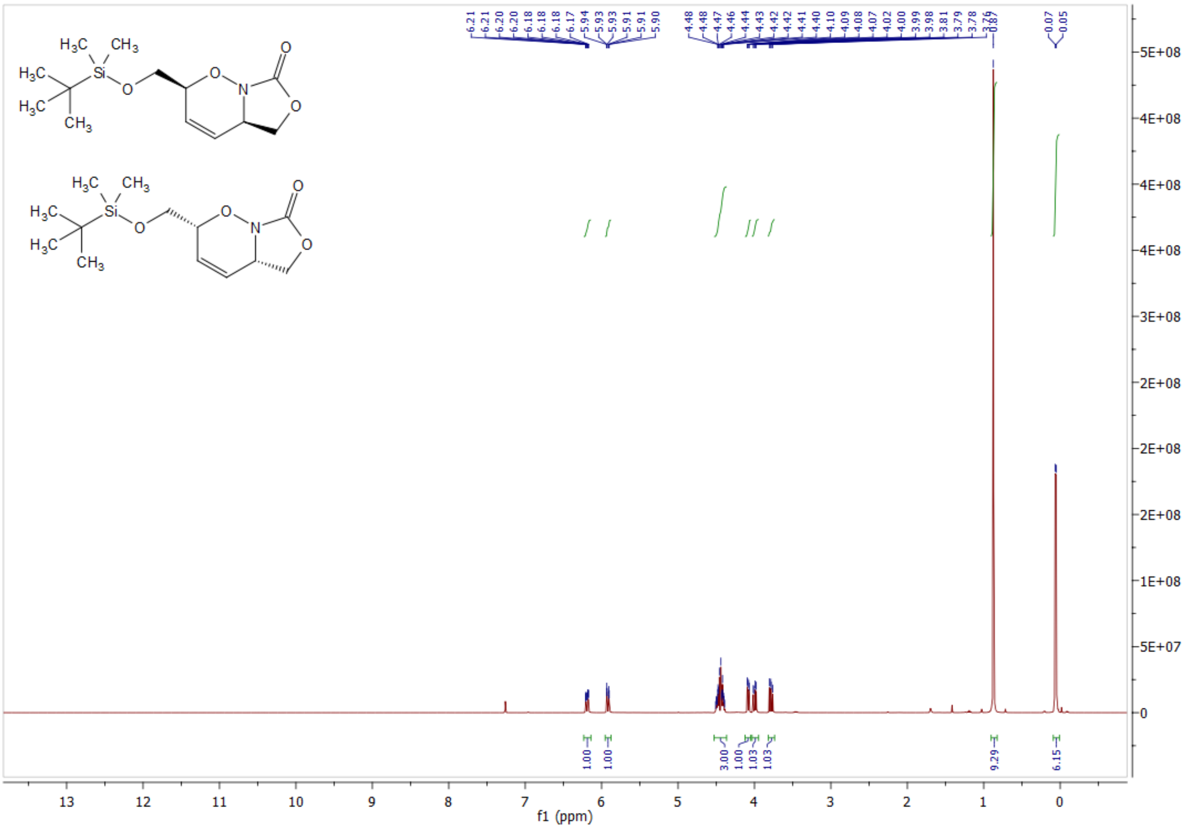

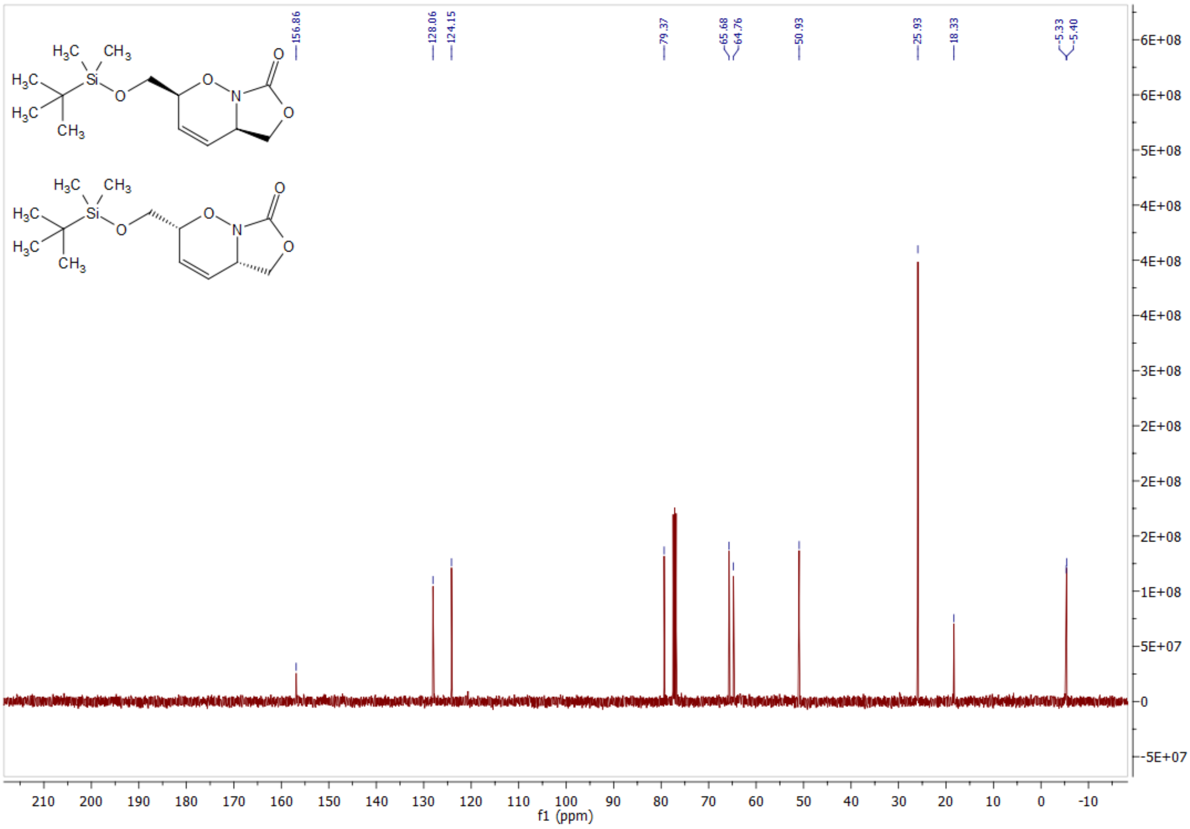

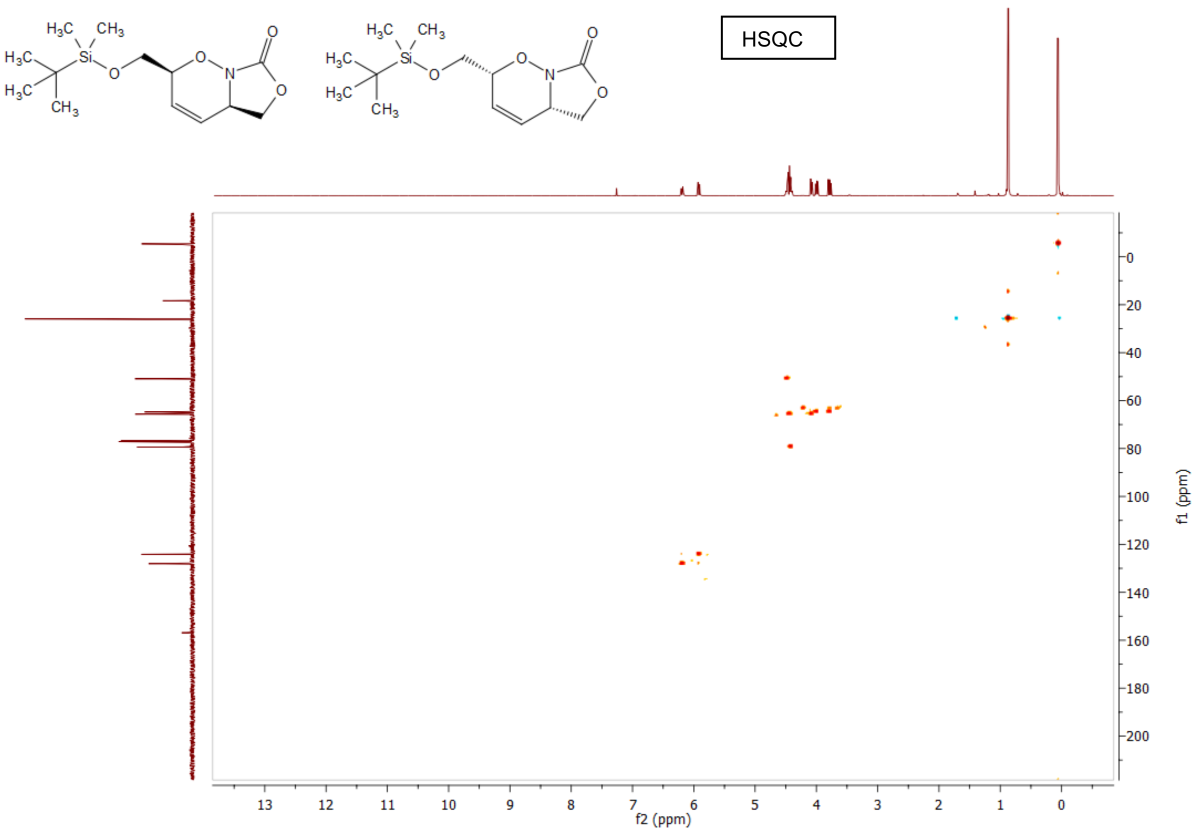
HSQC
f2 (ppm)
f1 (ppm)

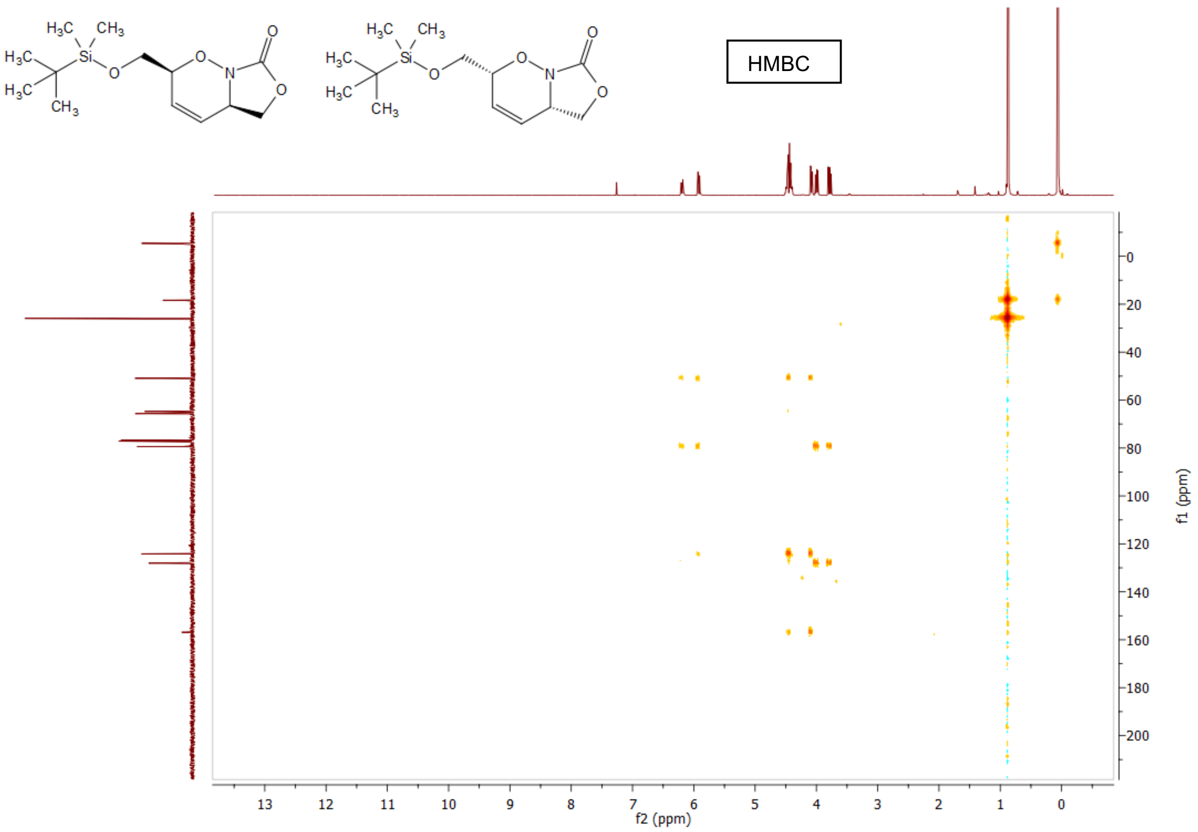
HMBC
f2 (ppm)
f1 (ppm)

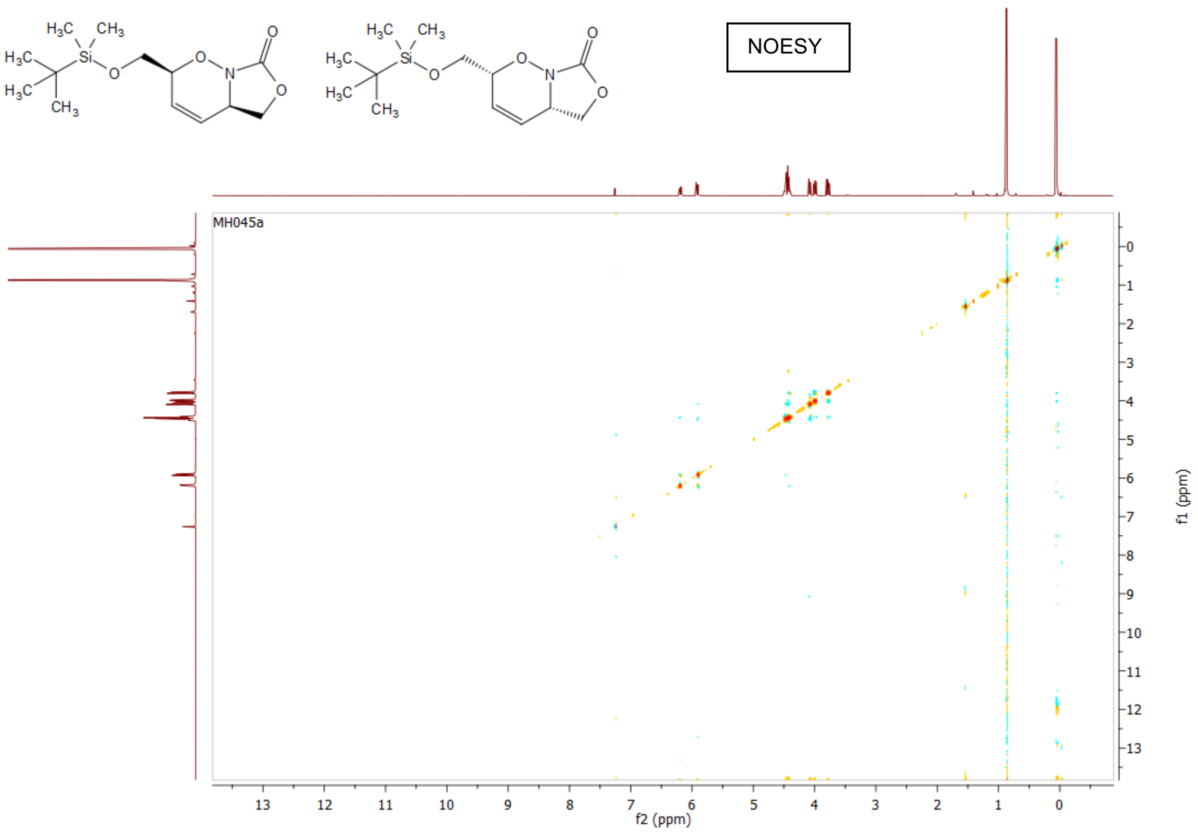
NOESY
MH045a
f2 (ppm)
f1 (ppm)

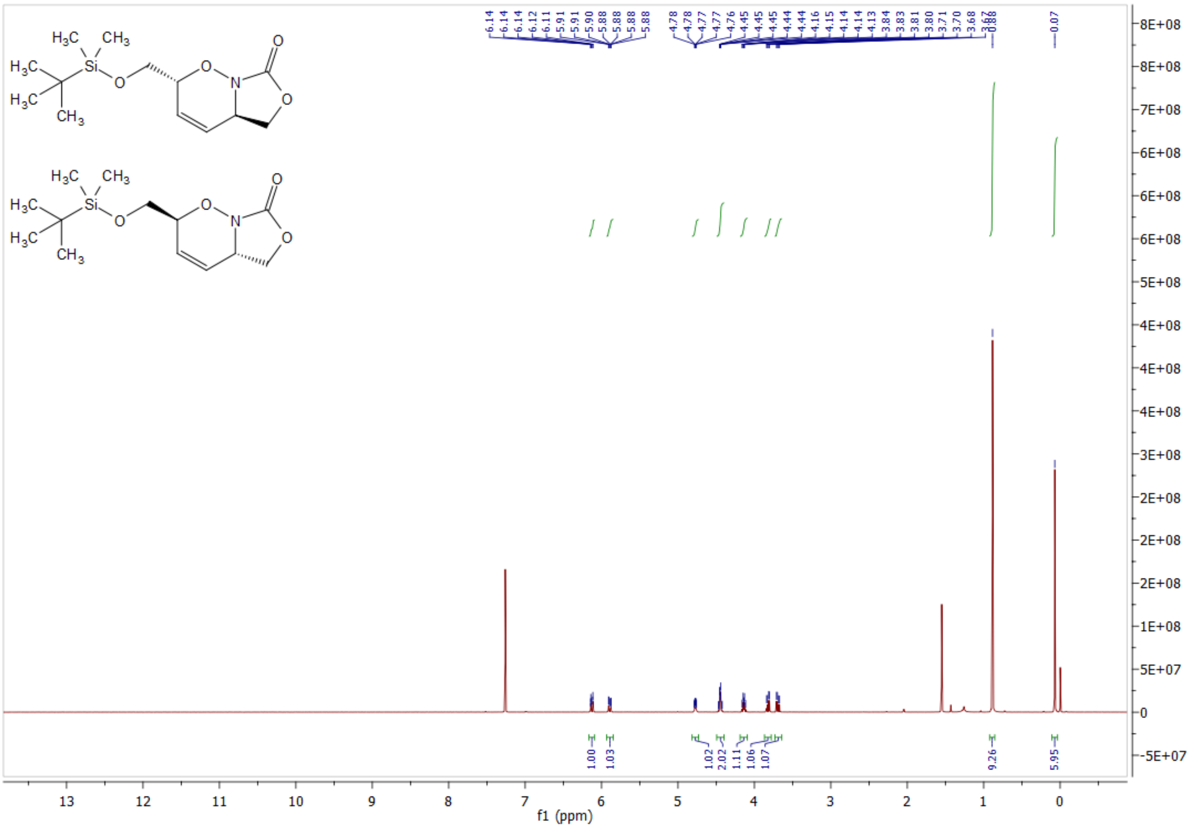
f1 (ppm)

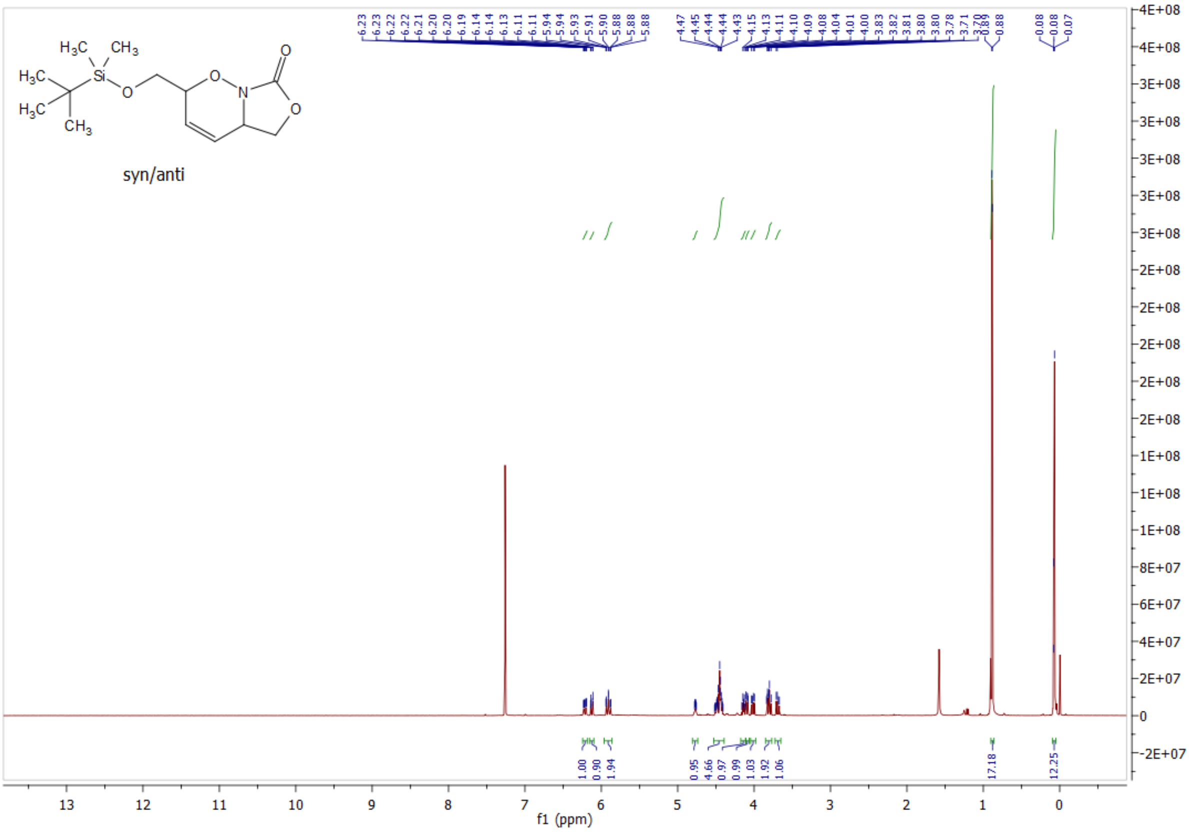

Supplementary references

[1] C. P. Frazier, J. R. Engelking, J. Read de Alaniz, *J. Am. Chem. Soc*., 2011, **133**, 10430.
[2] C. P. Frazier, A. Bugarin, J. R. Engelking, J. Read de Alaniz, *Org. Lett*., 2012, **14**, 3620.
[3] L. Bollans, J. Bacsa, J. A. Iggo, G. A. Morris, A. V. Stachulski, *Org. Biomol. Chem*., 2009, **7**, 4531.
[4] W. Adam, N. Bottke, O. Krebs, C. R. Saha-Möller, *Eur. J. Org. Chem.* 1999, 1963.
[5] D. Atkinson, M. A. Kabeshov, M. Edgar, A. V. Malkov, *Adv. Synth. Catal.* 2011, **353**, 3347.
[5] C. Jäger, M. Haase, K. Korroscheck, V. B. Urlacher, J. Deska, *Angew. Chem. Int. Ed.* 2023, **62**, e202213671.
